# Supplementary material for: Effectiveness of drug interventions to prevent sudden cardiac death in patients with heart failure and reduced ejection fraction: an overview of systematic reviews
Source: BMJ Open. 2018 Jul 28;8(7):e021108. doi: 10.1136/bmjopen-2017-021108 (PMC6067373; doi:10.1136/bmjopen-2017-021108)
Supplement: Supplementary data [file bmjopen-2017-021108supp002.pdf]

Database(s): **Ovid MEDLINE(R) Epub Ahead of Print, In-Process & Other Non-Indexed Citations, Ovid MEDLINE(R) Daily and Ovid MEDLINE(R)** 1946 to 25/05/2017
Search Strategy: 24.05.2017

#	Searches	Results
1	exp heart failure/ or (((heart or cardia* or myocardial) adj3 failure*) or (((heart or cardia*) adj3 decompensation*) or ((heart or cardia*) adj3 insufficienc*))).mp.	189098
2	review.ab.	1050955
3	review.pt.	2299071
4	meta-analysis.ab.	82199
5	meta-analysis.pt.	80375
6	or/2-5	2743700
7	letter.pt.	971739
8	comment.pt.	690989
9	editorial.pt.	439919
10	or/7-9	1585046
11	6 not 10	2708683
12	1 and 11	39715
13	(sudden adj3 (cardiac or death*)).ti,ab,kw.	42355
14	12 and 13	1826

Database(s): **Embase** 1974 to 2017 May 23rd
Search Strategy:

#	Searches	Results
1	exp heart failure/ or (((heart or cardia* or myocardial) adj3 failure*) or (((heart or cardia*) adj3 decompensation*) or ((heart or cardia*) adj3 insufficienc*))).mp.	444143
2	meta-analy:.mp.	202659
3	search:.tw.	417814
4	review.pt.	2246810
5	or/2-4	2642821
6	exp Sudden Cardiac Death/ or Sudden Death/ or exp heart arrest/	107134
7	5 and 6	14446
8	1 and 7	9747
9	(sudden adj3 (cardiac or death*)).mp.	76511
10	limit 9 to abstracts	58853
11	8 and 10	3294
