# Supplementary material for: Effectiveness of drug interventions to prevent sudden cardiac death in patients with heart failure and reduced ejection fraction: an overview of systematic reviews
Source: BMJ Open. 2018 Jul 28;8(7):e021108. doi: 10.1136/bmjopen-2017-021108 (PMC6067373; doi:10.1136/bmjopen-2017-021108)
Supplement: Supplementary data [file bmjopen-2017-021108supp003.docx]

S3 Table: AMSTAR scores for included studies

| Author (year), country | Q 1 | Q 2 | Q 3 | Q 4 | Q 5 | Q 6 | Q 7 | Q 8 | Q 9 | Q 10 | Q 11 | AMSTAR score |
| --- | --- | --- | --- | --- | --- | --- | --- | --- | --- | --- | --- | --- |
| Al-Gobari et al (2013), France | No | No | Yes | No | No | Yes | Yes | Yes | Yes | Yes | No | 6 |
| Chatterjee et al (2013), USA | No | Yes | Yes | Yes | No | Yes | Yes | Yes | Yes | Yes | No | 8 |
| Brophy et al (2001), Canada | No | No | Yes | No | No | Yes | No | No | Yes | Yes | No | 4 |
| Lee et al (2001), USA | No | No | Yes | Yes | No | Yes | No | No | Yes | No | No | 4 |
| Bonet et al (2000), USA | No | Yes | Yes | No | No | Yes | No | No | Yes | No | No | 4 |
| Heidenreich et al (1997), USA | No | Yes | No | Yes | Yes | Yes | No | No | Yes | No | No | 5 |
| Le HH. et al (2016), France | No | Yes | Yes | No | No | Yes | Yes | Yes | Yes | Yes | No | 7 |
| Bapoje et al (2013), USA | No | Yes | Yes | Yes | No | Yes | Yes | Yes | Yes | Yes | No | 8 |
| Wei et al (2010), China | No | Yes | Yes | Yes | No | Yes | No | No | Yes | No | No | 5 |
| Solomon et al (2016), USA | No | Yes | Yes | No | No | Yes | Yes | Yes | yes | No | Yes | 7 |
| Flather et al, (2000), Canada |  |  |  |  |  |  |  |  |  |  |  | NA* |
| Garg et al (1995), Canada | No | Yes | No | No | No | No | No | No | Yes | No | No | 2 |
| Rain & Rada, (2015), Chile* |  |  |  |  |  |  |  |  |  |  |  | NA* |
| Heran et al (2012), Canada | Yes | Yes | Yes | No | Yes | Yes | Yes | Yes | Yes | Yes | Yes | 10 |
| Shibata et al (2008), Canada | No | Yes | No | Yes | No | Yes | No | No | Yes | No | No | 4 |
| Lee et al (2004), USA | No | Yes | Yes | Yes | No | Yes | Yes | No | Yes | Yes | No | 7 |
| Dimopoulos et al (2004), UK | No | Yes | No | No | No | Yes | No | No | Yes | No | No | 3 |
| Jong et al (2002), Canada | Yes | Yes | Yes | No | No | Yes | Yes | Yes | Yes | Yes | No | 8 |
| Rain & Rada, (2017), Chile* |  |  |  |  |  |  |  |  |  |  |  | NA* |
| Al-Gobari et al (2017), Switzerland | No | Yes | Yes | No | No | Yes | Yes | Yes | Yes | Yes | No | 7 |
| Bonsu et al (2015), Malaysia | Yes | Yes | Yes | No | No | Yes | Yes | Yes | Yes | Yes | No | 8 |
| Wang et al (2014), China | No | Yes | Yes | No | No | Yes | Yes | No | No | Yes | No | 5 |
| Liu et al (2014), China | No | Yes | Yes | No | No | Yes | Yes | Yes | Yes | Yes | No | 7 |
| Rahimi et al (2012), UK | No | Yes | Yes | No | No | Yes | Yes | Yes | Yes | No | No | 6 |
| Zhang et al (2011), China | No | Yes | Yes | No | No | Yes | Yes | Yes | Yes | Yes | No | 7 |
| Xu et al (2010), China | No | Yes | Yes | Yes | No | Yes | No | No | Yes | Yes | No | 6 |
| Lipinski (2009), USA | No | Yes | Yes | No | No | Yes | Yes | Yes | Yes | Yes | No | 7 |
| Levantesi et al (2007), Italy | No | Yes | No | No | No | Yes | No | No | Yes | No | No | 3 |
| Claro et al (2015), Chile | Yes | Yes | Yes | Yes | Yes | Yes | Yes | Yes | Yes | Yes | No | 10 |
| Santangeli et al (2012), USA | No | Yes | Yes | Yes | No | Yes | No | No | Yes | No | No | 5 |
| Piccini et al (2009), USA | No | Yes | Yes | No | No | Yes | Yes | Yes | Yes | Yes | No | 7 |
| ATMA Investigators (1997) |  |  |  |  |  |  |  |  |  |  |  | NA* |
| Sim et al (1997), USA | No | Yes | Yes | Yes | No | Yes | No | No | Yes | No | No | 5 |
| Das et al (2010), USA* |  |  |  |  |  |  |  |  |  |  |  | NA* |
| Hilleman et al., (2001), USA* |  |  |  |  |  |  |  |  |  |  |  | NA* |
| Rizos et al (2012), Greece | No | Yes | Yes | No | No | Yes | Yes | Yes | Yes | Yes | Yes | 8 |
| Kotwal et al (2012), Australia | No | Yes | Yes | No | No | Yes | Yes | Yes | Yes | Yes | No | 7 |
| Kwak et al (2012), Korea | No | Yes | Yes | No | No | Yes | Yes | Yes | Yes | Yes | Yes | 8 |
| Chen et al (2011), China | No | Yes | Yes | No | No | Yes | Yes | Yes | Yes | Yes | No | 7 |
| Marik et al (2009), USA | No | Yes | Yes | No | No | Yes | No | No | Yes | No | No | 4 |
| Wang et al (2006), USA | No | No | Yes | Yes | No | Yes | Yes | Yes | Yes | No | No | 6 |

Q 1: Was an 'a priori' design provided?

Q 2: Was there duplicate study selection and data extraction?

Q 3: Was a comprehensive literature search performed?

Q 4: Was the status of publication (i.e. grey literature) used as an inclusion criterion?

Q 5: Was a list of studies (included and excluded) provided?

Q 6: Were the characteristics of the included studies provided?

Q 7: Was the scientific quality of the included studies assessed and documented?

Q 8: Was the scientific quality of the included studies used appropriately in formulating conclusions?

Q 9: Were the methods used to combine the findings of studies appropriate?

Q 10: Was the likelihood of publication bias assessed?

Q 11: Was the conflict of interest included?

* narrative review, overview or individual patient data meta-analysis (not applicable)
