# Supplementary material for: Effectiveness of drug interventions to prevent sudden cardiac death in patients with heart failure and reduced ejection fraction: an overview of systematic reviews
Source: BMJ Open. 2018 Jul 28;8(7):e021108. doi: 10.1136/bmjopen-2017-021108 (PMC6067373; doi:10.1136/bmjopen-2017-021108)
Supplement: Supplementary data [file bmjopen-2017-021108supp004.docx]

S4 Table Funding of included reviews of heart failure patients

| Author (year), country | Review type | Intervention/ comparator | Funding |
| --- | --- | --- | --- |
| Rain and Rada, (2015), Chile | Systematic review | ARB/ ACE-i | No data reported |
| Heran et al (2012), Canada | Systematic review and meta-analysis (Cochrane) | ARB (or ARB + ACE-i) / Placebo ; ACE-i | Included studies: 23 (96%) industry-sponsored and remaining unknown. Review. External: NIHR, UK Cochrane (Non-industry) |
| Shibata et al (2008), Canada | Systematic review and meta-analysis | ARB/ Placebo ; ACE-i | No funding exists. Not reported for included studies. |
| Lee et al (2004), USA | Meta-analysis | ARB/ Placebo ; ACE-i | Industry support: Stock ownership or options (other than mutual funds): D.C. Rhew (Merck, Pfizer); Grants received: Zynx Health Incorporated has received grants from AstraZeneca, Aventis,  Bristol-Myers Squibb, Merck, Novartis, and Pfizer. |
| Dimopoulos et al (2003), UK | Meta-analysis | ARB/ Placebo ; ACE-i | No data reported |
| Jong, et al (2002), Canada | Systematic review and meta-analysis | ARB (or ARB + ACE-i) / Placebo ; ACE-i | No data reported |
| Garg et al (1995), Canada | Systematic review & meta-analysis | ACE-i / Control | No data reported |
| Flather et al (2000), Canada | Systematic review | ACE-i / Placebo | Industry support: Medical Research Council of Canada and support from Merck Frosst Canada, Merck Sharpe and  Dohme, USA, Bristol Myers, Squibb, USA, Zeneca, UK, and Hoechst Marion Roussel, Frankfurt, Germany. |
| Le et al (2016), France | Systematic review and meta-analysis | Anti-aldosterone / Placebo; usual care | Non-industry (Academic and Government) |
| Bapoje et al (2013), USA | Systematic review and meta-analysis | Anti-aldosterone / Placebo; usual care | Not reported for included studies. No external fund for the review. |
| Wei J., et al (2010), China | Meta-analysis | Anti-aldosterone / Placebo; usual care | No data reported |
| Chatterjee et al (2013), USA | Systematic review and meta-analysis | Beta-blockers / Placebo; beta-blocker; usual care | None |
| Al-Gobari et al (2013), France | Systematic review & meta-analysis | Beta-blockers /Placebo; usual care | YEMEN LNG company: Private company: non-drug-industry., UMR 5558, BEBL. |
| Brophy et al (2001), Canada | Meta-analysis | Beta-blockers / Placebo; usual care | Les Fonds de la Recherche en Santé du Québec. Government (non-industry) |
| Lee et al (2001), USA | systematic review and meta-analysis | Beta-blockers / Placebo | No data reported |
| Bonet et al (2000), USA | Meta-analysis | Beta-blockers / Placebo ; usual care | No data reported |
| Heidenreich et al (1997), USA | Meta-analysis | Beta-blockers / Placebo; usual care | No data reported |
|  |  |  |  |
| Rizos et al (2012), Greece | systematic review and meta-analysis | Omega 3 Fatty acids / Placebo ; usual care | Funding of included studies is reported. Conflict of interest too ( for one author): industry sponsorship not related to those who manufacture or market Omega-3 suppl. |
| Kotwal et al (2012), Australia | systematic review and meta-analysis | Omega 3 Fatty acids / Placebo ; usual care | Reported but not for included studies. Non-industry. |
| Kwak et al (2012), Korea | Meta-analysis | Omega 3 Fatty acids / Placebo ; usual care | All included trials sponsored by industry but not reported for the review itself. |
| Chen et al (2011), China | Meta-analysis | Omega 3 Fatty acids / Placebo ; usual care | No data reported |
| Marik et al (2009), USA | systematic review | Omega 3 dietary supplements / Placebo; olive oil ; corn oil, sunflower oil ; usual care | No data reported |
| Wang et al (2006), USA | systematic review | n-3 Fatty acids / Placebo/olive oil ; corn oil, sunflower oil ; usual care | Internal by saying who obtained funds but not from whom. No data for included studies. |
| Solomon et al (2016), USA | Meta-analysis | Sacubitril; valsartan / Lisinopril; enalapril | Data reported for included studies: industry. No funding existed for the review itself. |
| Rain and Rada (2017), Chile | systematic review | Statins / Placebo ; usual care | No data reported |
| Al-Gobari et al (2017), Switzerland | Systematic review and meta-analysis | Statins / Placebo ; usual care | Yes for the review: non-industry (Governments). But not reported for included studies. |
| Bonsu et al (2015), Malaysia | Meta-analysis | Statins / Placebo ; usual care | No data reported |
| Wang et al (2014), China | Meta-analysis | Statins / Placebo ; usual care | None (reported) but not for included studies. |
| Liu et al (2014), China | Meta-analysis | Statins / Placebo ; usual care | Non-industry-sponsored but no data for included studies. |
| Zhang et al (2011), China | Meta-analysis | Statins / Placebo ; usual care | Non-industry-sponsored but no data for included studies. Academic, doctoral program…etc |
| Xu et al (2010), China | Meta-analysis | Statins / Placebo ; usual care | No data reported |
| Lipinski et al (2009), USA | Meta-analysis | Statins / Placebo ; usual care | No data reported |
| Levantesi et al (2007), Italy | Meta-analysis | Statins / Placebo ; usual care | No data reported |
| Rahimi et al (2012), UK | Meta-analysis | Statins / Placebo ; usual care | Funding for included studies reported. Some are industry. No funding for the review itself from industry but from Academy for some authors. |
| Claro et al (2015), Chile | Systematic review and meta-analysis (Cochrane) | Amiodarone / control | Funding for the review: Government (non-industry). But not reported for included studies. |
| Santangeli et al (2012), USA | systematic review | Amiodarone / Placebo | No data reported |
| Piccini et al (2009), USA | Meta-analysis | Amiodarone / Placebo ; control | No external fund for the review. No data reported for included studies. |
| ATMA Investigators (1997) | Meta-analysis | Amiodarone / Placebo ; usual care | No data reported |
| Sim et al (1997), USA | Meta-analysis | Amiodarone / Placebo ; usual care | No data reported |
| Das et al (2010), USA | Narrative Review | Anti-arrhythmics / Placebo ; usual care | No data reported |
| Hilleman et al (2001), USA | Narrative Review | Anti-arrhythmics / Placebo ; usual care | No data reported |
