# Supplementary material for: Effectiveness of drug interventions to prevent sudden cardiac death in patients with heart failure and reduced ejection fraction: an overview of systematic reviews
Source: BMJ Open. 2018 Jul 28;8(7):e021108. doi: 10.1136/bmjopen-2017-021108 (PMC6067373; doi:10.1136/bmjopen-2017-021108)
Supplement: Author's manuscript [file bmjopen-2017-021108.draft_revisions.pdf]

BMJ Open

BMJ Open is committed to open peer review. As part of this commitment we make the peer review history of every article we publish publicly available.

When an article is published we post the peer reviewers' comments and the authors' responses online. We also post the versions of the paper that were used during peer review. These are the versions that the peer review comments apply to.

The versions of the paper that follow are the versions that were submitted during the peer review process. They are not the versions of record or the final published versions. They should not be cited or distributed as the published version of this manuscript.

BMJ Open is an open access journal and the full, final, typeset and author-corrected version of record of the manuscript is available on our site with no access controls, subscription charges or pay-per-view fees (http://bmjopen.bmj.com).

If you have any questions on BMJ Open's open peer review process please email info.bmjopen@bmj.com

BMJ Open

Effectiveness of drug interventions to prevent sudden cardiac death in patients with heart failure and reduced ejection fraction: an overview of systematic reviews

Journal:	*BMJ Open*
Manuscript ID	bmjopen-2017-021108
Article Type:	Research
Date Submitted by the Author:	11-Dec-2017
Complete List of Authors:	Al-Gobari, Muaamar; Lausanne university Hospital (CHUV), Institute of social & preventive medicine (IUMSP), Cochrane Switzerland<br>Al-Aqeel, Sinaa; King Saud University , Clinical Pharmacy<br>Gueyffier, François; Université Claude Bernard Lyon 1, UMR5558; Hospices Civils de Lyon, Service de Pharmacologie clinique<br>Burnand, Bernard; Lausanne university Hospital (CHUV), Institute of social & preventive medicine (IUMSP), Cochrane Switzerland
Keywords:	cardiac failure, sudden death, umbrella review, meta-analysis, guidelines, treatment

SCHOLARONE™ Manuscripts

For peer review only

Effectiveness of drug interventions to prevent sudden cardiac death in patients with heart failure and reduced ejection fraction: an overview of systematic reviews

Muaamar Al-Gobari[1*], Sinaa Al-Aqeel[2], François Gueyffier[3], Bernard Burnand[1]

[1] Institute of social & preventive medicine (IUMSP) and Cochrane Switzerland, Lausanne University Hospital (CHUV), Lausanne, Switzerland

[2] Clinical Pharmacy Department, College of Pharmacy, King Saud University, Riyadh, Kingdom of Saudi Arabia.

[3] Laboratoire de Biologie et Biométrie Evolutive-Equipe Modélisation des Effets Thérapeutiques, UMR 5558 Université Claude Bernard Lyon1, Lyon, France

*** Corresponding Author Information:**

Institute of social & preventive medicine (IUMSP)

Biopôle 2, Route de la Corniche 10

CH-1010 Lausanne, Switzerland

Tel. +41 21 314 51 45

E-mail: muaamar.algobari@gmail.com

For peer review only

For peer review only - http://bmjopen.bmj.com/site/about/guidelines.xhtml

Abstract

Objectives

To summarize and synthesize the current evidence regarding the effects of drug interventions on prevention of sudden cardiac death (SCD) and all-cause mortality in heart failure with reduced ejection fraction (HFrEF) patients.

Design

Overview of systematic reviews.

Data sources

Medline, Embase, ISI Web of Science and Cochrane Library from inception to May 2017; manual search of references of included studies for potentially relevant reviews.

Eligibility criteria for study selection

We reviewed drug interventions in HFrEF patients that evaluated the effectiveness on SCD and all-cause mortality prevention, including overviews, systematic reviews and meta-analysis of randomized controlled trials of beta-blockers, angiotensin-converting enzyme inhibitors (ACE-i), angiotensin receptor blockers (ARBs), anti-aldosterones or mineralocorticoid-receptor antagonists, amiodarone, other antiarrhythmics, combined angiotensin receptor blocker (ARB)/neprilysin inhibitors, statins and fish oil supplementation.

Review methods

Two independent reviewers extracted data and assessed the methodological quality of the reviews and the quality of evidence for the primary studies for each drug intervention, using AMSTAR and GRADE respectively.

Results

We identified 41 reviews. Beta-blockers, anti-aldosterones, and combined angiotensin receptor blocker (ARB)/neprilysin inhibitors appeared effective to prevent SCD and all-cause mortality, angiotensin-converting enzyme inhibitors significantly reduced all-cause mortality but not SCD events, angiotensin receptor blockers and statins were ineffective where antiarrhythmics and omega-3 fatty acids had unclear evidence of effectiveness for prevention of SCD and all-cause mortality.

For peer review only

For peer review only - http://bmjopen.bmj.com/site/about/guidelines.xhtml

Conclusions

This comprehensive overview of the evidence confirms that beta-blockers, anti-aldosterone agents and combined ARB/neprilysin inhibitors are effective on SCD prevention but not ACE-i or ARBs. In high-risk SCD patients, a shift from ACE-i to a combined ARB/neprylisin inhibitor is an option.

Systematic Review Registration: PROSPERO 2017: CRD42017067442

Key Words
cardiac failure, sudden death, umbrella review, treatment, pharmacotherapy, meta-analysis, guidelines, evidence-based medicine

For peer review only

Strengths and limitations of this study

- A major strength of our study is that it summarizes and synthesizes the effectiveness of most evidence-based drug interventions in heart failure patients with reduced ejection fraction for sudden cardiac death (SCD) prevention and classified drug interventions according to the current evidence of their effectiveness.
- Our study used data from published studies and no data from unpublished studies.
- Our study reviews most cardiovascular drugs on the prevention of SCD and all-cause mortality but limited in scope for not including non-drug devices such as implantable cardioverter defibrillators.

For peer review only - http://bmjopen.bmj.com/site/about/guidelines.xhtml

Introduction

Heart failure (HF) morbidity and mortality constitute an important burden for patients and for the health-care systems in both developed and developing countries [1]. HF patients are frequently hospitalized and not only have a high mortality risk from a bad prognosis but also unexpectedly, termed sudden cardiac death (SCD). In people diagnosed with HF, SCD occurs at 6–9 times the rate of the general population and that almost 20% of patients die within one year of initial diagnosis and more than 80% on 8-year mortality [1 2]. Potential drug interventions in heart failure with reduced ejection fraction (HFrEF) patients include beta-blockers (BBs), angiotensin-converting enzyme inhibitors (ACE-i), angiotensin receptor blockers (ARBs), anti-aldosterones or mineralocorticoid-receptor antagonists, amiodarone, other antiarrhythmics, combined angiotensin receptor blocker/neprilysin inhibitors, statins and fish oil supplementation [3]. Some of these interventions aimed at improving survival and reducing total mortality and SCD in HF. For instance, a newly-licensed drug (Sacubitril/valsartan) in Paradigm-HF trial [4] showed around 20% SCD reduction compared to enalapril.

Nevertheless, current strategies to prevent SCD in HF appear suboptimal if we take into account the high portion of mortality that still occur in this population. Had a practitioner identified a patient with high risk of SCD, it would be important to know which drug is effective or not in SCD prevention. However, the large amount of information and the multiple and sometimes discordant systematic reviews could be misleading [5]. Moreover, the prescription of poly-drug regimen is usually adopted [3].

Therefore, there is a need to further review and investigate if all these drug interventions prevent SCD events in high-risk patients and whether one treatment is preferable to another or if any optimization of therapeutic strategies to those patients is possible accordingly. Thus, we decided to conduct an overview of systematic reviews to summarize and synthesize the available evidence of the effectiveness of drug interventions in the prevention of SCD in HFrEF and categorized the evidence into effective, ineffective and unclear evidence of effectiveness.

Methods

We developed a priori protocol for this review according to the Preferred Reporting Items for Systematic Reviews and Meta-Analyses (PRISMA) statement (**S1 File**) and registered it on the PROSPERO International prospective register of systematic reviews (CRD42017067442).

For peer review only - http://bmjopen.bmj.com/site/about/guidelines.xhtml

Data sources and search strategy

Using the Ovid online interface, we searched Medline (up to May 24, 2017), Embase (up to May 23, 2017), ISI web of science and the Cochrane library (up to May 24, 2017). We identified overviews, systematic reviews and meta-analyses of randomized clinical trials by means of a search strategy (available on **S2 File**). The search strategy was composed of a filter [6 7], a mixture of medical subject heading terms [Mesh and EMTREE in Medline and Embase respectively], text words as well as a truncation when possible without any language or publication date restriction. We did not search conference proceedings nor the grey literature. Reference lists of the included reviews were manually checked for any additional eligible studies. We contacted corresponding reviews and primary studies' authors in the case of relevant unreported data. If judged necessary, we intended to update the included reviews by searching primary studies published after the systematic review publication date. Apart from authors' expertise in the field, we decided to update if the most up-to-date review of a drug intervention was published more than 5 years ago and/or new clinical trials are not integrated into the evidence.

Selection criteria and data abstraction

Studies were eligible if they were overviews, systematic reviews and meta-analyses of randomized clinical trials that evaluated the effectiveness of drug interventions in HFrEF patients. Reviews were included if examined the effectiveness of the following drugs: beta-blockers (BBs), angiotensin-converting enzyme inhibitors (ACE-I), angiotensin receptor blockers (ARBs), anti-aldosterones or mineralocorticoid-receptor antagonists, amiodarone, antiarrythmics, angiotensin receptor blocker/neprilysin inhibitor, statins and fish oil supplementation. The selected reviews should have contained at least one of the aforementioned HF therapy and had evaluated SCD and/or all-cause mortality prevention as outcomes. We used Endnote and Rayyan[8] to remove duplicates during the selection based on titles and abstracts, and full-text screening.

The abstracted data included eligibility criteria, population type, ejection fraction, study design (including intervention and comparator arms), follow-up duration, and authors' evaluation of outcomes.. Two reviewers (MA&SA) independently abstracted data. We resolved discrepancies by consensus or by adding a third reviewer judgment, when necessary.

Quality assessment of included reviews

Methodological quality of included reviews

Two authors independently used the AMSTAR measurement tool to assess systematic reviews included in our overview. The AMSTAR checklist comprises eleven questions (**S3 table**) and each question accounted for one score point [9]. The answer of "yes" gave a score of 1 and zero otherwise. This increasingly adopted tool was

For peer review only - http://bmjopen.bmj.com/site/about/guidelines.xhtml

used at the data collection step as stipulated in the overview protocol [10]. Should the authors of included reviews failed to publish their protocol, we deducted one score. In addition, we scored "yes" if the authors mentioned that two reviewers were involved in the study screening, selection or data extraction.

Quality of evidence in included reviews

Two authors independently used the GRADE (Grading of Recommendations, Assessment, Development and Evaluation) approach [11] to assess the quality of evidence of each intervention[11]. GRADE is a widely accepted tool that allows the assessment of five key elements: risk of bias, inconsistency, indirectness, imprecision, and publication bias. GRADE categorizes the quality of evidence into four levels: high, moderate, low and very low. In presence of a high risk of bias, the quality of the evidence is downgraded from high to moderate and so on. We also reported the GRADE assessments reported by the authors of the included reviews, or assessed them otherwise. Moreover, we did not reassess the risk of bias at primary study level if authors of included reviews had sufficiently assessed their quality. In the case of the updated review for angiotensin receptor blockers, however, we assessed the quality of newly added randomized clinical trials and integrated it into the evidence synthesis.

Statistical analysis and data synthesis

We provided a narrative synthesis of the findings for the included reviews and if multiple reviews existed. However, in the case of ARBs, we updated the evidence and meta-analyzed the data using random-effects and fixed-effects model with Mantel-Haenszel methods [12]. Meanwhile, we evaluated each intervention against our outcomes of interest and synthesized the evidence taking into account heterogeneity and inconsistencies between reviews.

For the purpose of our overview, we categorized the evidence of the included interventions into three categories: 1. effective interventions; 2. ineffective interventions; 3. uncertain evidence (conflicting or inconclusive evidence). We used relative effect [odds ratio (OR) or risk ratio (RR)] as a summary statistic from the most recent or largest published systematic reviews, and confidence intervals (CIs) of 95% with a significance level determined at two-sided alpha less than 5%.

Results

Search result

According to our pre-defined eligibility criteria, our search strategy in electronic databases and manual searches resulted into 41 studies [5 13-52]. Fig 1 shows the search strategy results.

For peer review only - http://bmjopen.bmj.com/site/about/guidelines.xhtml

Characteristics of included reviews

As shown in **Table 1**, the population of the included reviews consisted of heart failure patients with an ejection fraction ≤ 45% in most studies and a corresponding New York Heart Association (NYHA) classification ranging from I-IV. The effectiveness of each drug intervention has been assessed in at least one review. All reviews were systematic except two reviews for anti-arrhythmic drugs. At the time of their publication, 15 out of 41 reviews (37%) had corresponding authors based on the USA, 7 (17%) from Canada, 6 (15%) from China, 3 from Chile, 2 from France, 2 from UK, and the 6 remaining from other countries.

The disclosure and reporting of financial resources or funding varied from one study to another. Twenty-one reviews (51%) did not report the source of funding. Ten reviews (24 %) reported support that included governments, academic institutions, and device industry. Six reviews declared financial resources as none or no external funds. Three reviews reported industry sponsorship for at least one author. One review [51] stated that one author obtained funds for the review without clarifying the source (see **S4 table**). We also reported authors' findings summary for each review as stated by their respective authors.

For peer review only

Table 1: Summary of characteristics of included studies of heart failure patients

Author (year); country; reference	Review type	Intervention/ Comparator	Population type; Ejection fraction (%); NYHA	Study design n; participants n	Mean follow-up/Range (months)	Authors' Findings summary	AMSTAR score
Al-Gobari M., et al. (2013), France [14]	Systematic review & meta-analysis	Beta-blockers /Placebo; "usual care"	HF; <45% except one study < 62%; I-IV	RCTs n= 30; n = 24,779	Mean: 11.51	Beta-blockers significantly reduced SCD, cardiovascular death and all-cause mortality	6
Chatterjee S., et al. (2013); USA [13]	Systematic review & meta-analysis	Beta-blockers / Placebo; beta-blocker; "usual care"	HF; <45%; II-IV	RCTs n=21 ; n= 23,122	median: 12	The study confirmed mortality benefits of BBs compared to placebo or usual care in heart failure with reduced ejection fraction.	8
Brophy JM. Et al., (2001); Canada [15]	Meta-analysis	Beta-blockers / Placebo; "usual care"	CHF; <45%; I-IV	RCTs n= 22 ; n= 10,135	Range: 3 to 23	This study reported a reduction in mortality and morbidity in CHF.	4
Lee S. et al., (2001), USA [16]	systematic review & meta-analysis	Beta-blockers / Placebo	HF; <30%; II-III	RCTs n=6 ; n= 9335	Range : 00000	The authors recommended use of beta-blockers in HF with reduced ejection fraction and NYHA II-III.	4
Bonet S., et al. (2000), USA [17]	Meta-analysis	Beta-blockers / Placebo ; "usual care"	HF; <45%; NA	RCTs n= 21 ; n= 5,849	Median: 6	Beta-blockers reduce total mortality by reducing pump failure and sudden cardiac death events. Vasodilating beta-blockers have perhaps greater effects on overall mortality than nonvasodilating agents.	4
Heidenreich P.A., et al. (1997); USA [18]	Meta-analysis	Beta-blockers / Placebo; "usual care"	HF; <30%; I-IV	RCTs n= 17 ; n= 3,039	Range: 3 to 24	Beta-blockers significantly reduced all-cause mortality but showed a trend toward better reduction in nonsudden cardiac death compared to sudden cardiac death.	5
Le HH. et al., (2016); France [19]	Systematic review & meta-analysis	Anti-aldosterone / Placebo; "usual care"	HF, post-MI; <40%- >50%; I-IV	RCTs n=25; n = 19,333	Range: 3 to 39.6	In HF, anti-aldosterones or mineralocorticoid receptor blockers reduced SCD, all-cause mortality and cardiovascular, all-cause and cardiovascular hospitalization. Adverse effects (hyperkalemia, degradation of renal function, gynecomastia) were, however, significantly higher in the treated group compared to placebo.	7
Bapoje SR. et al. (2013); USA [52]	Systematic review & meta-analysis	Anti-aldosterone / Placebo; "usual care"	HF; <45%; I-IV	RCTs n= 8; n= 11,875	Range: 3 to 24	Mineralocorticoid receptor antagonists (or anti-aldosterones) reduced the risk of SCD in patients with left ventricular dysfunction.	8
Wei J., et al. (2010); China [20]	Meta-analysis	Anti-aldosterone / Placebo; "usual care"	HF; <45%; NA	RCTs n= 6 (two are not double blind), n= 00000	Range: 2 to 24	Two [Pitt et al, 1995 and Pitt et al 2003] of the six-included studies showed a significant reduction of SCD in the group of Spironolactone vs placebo and the group of Eplerenone vs. Placebo, cited respectively.	5
Solomon SD., et al. (2016); USA [21]	Meta-analysis	Sacubitril; valsartan / ACE-i	HF; <30%; II-IV	RCTs n= 3; n= 14742	Range 6 to 27	The authors concluded that combined neprilysin/RAS inhibition reduced all-cause mortality in HFrEF.	7
Flather MD et al., (2000); Canada [23]	Systematic review	ACE-i / Placebo	CHF; <45%; NA	RCTs n= 5, n= 12,763	Range: 15 to 42	This meta-analysis showed a lower risk of death in ACE-i treated group compared to placebo.	3
Garg R. et al., (1995); Canada [22]	Systematic review & meta-analysis	ACE-i / Placebo	CHF; <45% ; I-IV	RCTs n= 32, n= 7,105	Range: 3 to 42	Overall, this study reported a significant reduction of total mortality (attributed mainly to less progressive HF deaths) and hospitalization for worsening HF.	2
Rain & Rada, (2015) ; Chile [24]	Systematic review	ARB/ ACE-i	HF ; <45% - < 35% ; II-IV	RCTS = 8 ; n= 5201	NA	The authors concluded that ARBs are probably as effective in mortality reduction as ACE-I with probably less withdrawal rate due to adverse effects.	NA*
Heran et al., (2012) ; Canada [25]	Systematic review & meta-analysis (Cochrane)	ARB (or ARB + ACE-i) / Placebo ; ACE-i	HF ; ≤40% ; II-IV	RCTS n= 24 ; n= 25,051	Range: 1 to 49.5	Compared to placebo or in addition to ACE-i, ARBs did not reduce all-cause mortality.	10
Shibata et al., (2008) ; Canada [26]	Systematic review & meta-analysis	ARB/ Placebo ; ACE-i	HF ; <40% ; I-IV	RCTs n= 7 ; n= 27,495	Range: 11 to 41	Compared to ACE-i or used in combination, ARBs provided no beneficial effects on mortality. A 17% reduction in hospitalizations was observed.	4

For peer review only - http://bmjopen.bmj.com/site/about/guidelines.xhtml

Lee et al., (2004) ; USA [27]	Meta-analysis	ARB/ Placebo ; ACE-i	CHF , AMI ; ≤45% ; II–IV	RCTs n= 24 ; n= 38,080	Range: 1 to 41	Compared to ACE-I, ARBs do not differ in efficacy for reducing all-cause mortality in CHF and AMI patients.	7
Dimopoulos et al., (2004) ; UK [28]	Meta-analysis	ARB/ Placebo ; ACE-i	CHF; <40% ; II–IV	RCTs n= 4; n = 7666	Mean: 31	ARBs can be used to prevent events in ACE-i-treated heart failure patients who are not suitable for beta-blockers.	3
Jong P., et al. (2002), Canada [29]	Systematic review & meta-analysis	ARB (or ARB + ACE-i) / Placebo ; ACE-i	HF; ≤35% - ≤45%; II–IV	RCTs n= 17; n = 12,469	Range: 1 to 23	The authors could not conclude any superiority of ARBs versus controls, stating this might be due to the use of ACE-i as a comparator or background treatment in the majority of included trials.	8
Rain & Rada, (2017); Chile [30]	systematic review	Statins / Placebo ; "usual care"	HF; <45%; I-IV	RCTs n= 25; n = NR	NA	The authors summarized that statins do not decrease mortality in chronic heart failure and might lead to a small reduction in hospital admissions for HF.	NA*
Al-Gobari M., et al. (2017), Switzerland [5]	Systematic review & meta-analysis	Statins / Placebo ; "usual care"	HF, ischaemic/nonischaemic; NA; I-IV NA	RCTs n= 24; n = 11,463	Range: 1 to 46.8	Statins do not significantly reduced SCD and all-cause mortality. They may or may not reduce hospitalizations due to worsening heart failure (HWHF)	7
Bonsu KO, et al. (2015), Malaysia [31]	Meta-analysis	Statins / Placebo ; "usual care"	HF; <45%; I-IV	RCTs n= 13; n = 10,966	Range: 3 to 46.8	Lipophilic statins showed significant decrease in all-cause mortality, cardiovascular mortality and hospitalization for worsening heart failure.	8
Wang JQ., et al. (2014); China [32]	Meta-analysis	Statins / Placebo ; "usual care"	HF; NA; NA	RCTs n= 6 (9: observational studies), n= 10,016	Range: 12 to 46.8	The authors concluded that statins reduce sudden cardiac death and all-cause mortality in HF.	5
Liu G. et al. (2014); China [33]	Meta-analysis	Statins / Placebo ; "usual care"	HF; <45%; I-IV	RCTs n=13 ; n = 1,532	Range: 3 to 35.5	The authors reported significant decrease in all-cause mortality but recommended cautious interpretation and further research.	7
Rahimi K. et al. (2012); UK [38]	Meta-analysis	Statins / Placebo ; "usual care"	HF, MI, Primary prevention, diabetes, ACS, CHD; NA; NA	RCTs n= 37 n= 155,020		Statins have a modest effect on SCD but no substantial protective effect on ventricular arrhythmic events.	6
Zhang S. et al. (2011); China [34]	Meta-analysis	Statins / Placebo ; "usual care"	HF; <45%; I-IV	RCTs n=13 ; n = 10,447	Range: 2 to 46.8	This meta-analysis concluded of no difference between treatment groups but benefits may occur in some specific populations and with a specific statin.	7
Xu M. et al. (2010); China [35]	Meta-analysis	Statins / Placebo ; "usual care"	HF; <45%; I-IV	RCTs n= 7, n= 540	Range: 3 to 31	The authors suggested that atorvastatin treatment is effective and reduce all-cause mortality and hospitalization for worsening HF.	6
Lipinski MJ. (2009); USA [36]	Meta-analysis	Statins / Placebo ; "usual care"	HF; <45%; I-IV	RCTs n= 10; n= 10,192	Range 3 to 47	The authors stated that statins are safe and improve LVEF and decrease hospitalization for worsening HF.	7
Levantesi G. et al. (2007); Italy [37]	Meta-analysis	Statins / Placebo ; "usual care"	Secondary prevention; NA; NA	RCTs n= 10 n= 22,275	Range: 6 to 73.2	Statins were associated with a significant risk reduction for SCD (in secondary prevention settings).	3
Claro JC., et al. (2015), Chile [39]	Systematic review & meta-analysis (Cochrane)	Amiodarone / Placebo; "usual care"	Sub-analysis : heart failure ; NA; NA	RCTs n= 11, n= 5,006	NA	In HF sub-population, amiodarone showed a statistically significant reduction for SCD but not for all-cause mortality. Authors judged the quality of the evidence for the whole population (primary prevention) as low to moderate and for secondary prevention population as very low.	10
Santangeli P. et al. (2012); USA [40]	systematic review	Amiodarone / Placebo	cardiovascular disease; NA; NA	NA	NA	Amiodarone has less favorable net clinical benefits for prophylaxis of SCD because of adverse effects.	5
Piccini JP., et al. (2009); USA [41]	Meta-analysis	Amiodarone / Placebo ; "usual care"	HF, AMI; <45%; II-IV	RCTs n= 15, n= 8,522	Range: 2 to 12	In HF sub-population, amiodarone showed a statistically significant reduction for SCD but not all-cause mortality.	7
ATMA Investigators (1997) [42]	Meta-analysis	Amiodarone / Placebo ; "usual care"	Post-MI and CHF; 31%; NA	NA	Range: 4.8 to 25.8	Amiodarone reduced arrhythmic/sudden death in high-risk patients with recent MI or CHF. All-cause mortality decreased by 13 %.	3
Sim I. et al., (1997); USA [43]	Meta-analysis	Amiodarone / Placebo ; "usual care"	Subgroup : heart failure; <45%; NA	RCTs n=5, n= 4,125	Range: 6 to 45.6	Amiodarone reduced all-cause mortality in high SCD risk groups.	5
Das MK. et al., (2010): USA [44]	Narrative Review	Anti-arrhythmics / Placebo ; "usual care"	Subgroup : heart failure; NA; NA	NA	NA	Class I Anti-arrhythmic drugs (AADs) increased all-cause mortality and SCD in Post-MI patients. Amiodarone (class III AADs) decreased or have neutral effect on SCD. Caution is warranted to outweigh risks of proarrhythmia and	NA*

For peer review only - http://bmjopen.bmj.com/site/about/guidelines.xhtml

						other adverse effects.	
Hilleman DE. et al., (2001); USA [45]	Narrative Review	Anti-arrhythmics / Placebo ; "usual care"	HF; <45%; NA	RCTs n= 6 n= 10440	Range: 6.5 to 45	Beta-blockers (Bisoprolol, Carvedilol, Metoprolol) reduced total mortality and SCD in HF. Class I antiarryhthmics increased mortality and SCD in a post hoc analysis of SPAF-I study. Amiodarone had mixed results and dofetilide did not reduce mortality or SCD.	NA*
Rizos EC., et al. (2012); Greece [46]	systematic review & meta-analysis	Omega 3 Fatty acids / Placebo ; "usual care"	cardiovascular diseases; NA; NA	RCTs n= 20; n= 68,680	NA	Omega-3 PUFAs supplementation were not associated with a lower risk of all-cause mortality or sudden cardiac death	8
Kotwal S. et al. (2012); Australia [47]	systematic review & meta-analysis	Omega 3 Fatty acids / Placebo ; "usual care"	cardiovascular diseases, heart failure admissions; NA; NA	RCTs n= 20; n= 62,851	Range: 6 to 72	The authors concluded that there is no clear effect on total mortality and sudden death outcomes.	7
Kwak SM., et al. (2012); Korea [48]	Meta-analysis	Omega 3 Fatty acids / Placebo ; "usual care"	secondary prevention of cardiovascular disease; NA; NA	RCTs n=14; n= 20,485	NA	This meta-analysis concluded of insufficient evidence.	8
Chen Q., et al. (2011); China [49]	Meta-analysis	Omega 3 Fatty acids / Placebo ; "usual care"	cardiovascular disease; NA; NA	RCTs n= 10; n= 33,429	NA	Omega-3 fatty acids did not appear to reduce SCD under guideline-adjusted treatment for CVD secondary prevention.	7
Marik PE., et al. (2009); USA [50]	systematic review	Omega 3 dietary supplements / Placebo; olive oil ; corn oil, sunflower oil ; "usual care"	cardiovascular disease; NA; NA	RCTs n= 11; n= 39,044	Range: 12 to 55.2	Dietary supplementation with omega-3 fatty acids reduced SCD and all-cause mortality	4
Wang C., et al. (2006); USA [51]	systematic review	n-3 Fatty acids / Placebo/olive oil ; corn oil, sunflower oil ; "usual care"	Primary and secondary prevention; NA; NA	RCTs n= 12; n= 32,981	Range: 12 to 48	The authors concluded of a significant reduction in all-cause mortality and SCD with n-3 Fatty acids from fish or fish-oil supplements but not α-linolenic acid.	6

Risk of bias and quality of reviews

As shown in **Table 1**, the AMSTAR scores for quality assessment of included reviews widely ranged from 2 to 10 (out of 11). All reviews had one score less because of non-listing of excluded primary studies except Cochrane reviews [25 39] which scored 10 because of non-inclusion of grey literature in the search strategy in one review and missing information for funding resources of included primary studies in another, cited respectively (see **S2 Table**). We did not assess the AMSTAR score for four studies of which two [44 45] were narrative reviews and the other two [24 30] were overviews of reviews.

The risk of bias of the included primary studies within reviews remained as judged by the original reviews' authors with the exception of the newly added randomized trials in the update of the angiotensin receptor blockers review that we assessed (GRADE) (**Table 2**). The quality of evidence for beta-blockers and anti-aldosterone agents obtained a high quality on the GRADE scale, while angiotensin-converting enzyme inhibitors (ACE-i), amiodarone and statins obtained a moderate quality. However, combined angiotensin receptor blockers/neprilysin inhibitors had a moderate and high quality for SCD and all-cause mortality outcomes respectively, whereas angiotensin receptor blockers had a low quality of evidence (**Table 2**).

For peer review only - http://bmjopen.bmj.com/site/about/guidelines.xhtml

Table 2: Summary of findings and Grade evaluation for sudden cardiac death and all-cause mortality

Drug interventions for SCD and all-cause mortality prevention in heart failure patients							
Outcome	Intervention / comparison	Assumed risk with comparator	Corresponding risk with intervention	Relative effect (95% CI)	Number of participants (No of studies)	Quality of the evidence (GRADE)	Comments
**Sudden cardiac death**							
	Beta-blockers / placebo	77 per 1000	54 per 1000 (49 to 60)	OR 0.69 (0.62 to 0.77)	24,779 (26 RCTs)	⊕⊕⊕⊕ high*	I^2=0% (P=0.57)
	Anti-aldosterone inhibitor / Placebo ; “usual care”	61 per 1000	49 per 1000 (41 to 60)	RR 0.81 (0.67 to 0.98)	8,301 (5 RCTs)	⊕⊕⊕⊕ high*	I^2=8% (P=0.36)
	ARB;Neprilysin inhibitor/ ACE-i	74 per 1000	60 per 1000 (51 to 70)	RR 0.81 (0.69 to 0.95)	8,399 (1 RCT)	⊕⊕⊕⊖ moderate[e]	
	ACE-i / Placebo	59 per 1000	54 per 1000 (43 to 65)	OR 0.91 (0.73 to 1.11)	6,988 (30 RCT)	⊕⊕⊕⊖ moderate[b]	I^2=0% (P=0.94)
	ARB (or ARB + ACE-i) / Placebo ; ACE-i	93 per 1000	94 per 1000 (76 to 117)	RR 1.01 (0.81 to 1.25)	13,884 (5 RCTs)	⊕⊕⊖⊖ Low[b,c]	I^2=71% (P=0.007)
	Statins / placebo ; “usual care”	108 per 1000	100 per 1000 (76 to 131) [99 per 1000 (72 to 131)]	RR 0.92 (0.7 to 1.21) [OR 0.90 (0.64 to 1.24)]	10,077 (8 RCTs)	⊕⊕⊕⊖ moderate[a]	I^2=42.6% (P=0.094
	Amiodarone / Placebo; “usual care”	118 per 1000	93 per 1000 (79 to 110)	RR 0.79 (0.67 to 0.93)	5,006 (11 RCTs)	⊕⊕⊖⊖ Low[a,b]	
	Omega 3 Fatty acids / Placebo ; “usual care”	93 per 1000	88 per 1000 (77 to 102)	RR 0.94 (0.82 to 1.09)	6,975 (1 RCT)	⊕⊕⊕⊖ moderate[e]	
**All-cause mortality**							
	Beta-blockers / placebo	178 per 1000	127 per 1000 (113 to 141)	OR 0.67 (0.59 to 0.76)	24,779 (26 RCTs)	⊕⊕⊕⊕ high*	I^2= 40% (P=0.02)
	Anti-aldosterone inhibitor / Placebo ; “usual care”	200 per 1000	162 per 1000 (148 to 176)	RR 0.81 (0.74 to 0.88)	9,019 (10 RCTs)	⊕⊕⊕⊕ high*	I^2=0% (P=0.56)
	ARB;Neprilysin inhibitor/ ACE-i	183 per 1000	158 per 1000 (145 to 172)	RR 0.86 (0.79 to 0.94)	14,742 (3 RCTs)	⊕⊕⊕⊖ moderate[b]	I^2=0% (P=0.42)
	ACE-i / Placebo	219 per 1000	178 per 1000 (158 to 198)	OR 0.77 (0.67 to 0.88)	7,105 (32 RCT)	⊕⊕⊕⊖ moderate[b]	I^2=0% (P=0.95)

For peer review only - http://bmjopen.bmj.com/site/about/guidelines.xhtml

	ARB (or ARB + ACE-i) / Placebo ; ACE-i	183 per 1000	177 per 1000 (161 to 197)	RR 0.97 (0.88 to 1.08)	19,510 (27 RCTs)	⊕⊕⊖⊖ Low[b,d]	I^2=24% (P=0.14)
	Statins / placebo ; "usual care"	273 per 1000	240 per 1000 (205 to 278) [233 per 1000 (199 to 273)]	RR 0.88 (0.75 to 1.02) OR 0.81 (0.66 to 1)	11,024 (13 RCTs)	⊕⊕⊕⊖ moderate[a]	I^2=37.7% (P=0.083)
	Amiodarone / Placebo ; "usual care"	264 per 1000	237 per 1000 (211 to 266)	RR 0.90 (0.80 to 1.01)	5,006 (11 RCTs)	⊕⊕⊖⊖ Low[a,b]	
	Omega 3 Fatty acids / Placebo ; "usual care"	291 per 1000	274 per 1000 (253 to 294)	RR 0.94 (0.87 to 1.01)	6,975 (1 RCT)	⊕⊕⊕⊖ moderate[e]	

[a] Likelihood of Publication bias presence with an asymmetric funnel plot.

[b] The studies reported to generally have a moderate - high risk of bias due to allocation concealment and blinding reporting.

[c] Inconsistent results ranged from no effect to insignificant increase of events (I^2 = 71%)

[d] Most studies have small sample and wide confidence intervals including no effect with appreciable harm or benefit.

[e] Estimation comes from one single clinical trial. No data obtained from other relevant studies for this outcome

* Although graded high, this might be downgraded into moderate if we strictly consider the risk of bias of primary studies other than an overall estimation.

For peer review only - http://bmjopen.bmj.com/site/about/guidelines.xhtml

For peer review only

Up-to-dateness of included reviews

Most retrieved evidence was published within the last 10 years (2008 and on) and 7 (out of 9) drug interventions with updated systematic reviews were within the last 5 years (2012 and on). Moreover, we updated the pooled results for ARBs, which resulted in slightly different results compared to the original Cochrane review [25].

Effectiveness of interventions

We report below the summaries of the effectiveness of the drug interventions considered that we have categorized into effective, ineffective, and uncertain effectiveness (inconclusive or conflicting evidence).

Effective interventions

Beta-blockers

Meta-analyses and systematic reviews of randomized controlled trials [13-18] provided overwhelming evidence that beta-blockers (BBs) decrease the risk of SCD and all-cause mortality in HFrEF patients. The quality of the evidence was rated high with a relative effect of 0.69 for SCD [odds ratio (OR), 95% confidence interval (CI) 0.62 to 0.77], and of 0.67 for all-cause mortality [OR, 95% CI (0.59 to 0.76)] (**Table 2**).

Anti-aldosterone agents

Published studies about mineralocorticoid-receptor antagonists (antimineralocorticoids) or (so-called) anti-aldosterones appeared effective in SCD and all-cause mortality prevention [19 20 52]. However, in a recent systematic review [19], adverse effects (hyperkalemia, degradation of renal function, gynecomastia) were significantly higher in the anti-aldosterone-treated group compared to placebo. The quality of the evidence was rated high with relative effect for SCD [risk ratio (RR) 0.81, 95% confidence interval (CI) 0.67 to 0.98], and all-cause mortality [RR 0.81, 95% CI (0.74 to 0.88)] (**Table 2**).

Angiotensin receptor blocker/neprilysin inhibitor

One meta-analysis [21] estimated the effects of combined neprilysin renin-aldosterone system inhibition and reported a reduction in SCD and all-cause mortality. The finding was principally derived from one RCT (PARADIGM-HF) [53] that showed about 16% reduction of all-cause mortality in favor of sacubitril/valsartan (LCZ696 previously) compared to enalapril (an ACE-i). This mortality reduction was attributed to a decline on both SCD (20%) and pump failure deaths [4]. **Table 2** shows the relative effect for SCD [risk ratio (RR) 0.81, 95% confidence interval (CI) 0.69 to 0.95], and all-cause mortality [RR 0.86, 95% CI (0.79 to 0.94)]. The moderate quality of the evidence for SCD outcome was due to estimation from one single clinical trial and absence of data from other included studies. All-cause mortality was, however, rated as high with a possibility of downgrading in case of a detectable publication bias or inexistent class effect.

For peer review only - http://bmjopen.bmj.com/site/about/guidelines.xhtml

For peer review only

Ineffective Interventions

Angiotensin-converting enzyme inhibitors (ACE-i)

Although two systematic reviews [22 23], with an AMSTAR score of 2/11 and 3/11 respectively, reported a decline in total mortality and less progressive HF deaths, SCD events did not significantly decrease [Odds ratio (OR) 0.91, 95% confidence interval (CI) 0.73 to 1.12]. The quality of the evidence was rated as moderate because of unclear or high risk of bias in included primary studies (**Table 2**).

Angiotensin receptor blockers (ARBs)

As shown in **Fig 2 and Fig 3**, we updated a Cochrane review [25] by including more eligible primary studies such as SUPPORT trial [54] comparing ARBs to controls which resulted in slightly different effect size for SCD [Risk ratio (RR) 1.01, 95% confidence interval (CI) 0.81 to 1.25], and all-cause mortality [RR 0.97, 95% CI (0.88 to 1.08)]. In stratified analyses, ARBs compared to placebo remained ineffective for all-cause mortality [RR 0.79, 95% CI (0.55 to 1.13)]. Similarly, ARBs, compared to ACE-I or in combination versus ACE-I alone, were not superior in all-cause mortality reduction [RR 0.87, 95% CI (0.56 to 1.36), [RR 0.99, 95% CI (0.90 to 1.09)] respectively] (**Fig 2**). The quality of the evidence is rated as low because of risk of bias, imprecision and inconsistency (I^2 = 71, P=0.007 for SCD outcome) (**Table 2**). In addition, the funnel plot for all-cause mortality outcome showed no evidence of publication bias (**Fig 4**). No estimation of publication and no funnel plot was drawn for SCD as only five studies reported this outcome.

Statins

Published systematic reviews and meta-analyses about statins in HF were inconsistent [5 30-38] with a recent tendency toward inefficacy in total mortality and SCD prevention. The quality of the evidence was rated as moderate because of a likelihood of publication bias revealed on the most up-to-date systematic review [5] (**Table 2**).

Unclear evidence of effectiveness

The evidence of effectiveness of the drug interventions reported below was considered uncertain due either to conflicting or inconclusive evidence.

Amiodarone & anti-arrhythmic drugs (AADs)

Recently published systematic reviews[39 41] for amiodarone showed a significant reduction for SCD but not for all-cause mortality with less favorable net clinical benefits[40]. Other older reviews [42 43] of minor quality (AMSTAR of 3/11 and 5/11 cited respectively) reported a decline of both SCD and all-cause mortality. The quality of evidence for amiodarone was rated as low because of unclear or high risk of bias and potential publication bias in primary studies (**Table 2**). No systematic review for anti-arrythmic drugs (AADs) of other

For peer review only - http://bmjopen.bmj.com/site/about/guidelines.xhtml

classes or drugs (other than amiodarone) were retrieved and hence two narrative reviews [44 45] reported that class I anti-arrhythmics increased SCD and all-cause mortality. These narrative reviews called for caution regarding the mixed results of amiodarone and its adverse effects.

Omega-3 polyunsaturated fatty acids (PUFA) and fish oil supplementation

No systematic review was exclusively conducted in heart failure patients for this intervention. One primary study[55], known as GISSI-Prevenzione HF, recruited patients with chronic heart failure and reported a lower mortality events in the n-3 polyunsaturated fatty acids (PUFA) group compared to placebo group. The authors reported an adjusted hazard ratio of 0·91 [95·5% CI 0·833–0·998], p=0·041). However, relative risk in our analysis remained statistically insignificant [RR 0.94, 95% CI (0.87 to 1.01), p=0.10] and [RR 0.94, 95% CI (0.81 to 1.09), p=0.42] for all-cause mortality and SCD, respectively. Our assessment for the quality of the evidence involving GISSI-Prevenzione HF was moderate because of an absence of data of any other relevant studies (**Table 2**). In addition, some recent systematic reviews [46-49] included patients regardless of their cardiovascular disease and concluded of no clear effect, insufficient evidence or no reduction on SCD and all-cause mortality outcomes. Meanwhile, some older studies [50 51] reported that omega-3 fatty acids and fish-oil supplements (other than α-linolenic acid [51]) reduced SCD and all-cause mortality.

Discussion

Our assessment of the effectiveness of drug interventions to prevent SCD or all-cause mortality in HFrEF patients indicated that beta-blockers (BBs), antialdosterone agents, as well as combined angiotensin receptor blocker (ARB)/neprilysin inhibitors were effective.

Previously reported meta-analyses and systematic reviews of randomized clinical trials [13-18] indicated that beta-blockers are effective in the prevention of SCD and all-cause mortality in HFrEF. However, although they were increasingly used as a usual “routine” care in the compared arms of the more recently published clinical trials[56], beta-blockers stayed underused for long time and may still be. Mineralocorticoid receptor antagonists or antialdosterone drugs have been reported effective in HFrEF and reduce SCD and all-cause mortality [19 20 52 57]. Our summary of finding and consistency of results support this claim with a high quality of evidence. Only one retrieved meta-analysis [21] supported the effectiveness of combined angiotensin receptor blocker (ARB)/neprilysin inhibitor. Although the authors acknowledged the limitation of their meta-analysis, which was not based on a systematic review but merely the meta-analysis of three well-known trials published in high impact journals (i.e IMPRESS [58], OVERTURE [59] and Paradigm-HF [53]). The quality of the evidence is, however,

For peer review only - http://bmjopen.bmj.com/site/about/guidelines.xhtml

moderate for SCD and high for all-cause mortality, albeit our inability to assess any presence of a class effect or a potential publication bias.

We found that ACE-i showed a total mortality reduction in clinical trials and systematic reviews of heart failure patients [22 23]. However, our overview showed that ACE-i, surprisingly, did not significantly decrease SCD with a moderate quality of evidence.

In addition, we found that neither ARBs nor statins reduced SCD and/or all-cause mortality. Our findings for ARBs were in agreement with Jong and colleagues [29] and Shibata et al. [26] or Dimopoulos et al. [28] but in contradiction to Lee VC et al. [27] and Rain & Rada's conclusion [24]. Our up-to-date meta-analysis for ARBs included only five primary studies but large-scale trials that reported SCD events and the pooled estimate showed inefficacy of ARBs compared to control in SCD prevention [RR 1.01, 95% CI (0.81 to 1.25). Moreover, the addition of current trials such as SUPPORT [54], improved the statistical power of detecting an effect if existed and the summary statistic moved from a borderline effect to an insignificant result. (**Fig 2**). Of note, Jong and colleagues [29] attributed this inefficacy of ARBs in HF to the background treatment by ACE-i.

Within the current evidence, ARBs should not be seen as interchangeable to ACE-I, which also showed neutral effect on SCD, without a proper reason. Therefore, in a high-risk SCD patient, another therapeutic strategy should be adopted and an ARB/neprilysin inhibitor might be an alternative. Actually, this approach has been proposed by the 2016 European Society of Cardiology (ESC) guidelines[3].

The addition of statins to the therapy regimen of heart failure patients had no survival benefits. Actually, a recent systematic review and meta-analysis indicated that statins did not reduce SCD nor all-cause mortality [5]. Our current study reached the same conclusion with similar quality of evidence.

Our overview showed unclear evidence of effectiveness of Omega-3 polyunsaturated fatty acids (PUFA), fish oil supplementation and anti-arrhythmic drugs (AADs). The latter intervention had an evidence originated from only narrative reviews, as we did not identify any systematic reviews. Also, only one n-3 PUFA clinical trial [55] conducted in heart failure patients and reported a statistically significant mortality reduction, but it was still not supported by other trials and recent systematic reviews [46-49] , a finding that justified our conclusion of unclear evidence. Moreover, no other data or no more systematic reviews conducted in HF were retrieved by our electronic and manual searches.

AADs are classified into four categories [44]: sodium channel blocking drugs (class I), beta-blockers (class II), potassium channel blockers (class III), and calcium channel blockers (class IV). We found inconclusive evidence of effectiveness of all categories, with the exception of beta-blockers. The evidence of effectiveness of

For peer review only - http://bmjopen.bmj.com/site/about/guidelines.xhtml

class I, III and IV is inconclusive, neutral or even detrimental to patients as for class I AADs [44 45]. Amiodarones, which present class I, II, III, and IV effects, reported mixed results with potential SCD prevention but with adverse effects [41] and potentially, but rare [60], life-threatening proarrhythmias [44].

Our overview has some limitations. First, we limited the scope of our study to drug treatment, thus excluding devices like implantable cardioverter defibrillators. Second, we may have failed to include other drug interventions used in HFrEF. However, our overview included most evidence-based pharmacological therapy in heart failure as pre-specified in our published protocol [10]. Third, we did not use specific drug names in our literature search strategy, in order to avoid omitting a therapy that evaluated SCD and/or all-cause mortality prevention in heart failure patients. Fourth, we based our analyses on existing systematic reviews and meta-analyses and we updated only one meta-analysis. Furthermore, as indicated by the AMSTAR score, the methodological quality of some of the existing reviews was suboptimal. Fifth, we did not assess the safety of the evaluated drug interventions, nor the contra-indications to their prescription, drug-drug interactions, as well as treatment adherence. Indeed, we considered that these important aspects were out of the scope of our analysis. Sixth, we were unable to do a sensitivity analysis, initially suggested in our protocol, for ischaemic versus non-ischaemic heart failure due to limited data availability. Finally, a potential source of bias relates to authors of this overview being authors of three of the included reviews. However, the adopted methodology is in line with systematic reviews guidelines and ensured a double check of data and methodological evaluation by at least two reviewers and a published protocol [10].

It is noteworthy that high quality evidence does not necessarily imply strong recommendations, and strong recommendations can arise from low quality evidence [61]. Therefore, when one intervention is graded high, it is not our intention to say that it is highly recommended, as we did not assess the level of recommendation in our study. In fact, level of recommendation not only depends on the strength of evidence but also (among others) on values and preferences of patients, net benefits and cost-effectiveness of a particular intervention.

A major strength of our study is that it summarizes and synthesizes the effectiveness of most evidence-based drug interventions in heart failure patients with reduced ejection fraction for SCD prevention and classified drug interventions according to the current evidence of their effectiveness. This categorization could help health professionals and patients making evidence-based decisions based on updated knowledge, particularly whenever a high-risk SCD patient is identified. Consequently, there might be a shift towards a therapeutic strategy based on SCD prevention-effective therapy.

For peer review only

For peer review only - http://bmjopen.bmj.com/site/about/guidelines.xhtml

In conclusion, our overview indicates that only three drug interventions (beta-blockers, anti-aldosterones, combined angiotensin receptor blockers/neprilysin inhibitors) significantly reduce SCD and improve overall survival among individuals with heart failure and reduced ejection fraction. On the other hand, there is no evidence of efficacy of ARBs to reduce neither all-cause mortality nor SCD, and ACE-i do not significantly reduce SCD events. When the goal of drug therapy is to reduce SCD, especially in high-risk patients, our synthesis supports the option of replacing ACE-i by combined angiotensin receptor blockers/neprilysin inhibitors. This option has already been proposed by the 2016 ESC guidelines.

Summary for current knowledge

- It is known that mortality of heart failure is very high and that new therapy and strategies are needed to better control disease progression and to prevent the high mortality event rates, in particular sudden cardiac deaths (SCD).
- There is uncertainty about the actual effectiveness of several drugs used in heart failure patients to reduce SCD and all-cause mortality events.
- A recent clinical trial (Paradigm-HF), which compared combined angiotensin receptor blocker/neprilysin inhibitors to enalapril (an angiotensin converting enzyme inhibitor), reported a 20% reduction in SCD and 16% in all-cause mortality after 27 months of follow-up.

Summary for this study outcome

- Our overview shows that beta-blockers, anti-aldosterones, as well as combined angiotensin receptor blockers/neprilysin inhibitors significantly reduced sudden cardiac death (SCD) and all-cause mortality events; while angiotensin receptor blockers did not.
- Angiotensin converting enzyme inhibitors reduced all-cause mortality but failed to significantly reduce SCD events.
- Our study calls for the use of the most effective regimen especially in high-risk SCD patients.

Author contribution

Design and conception: MA, FG, BB. (MA is the guarantor). Project administration: BB. Writing original draft: MA. Critical analysis: MA, SA, FG, BB. Data curation: MA, SA. Statistical analysis: MA. Proofread and approved the final draft: MA, SA, FG, and BB.

For peer review only - http://bmjopen.bmj.com/site/about/guidelines.xhtml

Competing interests

We declare that some authors of this overview are also authors of some of the included reviews. However, at least two reviewers systematically checked and validated the extracted data including study qualities. We declare no other competing interests exist.

Funding

MA, the corresponding author, received a financial grant (2014.0193) from The Federal Department of Economic Affairs, Education and Research (EAER), Switzerland, also from Département universitaire de médecine et santé communautaires (DUMSC), Lausanne university hospital (CHUV), Switzerland. The former funders had no role in study design or conception, study analysis, writing or preparation of the manuscript.

Ethics committee approval

This study did not need to ask for ethical approval and no direct patient involvement occurred.

Data sharing

All data are available in the manuscript and its supported files. Any more information can be requested from the corresponding author.

Acknowledgements

We thank Emilie Antille for her technical assistance.

Supporting materials

- S1 File: PRISMA checklist
- S2 File: Search strategy equations and result
- S3 Table: AMSTAR scores.
- S4 Table: Funding data of included reviews

Abbreviations

- ACE-i: angiotensin-converting enzyme inhibitor
- ACS: acute coronary syndrome
- ARBs: angiotensin receptor blockers
- ATMA: amiodarone trial meta-analysis
- BBs: beta-blockers
- CHD: coronary heart disease
- CHF: chronic heart disease

For peer review only - http://bmjopen.bmj.com/site/about/guidelines.xhtml

- Critical analysis of SRs about effectiveness of statins in HF patients is previously published elsewhere
- GRADE: Grading of Recommendations, Assessment, Development and Evaluation
- HF: heart failure
- HFrEF: heart failure with reduced ejection fraction
- HFrEF: heart failure with reduced ejection fraction
- LVD: left ventricular dysfunction
- MI: Myocardial Infarction
- NA: not available or not applicable.
- NR: not reported
- NYHA: New York Heart Association class
- Post-MI: post myocardial infarction
- PUFAs: polyunsaturated fatty acids
- RAS: renin angiontensin system
- RCTs: randomized controlled trials
- SCD: sudden cardiac death
- SPAF-I: Stroke prevention Atrial Fibrillation Study

References

1. Thom T, Haase N, Rosamond W, et al. Heart disease and stroke statistics--2006 update: a report from the American Heart Association Statistics Committee and Stroke Statistics Subcommittee. *Circulation* 2006;113(6):e85-151. doi: 10.1161/circulationaha.105.171600 [published Online First: 2006/01/13]
2. Tomaselli GF, Zipes DP. What causes sudden death in heart failure? *Circulation research* 2004;95(8):754-63. doi: 10.1161/01.RES.0000145047.14691.db [published Online First: 2004/10/16]
3. Ponikowski P, Voors AA, Anker SD, et al. 2016 ESC Guidelines for the diagnosis and treatment of acute and chronic heart failure: The Task Force for the diagnosis and treatment of acute and chronic heart failure of the European Society of Cardiology (ESC)Developed with the special contribution of the Heart Failure Association (HFA) of the ESC. *European heart journal* 2016;37(27):2129-200. doi: 10.1093/eurheartj/ehw128 [published Online First: 2016/05/22]
4. Desai AS, McMurray JJ, Packer M, et al. Effect of the angiotensin-receptor-neprilysin inhibitor LCZ696 compared with enalapril on mode of death in heart failure patients. *European heart journal* 2015;36(30):1990-7. doi: 10.1093/eurheartj/ehv186 [published Online First: 2015/05/30]
5. Al-Gobari M, Le HH, Fall M, et al. No benefits of statins for sudden cardiac death prevention in patients with heart failure and reduced ejection fraction: A meta-analysis of randomized controlled trials. *Plos One* 2017;12(2)
6. Lee E, Dobbins M, Decorby K, et al. An optimal search filter for retrieving systematic reviews and meta-analyses. *BMC medical research methodology* 2012;12:51. doi: 10.1186/1471-2288-12-51 [published Online First: 2012/04/20]
7. Wilczynski NL, Haynes RB. EMBASE search strategies achieved high sensitivity and specificity for retrieving methodologically sound systematic reviews. *Journal of clinical epidemiology* 2007;60(1):29-33. doi: 10.1016/j.jclinepi.2006.04.001 [published Online First: 2006/12/13]
8. Ouzzani M, Hammady H, Fedorowicz Z, et al. Rayyan-a web and mobile app for systematic reviews. *Systematic reviews* 2016;5(1):210. doi: 10.1186/s13643-016-0384-4 [published Online First: 2016/12/07]
9. Shea BJ, Grimshaw JM, Wells GA, et al. Development of AMSTAR: a measurement tool to assess the methodological quality of systematic reviews. *BMC medical research methodology* 2007;7:10. doi: 10.1186/1471-2288-7-10 [published Online First: 2007/02/17]
10. Al-Gobari M, Gueyffier F, Burnand B. Guideline-directed drug interventions for sudden cardiac death prevention in patients with heart failure and reduced ejection fraction: an overview of systematic reviews. *PROSPERO 2017:CRD42017067442* 2017; Available from http://www.crd.york.ac.uk/PROSPERO/display_record.asp?ID=CRD42017067442

For peer review only - http://bmjopen.bmj.com/site/about/guidelines.xhtml

11. Kunz R, Burnand B, Schünemann HJ. Das GRADE-System. *Der Internist* 2008;49(6):673-80. doi: 10.1007/s00108-008-2141-9
12. Mantel N, Haenszel W. Statistical aspects of the analysis of data from retrospective studies of disease. *Journal of the National Cancer Institute* 1959;22(4):719-48. [published Online First: 1959/04/01]
13. Chatterjee S, Biondi-Zoccai G, Abbate A, et al. Benefits of beta blockers in patients with heart failure and reduced ejection fraction: network meta-analysis. *BMJ (Clinical research ed)* 2013;346:no pagination.
14. Al-Gobari M, El Khatib C, Pillon F, et al. Beta-blockers for the prevention of sudden cardiac death in heart failure patients: a meta-analysis of randomized controlled trials. *Bmc Cardiovascular Disorders* 2013;13
15. Brophy JM, Joseph L, Rouleau JL. Beta-blockers in congestive heart failure. A Bayesian meta-analysis. *Annals of internal medicine* 2001;134(7):550-60. [published Online First: 2001/04/03]
16. Lee S, Spencer A. Beta-blockers to reduce mortality in patients with systolic dysfunction: A meta-analysis. *Journal of Family Practice* 2001;50(6):499-504.
17. Bonet S, Agusti A, Arnau JM, et al. Beta-adrenergic blocking agents in heart failure: benefits of vasodilating and nonvasodilating agents according to patients' characteristics: a meta-analysis of clinical trials (Structured abstract). *Archives of Internal Medicine* 2000;160(5):621-27.
18. Heidenreich PA, Lee TT, Massie BM. Effect of beta-blockade on mortality in patients with heart failure: A meta-analysis of randomized clinical trials. *Journal of the American College of Cardiology* 1997;30(1):27-34.
19. Le HH, El-Khatib C, Mombled M, et al. Impact of Aldosterone Antagonists on Sudden Cardiac Death Prevention in Heart Failure and Post-Myocardial Infarction Patients: A Systematic Review and Meta-Analysis of Randomized Controlled Trials. *Plos One* 2016;11(2)
20. Wei J, Ni J, Huang D, et al. The effect of aldosterone antagonists for ventricular arrhythmia: a meta-analysis. *Clinical cardiology* 2010;33(9):572-7.
21. Solomon SD, Claggett B, McMurray JJ, et al. Combined neprilysin and renin-angiotensin system inhibition in heart failure with reduced ejection fraction: a meta-analysis. *European journal of heart failure* 2016;18(10):1238-43. doi: 10.1002/ejhf.603 [published Online First: 2016/07/02]
22. Garg R, Yusuf S. Overview of randomized trials of angiotensin-converting enzyme inhibitors on mortality and morbidity in patients with heart failure. Collaborative Group on ACE Inhibitor Trials. *Jama* 1995;273(18):1450-6. [published Online First: 1995/05/10]
23. Flather MD, Yusuf S, Kober L, et al. Long-term ACE-inhibitor therapy in patients with heart failure or left-ventricular dysfunction: a systematic overview of data from individual patients. ACE-Inhibitor Myocardial Infarction Collaborative Group. *Lancet* 2000;355(9215):1575-81. [published Online First: 2000/05/23]
24. Rain C, Rada G. Is there any difference between angiotensin converting enzyme inhibitors and angiotensin receptor blockers for heart failure? *Medwave* 2015;15 Suppl 1:e6177. doi: 10.5867/medwave.2015.06.6177 [published Online First: 2015/08/08]
25. Heran BS, Musini VM, Bassett K, et al. Angiotensin receptor blockers for heart failure. *The Cochrane database of systematic reviews* 2012(4):Cd003040. doi: 10.1002/14651858.CD003040.pub2 [published Online First: 2012/04/20]
26. Shibata MC, Tsuyuki RT, Wiebe N. The effects of angiotensin-receptor blockers on mortality and morbidity in heart failure: a systematic review. *International journal of clinical practice* 2008;62(9):1397-402. doi: 10.1111/j.1742-1241.2008.01806.x [published Online First: 2008/09/17]
27. Lee VC, Rhew DC, Dylan M, et al. Meta-analysis: angiotensin-receptor blockers in chronic heart failure and high-risk acute myocardial infarction. *Annals of internal medicine* 2004;141(9):693-704. [published Online First: 2004/11/03]
28. Dimopoulos K, Salukhe TV, Coats AJ, et al. Meta-analyses of mortality and morbidity effects of an angiotensin receptor blocker in patients with chronic heart failure already receiving an ACE inhibitor (alone or with a beta-blocker). *International journal of cardiology* 2004;93(2-3):105-11. doi: 10.1016/j.ijcard.2003.10.001 [published Online First: 2004/02/21]
29. Jong P, Demers C, McKelvie RS, et al. Angiotensin receptor blockers in heart failure: meta-analysis of randomized controlled trials. *J Am Coll Cardiol* 2002;39(3):463-70. [published Online First: 2002/02/02]
30. Rain C, Rada G. Are statins beneficial for chronic heart failure? First update. *Medwave* 2017;17(Suppl2):e6853. doi: 10.5867/medwave.2017.6853 [published Online First: 2017/04/30]

For peer review only - http://bmjopen.bmj.com/site/about/guidelines.xhtml

31. Osei Bonsu K, Reidpath DD, Kadirvelu A. Hydrophilic versus lipophilic statin therapy on outcomes in patients with heart failure: A systematic review and an adjusted indirect comparison meta-analysis of randomised trials. *European heart journal* 2015;36:546.
32. Wang JQ, Wu GR, Wang Z, et al. Long-term Clinical Outcomes of Statin Use for Chronic Heart Failure: A Meta-analysis of 15 Prospective Studies. *Heart Lung and Circulation* 2014;23(2):105-13.
33. Liu G, Zheng XX, Xu YL, et al. Effects of lipophilic statins for heart failure: a meta-analysis of 13 randomised controlled trials. *Heart, lung & circulation* 2014;23(10):970-7. doi: 10.1016/j.hlc.2014.05.005 [published Online First: 2014/06/24]
34. Zhang S, Zhang L, Sun A, et al. Efficacy of statin therapy in chronic systolic cardiac insufficiency: a meta-analysis. *European journal of internal medicine* 2011;22(5):478-84. doi: 10.1016/j.ejim.2011.06.003 [published Online First: 2011/09/20]
35. Xu M, Yuan G, Wei F. Effect of atorvastatin in patients with chronic heart failure - insights from randomized clinical trials. *Archives of medical science : AMS* 2010;6(6):866-73.
36. Lipinski MJ, Cauthen CA, Biondi-Zoccai GG, et al. Meta-analysis of randomized controlled trials of statins versus placebo in patients with heart failure. *The American journal of cardiology* 2009;104(12):1708-16. doi: 10.1016/j.amjcard.2009.07.055 [published Online First: 2009/12/08]
37. Levantesi G, Scarano M, Marfisi R, et al. Meta-analysis of effect of statin treatment on risk of sudden death. *The American journal of cardiology* 2007;100(11):1644-50. doi: 10.1016/j.amjcard.2007.07.015 [published Online First: 2007/11/27]
38. Rahimi K, Majoni W, Merhi A, et al. Effect of statins on ventricular tachyarrhythmia, cardiac arrest, and sudden cardiac death: A meta-analysis of published and unpublished evidence from randomized trials. *European heart journal* 2012;33(13):1571-81.
39. Claro JC, Candia R, Rada G, et al. Amiodarone versus other pharmacological interventions for prevention of sudden cardiac death. *Cochrane Database of Systematic Reviews* 2015;0(12)
40. Santangeli P, Di Biase L, Burkhardt JD, et al. Examining the safety of amiodarone. *Expert Opinion on Drug Safety* 2012;11(2):191-214.
41. Piccini JP, Berger JS, O'Connor CM. Amiodarone for the prevention of sudden cardiac death: a meta-analysis of randomized controlled trials. *European heart journal* 2009;30(10):1245-53.
42. Effect of prophylactic amiodarone on mortality after acute myocardial infarction and in congestive heart failure: meta-analysis of individual data from 6500 patients in randomised trials (Structured abstract). *Lancet* 1997;350(2):1417-24.
43. Sim I, McDonald KM, Lavori PW, et al. Quantitative overview of randomized trials of amiodarone to prevent sudden cardiac death. *Circulation* 1997;96(9):2823-29.
44. Das MK, Zipes DP. Antiarrhythmic and Nonantiarrhythmic Drugs for Sudden Cardiac Death Prevention. *Journal of Cardiovascular Pharmacology* 2010;55(5):438-49.
45. Hilleman DE, Bauman AL. Role of antiarrhythmic therapy in patients at risk for sudden cardiac death: an evidence-based review. *Pharmacotherapy* 2001;21(5):556-75. [published Online First: 2001/05/15]
46. Rizos E, Ntzani E, Bika E, et al. Association between omega-3 fatty acid supplementation and risk of major cardiovascular disease events: a systematic review and meta-analysis (Structured abstract). *Jama* 2012;308(10):1024-33.
47. Kotwal S, Jun M, Sullivan D, et al. Omega 3 Fatty acids and cardiovascular outcomes: systematic review and meta-analysis. *Circulation Cardiovascular quality and outcomes* 2012;5(6):808-18. doi: 10.1161/circoutcomes.112.966168 [published Online First: 2012/11/01]
48. Kwak SM, Myung SK, Lee YJ, et al. Efficacy of omega-3 fatty acid supplements (eicosapentaenoic acid and docosahexaenoic acid) in the secondary prevention of cardiovascular disease: A meta-analysis of randomized, double-blind, placebo-controlled trials. *Archives of Internal Medicine* 2012;172(9):686-94.
49. Chen Q, Cheng L, Xiao T, et al. Effects of omega-3 fatty acid for sudden cardiac death prevention in patients with cardiovascular disease: a contemporary meta-analysis of randomized, controlled trials (Structured abstract). *Cardiovascular Drugs and Therapy* 2011;25(3):259-65.
50. Marik PE, Varon J. Omega-3 dietary supplements and the risk of cardiovascular events: a systematic review. *Clinical cardiology* 2009;32(7):365-72.

For peer review only - http://bmjopen.bmj.com/site/about/guidelines.xhtml

51. Wang C, Harris WS, Chung M, et al. n-3 Fatty acids from fish or fish-oil supplements, but not alpha-linolenic acid, benefit cardiovascular disease outcomes in primary- and secondary-prevention studies: a systematic review. *The American journal of clinical nutrition* 2006;84(1):5-17. [published Online First: 2006/07/11]

52. Bapoje SR, Bahia A, Hokanson JE, et al. Effects of Mineralocorticoid Receptor Antagonists on the Risk of Sudden Cardiac Death in Patients With Left Ventricular Systolic Dysfunction A Meta-analysis of Randomized Controlled Trials. *Circulation-Heart Failure* 2013;6(2):166-+.

53. McMurray JJ, Packer M, Desai AS, et al. Angiotensin-neprilysin inhibition versus enalapril in heart failure. *The New England journal of medicine* 2014;371(11):993-1004. doi: 10.1056/NEJMoa1409077 [published Online First: 2014/09/02]

54. Sakata Y, Shiba N, Takahashi J, et al. Clinical impacts of additive use of olmesartan in hypertensive patients with chronic heart failure: the supplemental benefit of an angiotensin receptor blocker in hypertensive patients with stable heart failure using olmesartan (SUPPORT) trial. *European heart journal* 2015;36(15):915-23. doi: 10.1093/eurheartj/ehu504 [published Online First: 2015/02/02]

55. Tavazzi L, Maggioni AP, Marchioli R, et al. Effect of n-3 polyunsaturated fatty acids in patients with chronic heart failure (the GISSI-HF trial): a randomised, double-blind, placebo-controlled trial. *Lancet* 2008;372(9645):1223-30. doi: 10.1016/s0140-6736(08)61239-8 [published Online First: 2008/09/02]

56. Shen L, Jhund PS, Petrie MC, et al. Declining Risk of Sudden Death in Heart Failure. *The New England journal of medicine* 2017;377(1):41-51. doi: 10.1056/NEJMoa1609758 [published Online First: 2017/07/06]

57. Anand K, Mooss AN, Mohiuddin SM. Aldosterone inhibition reduces the risk of sudden cardiac death in patients with heart failure. *Journal of the Renin-Angiotensin-Aldosterone System* 2006;7(1):15-19.

58. Rouleau JL, Pfeffer MA, Stewart DJ, et al. Comparison of vasopeptidase inhibitor, omapatrilat, and lisinopril on exercise tolerance and morbidity in patients with heart failure: IMPRESS randomised trial. *Lancet* 2000;356(9230):615-20. [published Online First: 2000/09/01]

59. Packer M, Califf RM, Konstam MA, et al. Comparison of omapatrilat and enalapril in patients with chronic heart failure: the Omapatrilat Versus Enalapril Randomized Trial of Utility in Reducing Events (OVERTURE). *Circulation* 2002;106(8):920-6. [published Online First: 2002/08/21]

60. Doggrell SA. Amiodarone - Waxed and waned and waxed again. *Expert Opinion on Pharmacotherapy* 2001;2(11):1877-90.

61. Guyatt GH, Oxman AD, Vist GE, et al. GRADE: an emerging consensus on rating quality of evidence and strength of recommendations. *BMJ : British Medical Journal* 2008;336(7650):924-26. doi: 10.1136/bmj.39489.470347.AD

For peer review only

Legends of figures

Fig 1: flow chart for search result

Fig 2: Efficacy of angiotensin receptor blockers (ARBs) compared with placebo**,** angiotensin-converting enzyme inhibitor (ACE-i), or combined in heart failure and reduced ejection fraction (HFrEF) for the prevention of **all-cause mortality**.

Fig 3: Efficacy of angiotensin receptor blockers (ARBs) compared with control in heart failure and reduced ejection fraction (HFrEF) for the prevention of **sudden cardiac death (SCD).**

Fig 4: Funnel plot of SE (log odds ratio) by odds ratio to evaluate publication bias for the efficacy of angiotensin receptor blockers (ARBs) compared with control in heart failure and reduced ejection fraction (HFrEF) for the prevention of **all-cause mortality.**

For peer review only - http://bmjopen.bmj.com/site/about/guidelines.xhtml

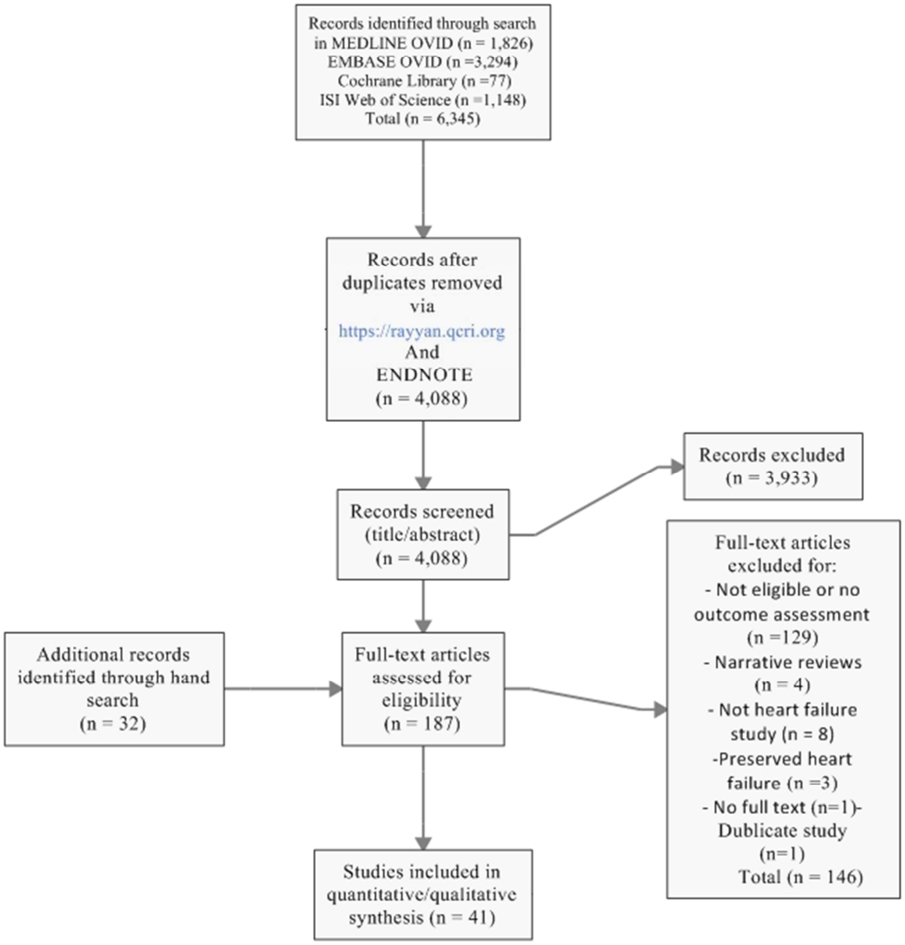

Fig 1: flow chart for search result

190x198mm (96 x 96 DPI)

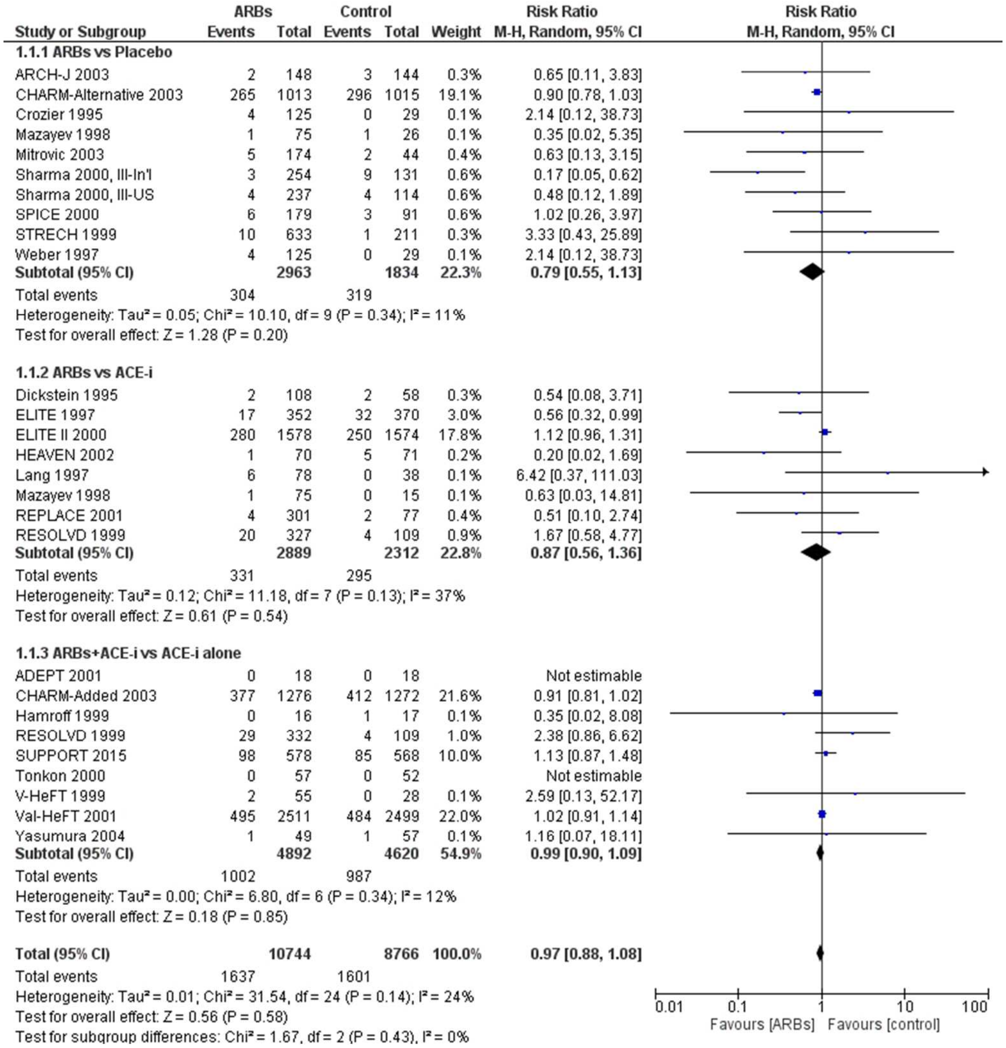

Fig 2: Efficacy of angiotensin receptor blockers (ARBs) compared with placebo, angiotensin-converting enzyme inhibitor (ACE-i), or combined in heart failure and reduced ejection fraction (HFrEF) for the prevention of all-cause mortality.

67x70mm (300 x 300 DPI)

Study or Subgroup	Experimental Events	Experimental Total	Control Events	Control Total	Weight	Risk Ratio M-H, Random, 95% CI
CHARM-Added 2003	150	1276	168	1272	24.5%	0.89 [0.72, 1.09]
CHARM-Alternative 2003	80	1013	111	1015	21.0%	0.72 [0.55, 0.95]
ELITE II 2000	130	1578	101	1574	22.2%	1.28 [1.00, 1.65]
SUPPORT 2015	18	578	8	568	5.7%	2.21 [0.97, 5.04]
Val-HeFT 2001	262	2511	258	2499	26.7%	1.01 [0.86, 1.19]
**Total (95% CI)**		**6956**		**6928**	**100.0%**	**1.01 [0.81, 1.25]**
Total events	640		646			

Heterogeneity: Tau² = 0.04; Chi² = 13.96, df = 4 (P = 0.007); I² = 71%
Test for overall effect: Z = 0.06 (P = 0.95)

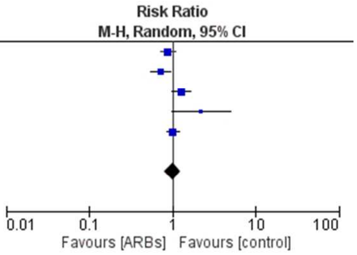

Fig 3: Efficacy of angiotensin receptor blockers (ARBs) compared with control in heart failure and reduced ejection fraction (HFrEF) for the prevention of sudden cardiac death (SCD).

67x17mm (300 x 300 DPI)

For peer review only

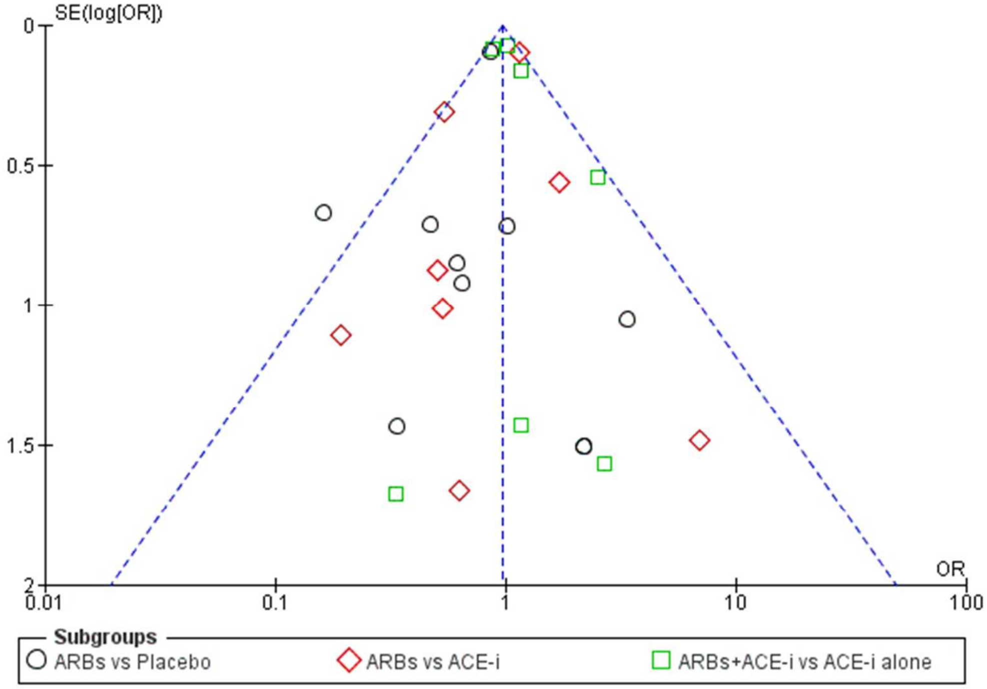

Fig 4: Funnel plot of SE (log odds ratio) by odds ratio to evaluate publication bias for the efficacy of angiotensin receptor blockers (ARBs) compared with control in heart failure and reduced ejection fraction (HFrEF) for the prevention of all-cause mortality.

50x36mm (300 x 300 DPI)

w only

S1 File: PRISMA Checklist

Section/topic	#	Checklist item	Reported on page #
**TITLE**			
Title	1	Identify the report as a systematic review, meta-analysis, or both.	1
**ABSTRACT**			
Structured summary	2	Provide a structured summary including, as applicable: background; objectives; data sources; study eligibility criteria, participants, and interventions; study appraisal and synthesis methods; results; limitations; conclusions and implications of key findings; systematic review registration number.	2
**INTRODUCTION**			
Rationale	3	Describe the rationale for the review in the context of what is already known.	4
Objectives	4	Provide an explicit statement of questions being addressed with reference to participants, interventions, comparisons, outcomes, and study design (PICOS).	4
**METHODS**			
Protocol and registration	5	Indicate if a review protocol exists, if and where it can be accessed (e.g., Web address), and, if available, provide registration information including registration number.	4
Eligibility criteria	6	Specify study characteristics (e.g., PICOS, length of follow-up) and report characteristics (e.g., years considered, language, publication status) used as criteria for eligibility, giving rationale.	5
Information sources	7	Describe all information sources (e.g., databases with dates of coverage, contact with study authors to identify additional studies) in the search and date last searched.	5
Search	8	Present full electronic search strategy for at least one database, including any limits used, such that it could be repeated.	S2 File
Study selection	9	State the process for selecting studies (i.e., screening, eligibility, included in systematic review, and, if applicable, included in the meta-analysis).	5
Data collection process	10	Describe method of data extraction from reports (e.g., piloted forms, independently, in duplicate) and any processes for obtaining and confirming data from investigators.	5
Data items	11	List and define all variables for which data were sought (e.g., PICOS, funding sources) and any assumptions and simplifications made.	5
Risk of bias in individual studies	12	Describe methods used for assessing risk of bias of individual studies (including specification of whether this was done at the study or outcome level), and how this information is to be used in any data synthesis.	6
Summary measures	13	State the principal summary measures (e.g., risk ratio, difference in means).	6
Synthesis of results	14	Describe the methods of handling data and combining results of studies, if done, including measures of consistency (e.g., I^2) for each meta-analysis.	6

For peer review only - http://bmjopen.bmj.com/site/about/guidelines.xhtml

S1 File: PRISMA Checklist

Section/topic	#	Checklist item	Reported on page #
Risk of bias across studies	15	Specify any assessment of risk of bias that may affect the cumulative evidence (e.g., publication bias, selective reporting within studies).	6
Additional analyses	16	Describe methods of additional analyses (e.g., sensitivity or subgroup analyses, meta-regression), if done, indicating which were pre-specified.	6
**RESULTS**			
Study selection	17	Give numbers of studies screened, assessed for eligibility, and included in the review, with reasons for exclusions at each stage, ideally with a flow diagram.	7
Study characteristics	18	For each study, present characteristics for which data were extracted (e.g., study size, PICOS, follow-up period) and provide the citations.	Table 1
Risk of bias within studies	19	Present data on risk of bias of each study and, if available, any outcome level assessment (see item 12).	Tables 1&2 and S3
Results of individual studies	20	For all outcomes considered (benefits or harms), present, for each study: (a) simple summary data for each intervention group (b) effect estimates and confidence intervals, ideally with a forest plot.	8-10
Synthesis of results	21	Present the main results of the review. If meta-analyses are done, include for each, confidence intervals and measures of consistency.	8-10
Risk of bias across studies	22	Present results of any assessment of risk of bias across studies (see Item 15).	S3 Table & Fig 4
Additional analysis	23	Give results of additional analyses, if done (e.g., sensitivity or subgroup analyses, meta-regression [see Item 16]).	9 (Fig 2-3)
**DISCUSSION**			
Summary of evidence	24	Summarize the main findings including the strength of evidence for each main outcome; consider their relevance to key groups (e.g., healthcare providers, users, and policy makers).	10-11
Limitations	25	Discuss limitations at study and outcome level (e.g., risk of bias), and at review-level (e.g., incomplete retrieval of identified research, reporting bias).	12
Conclusions	26	Provide a general interpretation of the results in the context of other evidence, and implications for future research.	13
**FUNDING**			
Funding	27	Describe sources of funding for the systematic review and other support (e.g., supply of data); role of funders for the systematic review.	14

From: Moher D, Liberati A, Tetzlaff J, Altman DG, The PRISMA Group (2009). Preferred Reporting Items for Systematic Reviews and Meta-Analyses: The PRISMA Statement. PLoS Med 6(7): e1000097. doi:10.1371/journal.pmed1000097. For more information, visit: **www.prisma-statement.org**.

For peer review only - http://bmjopen.bmj.com/site/about/guidelines.xhtml

Database(s): **Ovid MEDLINE(R) Epub Ahead of Print, In-Process & Other Non-Indexed Citations, Ovid MEDLINE(R) Daily and Ovid MEDLINE(R)** 1946 to 25/05/2017
Search Strategy: 24.05.2017

#	Searches	Results
1	exp heart failure/ or (((heart or cardia* or myocardial) adj3 failure*) or (((heart or cardia*) adj3 decompensation*) or ((heart or cardia*) adj3 insufficienc*))).mp.	189098
2	review.ab.	1050955
3	review.pt.	2299071
4	meta-analysis.ab.	82199
5	meta-analysis.pt.	80375
6	or/2-5	2743700
7	letter.pt.	971739
8	comment.pt.	690989
9	editorial.pt.	439919
10	or/7-9	1585046
11	6 not 10	2708683
12	1 and 11	39715
13	(sudden adj3 (cardiac or death*)).ti,ab,kw.	42355
14	12 and 13	1826

For peer review only

Database(s): **Embase** 1974 to 2017 May 23rd
Search Strategy:

#	Searches	Results
1	exp heart failure/ or (((heart or cardia* or myocardial) adj3 failure*) or (((heart or cardia*) adj3 decompensation*) or ((heart or cardia*) adj3 insufficienc*))).mp.	444143
2	meta-analy:.mp.	202659
3	search:.tw.	417814
4	review.pt.	2246810
5	or/2-4	2642821
6	exp Sudden Cardiac Death/ or Sudden Death/ or exp heart arrest/	107134
7	5 and 6	14446
8	1 and 7	9747
9	(sudden adj3 (cardiac or death*)).mp.	76511
10	limit 9 to abstracts	58853
11	8 and 10	3294

For peer review only - http://bmjopen.bmj.com/site/about/guidelines.xhtml

S3 Table: AMSTAR scores for included studies

For peer review only

Author (year); country; reference	Q 1	Q 2	Q 3	Q 4	Q 5	Q 6	Q 7	Q 8	Q 9	Q 10	Q 11	AMSTAR score
Al-Gobari M., et al. (2013), France [14]	No	No	Yes	No	No	Yes	Yes	Yes	Yes	Yes	No	6
Chatterjee S., et al. (2013); USA [13]	No	Yes	Yes	Yes	No	Yes	Yes	Yes	Yes	Yes	No	8
Brophy JM. Et al., (2001); Canada [15]	No	No	Yes	No	No	Yes	No	No	Yes	Yes	No	4
Lee S. et al., (2001), USA [16]	No	No	Yes	Yes	No	Yes	No	No	Yes	No	No	4
Bonet S., et al. (2000), USA [17]	No	Yes	Yes	No	No	Yes	No	No	Yes	No	No	4
Heidenreich P.A., et al. (1997); USA [18]	No	Yes	No	Yes	Yes	Yes	No	No	Yes	No	No	5
Le HH. et al., (2016); France [19]	No	Yes	Yes	No	No	Yes	Yes	Yes	Yes	Yes	No	7
Bapoje SR. et al. (2013); USA [52]	No	Yes	Yes	Yes	No	Yes	Yes	Yes	Yes	Yes	No	8
Wei J., et al. (2010); China [20]	No	Yes	Yes	Yes	No	Yes	No	No	Yes	No	No	5
Solomon SD., et al. (2016); USA [21]	No	Yes	Yes	No	No	Yes	Yes	Yes	yes	No	Yes	7
Flather MD et al., (2000); Canada [23]	No	No	Yes	No	No	Yes	No	No	Yes	No	No	3
Garg R. et al., (1995); Canada [22]	No	Yes	No	No	No	No	No	No	Yes	No	No	2
Rain & Rada, (2015) ; Chile [24]*												NA*
Heran et al., (2012) ; Canada [25]	Yes	Yes	Yes	No	Yes	Yes	Yes	Yes	Yes	Yes	Yes	10
Shibata et al., (2008) ; Canada [26]	No	Yes	No	Yes	No	Yes	No	No	Yes	No	No	4
Lee et al., (2004) ; USA [27]	No	Yes	Yes	Yes	No	Yes	Yes	No	Yes	Yes	No	7
Dimopoulos et al., (2004) ; UK [28]	No	Yes	No	No	No	Yes	No	No	Yes	No	No	3
Jong P., et al. (2002), Canada [29]	Yes	Yes	Yes	No	No	Yes	Yes	Yes	Yes	Yes	No	8
Rain & Rada, (2017); Chile [30]*												NA*
Al-Gobari M., et al. (2017), Switzerland [5]	No	Yes	Yes	No	No	Yes	Yes	Yes	Yes	Yes	No	7
Bonsu KO, et al. (2015), Malaysia [31]	Yes	Yes	Yes	No	No	Yes	Yes	Yes	Yes	Yes	No	8

For peer review only

Wang JQ., et al. (2014); China [32]	No	Yes	Yes	No	No	Yes	Yes	No	No	Yes	No	5
Liu G. et al. (2014); China [33]	No	Yes	Yes	No	No	Yes	Yes	Yes	Yes	Yes	No	7
Rahimi K. et al. (2012); UK [38]	No	Yes	Yes	No	No	Yes	Yes	Yes	Yes	No	No	6
Zhang S. et al. (2011); China [34]	No	Yes	Yes	No	No	Yes	Yes	Yes	Yes	Yes	No	7
Xu M. et al. (2010); China [35]	No	Yes	Yes	Yes	No	Yes	No	No	Yes	Yes	No	6
Lipinski MJ. (2009); USA [36]	No	Yes	Yes	No	No	Yes	Yes	Yes	Yes	Yes	No	7
Levantesi G. et al. (2007); Italy [37]	No	Yes	No	No	No	Yes	No	No	Yes	No	No	3
Claro JC., et al. (2015), Chile [39]	Yes	Yes	Yes	Yes	Yes	Yes	Yes	Yes	Yes	Yes	No	10
Santangeli P. et al. (2012); USA [40]	No	Yes	Yes	Yes	No	Yes	No	No	Yes	No	No	5
Piccini JP., et al. (2009); USA [41]	No	Yes	Yes	No	No	Yes	Yes	Yes	Yes	Yes	No	7
ATMA Investigators (1997) [42]	Can't answer	Yes	No	No	No	Yes	No	No	Yes	No	No	3
Sim I. et al., (1997); USA [43]	No	Yes	Yes	Yes	No	Yes	No	No	Yes	No	No	5
Das MK. et al., (2010): USA [44]*												NA*
Hilleman DE. et al., (2001); USA [45]*												NA*
Rizos EC., et al. (2012); Greece [46]	No	Yes	Yes	No	No	Yes	Yes	Yes	Yes	Yes	Yes	8
Kotwal S. et al. (2012); Australia [47]	No	Yes	Yes	No	No	Yes	Yes	Yes	Yes	Yes	No	7
Kwak SM., et al. (2012); Korea [48]	No	Yes	Yes	No	No	Yes	Yes	Yes	Yes	Yes	Yes	8
Chen Q., et al. (2011); China [49]	No	Yes	Yes	No	No	Yes	Yes	Yes	Yes	Yes	No	7
Marik PE., et al. (2009); USA [50]	No	Yes	Yes	No	No	Yes	No	No	Yes	No	No	4
Wang C., et al. (2006); USA [51]	No	No	Yes	Yes	No	Yes	Yes	Yes	Yes	No	No	6

For peer review only

Q 1: Was an 'a priori' design provided?

Q 2: Was there duplicate study selection and data extraction?

Q 3: Was a comprehensive literature search performed?

Q 4: Was the status of publication (i.e. grey literature) used as an inclusion criterion?

Q 5: Was a list of studies (included and excluded) provided?

Q 6: Were the characteristics of the included studies provided?

Q 7: Was the scientific quality of the included studies assessed and documented?

Q 8: Was the scientific quality of the included studies used appropriately in formulating conclusions?

Q 9: Were the methods used to combine the findings of studies appropriate?

Q 10: Was the likelihood of publication bias assessed?

Q 11: Was the conflict of interest included?

* narrative reviews or overview (not applicable)

For peer review only

For peer review only

S4 Table: Funding data of included reviews of heart failure patients

Author (year); country; reference	Review type	Intervention/ Comparator	Funding
Al-Gobari M., et al. (2013), France [14]	Systematic review & meta-analysis	Beta-blockers /Placebo; "usual care"	YEMEN LNG company: Private company but non-drug-industry., UMR 5558, BEBL.
Chatterjee S., et al. (2013); USA [13]	Systematic review & meta-analysis	Beta-blockers / Placebo; beta-blocker; "usual care"	None
Brophy JM. Et al., (2001); Canada [15]	Meta-analysis	Beta-blockers / Placebo; "usual care"	Les Fonds de la Recherche en Santé du Québec. Government (non-industry)
Lee S. et al., (2001), USA [16]	systematic review & meta-analysis	Beta-blockers / Placebo	No data reported
Bonet S., et al. (2000), USA [17]	Meta-analysis	Beta-blockers / Placebo ; "usual care"	No data reported
Heidenreich P.A., et al. (1997); USA [18]	Meta-analysis	Beta-blockers / Placebo; "usual care"	No data reported
Le HH. et al., (2016); France [19]	Systematic review & meta-analysis	Anti-aldosterone / Placebo; "usual care"	Non-industry (Academic and Government)
Bapoje SR. et al. (2013); USA [52]	SR and MA	Anti-aldosterone / Placebo; "usual care"	Not reported for included studies. No external fund for the review.
Wei J., et al. (2010); China [20]	Meta-analysis	Anti-aldosterone / Placebo; "usual care"	No data reported
Solomon SD., et al. (2016); USA [21]	Meta-analysis	Sacubitril; valsartan / ACE-i	Data reported for included studies: industry. No funding existed for the review itself.
Flather MD et al., (2000); Canada [23]	Systematic review	ACE-i / Placebo	Industry support: Medical Research Council of Canada and support from Merck Frosst Canada, Merck Sharpe and Dohme, USA, Bristol Myers, Squibb, USA, Zeneca, UK, and Hoechst Marion Roussel, Frankfurt, Germany.
Garg R. et al., (1995); Canada [22]	Systematic review & meta-analysis	ACE-i / Placebo	No data reported
Rain & Rada, (2015) ; Chile [24]	Systematic review	ARB/ ACE-i	No data reported
Heran et al., (2012) ; Canada [25]	Systematic review & meta-analysis (Cochrane)	ARB (or ARB + ACE-i) / Placebo ; ACE-i	Included studies: 23 (96%) industry-sponsored and remaining unknown. Review. External: NIHR, UK Cochrane (Non-industry)
Shibata et al., (2008) ; Canada [26]	Systematic review & meta-analysis	ARB/ Placebo ; ACE-i	No funding exists. Not reported for included studies.
Lee et al., (2004) ; USA [27]	Meta-analysis	ARB/ Placebo ; ACE-i	Industry support: Stock ownership or options (other than mutual funds): D.C. Rhew (Merck, Pfizer); Grants received: Zynx Health Incorporated has received grants from AstraZeneca, Aventis, Bristol-Myers Squibb, Merck, Novartis, and Pfizer.
Dimopoulos et al., (2004) ; UK [28]	Meta-analysis	ARB/ Placebo ; ACE-i	No data reported
Jong P., et al. (2002), Canada [29]	Systematic review & meta-analysis	ARB (or ARB + ACE-i) / Placebo ; ACE-i	No data reported
Rain & Rada, (2017); Chile [30]	systematic review	Statins / Placebo ; "usual care"	No data reported
Al-Gobari M., et al. (2017), Switzerland [5]	Systematic review & meta-analysis	Statins / Placebo ; "usual care"	Yes for the review: non-industry (Governments). Not reported for included studies.

For peer review only

Bonsu KO, et al. (2015), Malaysia [31]	Meta-analysis	Statins / Placebo ; "usual care"	No data reported
Wang JQ., et al. (2014); China [32]	Meta-analysis	Statins / Placebo ; "usual care"	None (reported) but not for included studies.
Liu G. et al. (2014); China [33]	Meta-analysis	Statins / Placebo ; "usual care"	Non-industry-sponsored but no data for included studies.
Rahimi K. et al. (2012); UK [38]	Meta-analysis	Statins / Placebo ; "usual care"	Funding for included studies reported. Some are industry. No funding for the review itself from industry but from Academy for some authors.
Zhang S. et al. (2011); China [34]	Meta-analysis	Statins / Placebo ; "usual care"	Non-industry-sponsored but no data for included studies. Academic, doctoral program…etc
Xu M. et al. (2010); China [35]	Meta-analysis	Statins / Placebo ; "usual care"	No data reported
Lipinski MJ. (2009); USA [36]	Meta-analysis	Statins / Placebo ; "usual care"	No data reported
Levantesi G. et al. (2007); Italy [37]	Meta-analysis	Statins / Placebo ; "usual care"	No data reported
Claro JC., et al. (2015), Chile [39]	Systematic review & meta-analysis (Cochrane)	Amiodarone / Placebo; "usual care"	Funding for the review: Government (non-industry). Not reported for included studies.
Santangeli P. et al. (2012); USA [40]	systematic review	Amiodarone / Placebo	No data reported
Piccini JP., et al. (2009); USA [41]	Meta-analysis	Amiodarone / Placebo ; "usual care"	No external fund for the review. No data reported for included studies.
ATMA Investigators (1997) [42]	Meta-analysis	Amiodarone / Placebo ; "usual care"	No data reported
Sim I. et al., (1997); USA [43]	Meta-analysis	Amiodarone / Placebo ; "usual care"	No data reported
Das MK. et al., (2010): USA [44]	Narrative Review	Anti-arrhythmics / Placebo ; "usual care"	No data reported
Hilleman DE. et al., (2001); USA [45]	Narrative Review	Anti-arrhythmics / Placebo ; "usual care"	No data reported
Rizos EC., et al. (2012); Greece [46]	systematic review & meta-analysis	Omega 3 Fatty acids / Placebo ; "usual care"	Funding of included studies is reported. Conflict of interest too (for one author): industry sponsorship not related to those who manufacture or market Omega-3 suppl.
Kotwal S. et al. (2012); Australia [47]	systematic review & meta-analysis	Omega 3 Fatty acids / Placebo ; "usual care"	Reported but not for included studies. Non-industry.
Kwak SM., et al. (2012); Korea [48]	Meta-analysis	Omega 3 Fatty acids / Placebo ; "usual care"	All included trials sponsored by industry but not reported for the review itself.
Chen Q., et al. (2011); China [49]	Meta-analysis	Omega 3 Fatty acids / Placebo ; "usual care"	No data reported
Marik PE., et al. (2009); USA [50]	systematic review	Omega 3 dietary supplements / Placebo; olive oil ; corn oil, sunflower oil ; "usual care"	No data reported
Wang C., et al. (2006); USA [51]	systematic review	n-3 Fatty acids / Placebo/olive oil ; corn oil, sunflower oil ; "usual care"	Internal by saying who obtained funds but no specific source. No data for included studies.

For peer review only

BMJ Open

Effectiveness of drug interventions to prevent sudden cardiac death in patients with heart failure and reduced ejection fraction: an overview of systematic reviews

Journal:	*BMJ Open*
Manuscript ID	bmjopen-2017-021108.R1
Article Type:	Research
Date Submitted by the Author:	12-Apr-2018
Complete List of Authors:	Al-Gobari, Muaamar; Lausanne university Hospital (CHUV), Institute of social & preventive medicine (IUMSP), Cochrane Switzerland<br>Al-Aqeel, Sinaa; King Saud University , Clinical Pharmacy<br>Gueyffier, François; Université Claude Bernard Lyon 1, UMR5558; Hospices Civils de Lyon, Service de Pharmacologie clinique<br>Burnand, Bernard; Lausanne university Hospital (CHUV), Institute of social & preventive medicine (IUMSP), Cochrane Switzerland
**Primary Subject Heading**:	Cardiovascular medicine
Secondary Subject Heading:	Evidence based practice
Keywords:	cardiac failure, sudden death, umbrella review, meta-analysis, guidelines, treatment

SCHOLARONE™ Manuscripts

only

For peer review only - http://bmjopen.bmj.com/site/about/guidelines.xhtml

Effectiveness of drug interventions to prevent sudden cardiac death in patients with heart failure and reduced ejection fraction: an overview of systematic reviews

Muaamar Al-Gobari[1*], Sinaa Alaqeel[2], François Gueyffier[3], Bernard Burnand[1]

[1] Institute of social & preventive medicine (IUMSP) and Cochrane Switzerland, Lausanne University Hospital (CHUV), Lausanne, Switzerland

[2] Clinical Pharmacy Department, College of Pharmacy, King Saud University, Riyadh, Kingdom of Saudi Arabia.

[3] Laboratoire de Biologie et Biométrie Evolutive-Equipe Modélisation des Effets Thérapeutiques, UMR 5558 Université Claude Bernard Lyon1, Lyon, France

*** Corresponding Author Information:**

Institute of social & preventive medicine (IUMSP)

Biopôle 2, Route de la Corniche 10

CH-1010 Lausanne, Switzerland

Tel. +41 21 314 51 45

E-mail: muaamar.algobari@gmail.com

For peer review only

For peer review only - http://bmjopen.bmj.com/site/about/guidelines.xhtml

Abstract

Objectives

To summarize and synthesize the current evidence regarding the effects of drug interventions on prevention of sudden cardiac death (SCD) and all-cause mortality in heart failure with reduced ejection fraction (HFrEF) patients.

Design

Overview of systematic reviews.

Data sources

Medline, Embase, ISI Web of Science and Cochrane Library from inception to May 2017; manual search of references of included studies for potentially relevant reviews.

Eligibility criteria for study selection

We reviewed drug interventions in HFrEF patients that evaluated the effectiveness on SCD and all-cause mortality prevention, including overviews, systematic reviews and meta-analyses of randomized controlled trials of beta-blockers, angiotensin-converting enzyme inhibitors (ACE-i), angiotensin receptor blockers (ARBs), anti-aldosterones or mineralocorticoid-receptor antagonists, amiodarone, other antiarrhythmic drugs, combined angiotensin receptor blocker (ARB)/neprilysin inhibitors, statins and fish oil supplementation.

Review methods

Two independent reviewers extracted data and assessed the methodological quality of the reviews and the quality of evidence for the primary studies for each drug intervention, using AMSTAR and GRADE respectively.

Results

We identified 41 reviews. Beta-blockers, anti-aldosterones, and combined ARB/neprilysin inhibitors appeared effective to prevent SCD and all-cause mortality, angiotensin-converting enzyme inhibitors significantly reduced all-cause mortality but not SCD events, angiotensin receptor blockers and statins were ineffective where antiarrhythmic drugs and omega-3 fatty acids had unclear evidence of effectiveness for prevention of SCD and all-cause mortality.

For peer review only - http://bmjopen.bmj.com/site/about/guidelines.xhtml

Conclusions

This comprehensive overview of the evidence confirms that beta-blockers, anti-aldosterone agents and combined ARB/neprilysin inhibitors are effective on SCD prevention but not ACE-i or ARBs. In patients with high risk of SCD, an alternative therapeutic strategy should be explored in future research.

Systematic Review Registration: PROSPERO 2017: CRD42017067442

Key Words

cardiac failure, sudden death, umbrella review, treatment, pharmacotherapy, meta-analysis, guidelines, evidence-based medicine

Strengths and limitations of this study

- A major strength of our study is that it summarizes and synthesizes the effectiveness of most evidence-based drug interventions in heart failure patients with reduced ejection fraction for sudden cardiac death (SCD) prevention and classified drug interventions according to the current evidence of their effectiveness.
- Our study used data from published studies and no data from unpublished studies.
- Our study reviews most heart failure drugs on the prevention of SCD and all-cause mortality but limited in scope for not including some drugs such as digoxin, ivabradine, and non-drug devices such as implantable cardioverter defibrillators.

For peer review only

Introduction

Heart failure (HF) morbidity and mortality constitute an important burden for patients and for the healthcare systems in both developed and developing countries [1]. HF patients are frequently hospitalized and not only have a high mortality risk from a bad prognosis, but also unexpectedly, termed sudden cardiac death (SCD). In people diagnosed with HF, SCD occurs at 6–9 times the rate of the general population. Almost 20% and 80% of patients die within one year and eight years of initial diagnosis, respectively [1 2]. Potential drug interventions in heart failure with reduced ejection fraction (HFrEF) patients include beta-blockers (BBs), angiotensin-converting enzyme inhibitors (ACE-i), angiotensin receptor blockers (ARBs), anti-aldosterones or mineralocorticoid-receptor antagonists, amiodarone, other antiarrhythmic agents, combined angiotensin receptor blocker/neprilysin inhibitors, statins and fish oil supplementation [3]. Some of these interventions aimed at improving survival and reducing total mortality and SCD in HF. For instance, a newly licensed drug (Sacubitril/valsartan) in Paradigm-HF trial showed around 20% SCD reduction compared to enalapril [4].

Nevertheless, optimal strategies for SCD prevention in HF are warranted if we take into account the high portion of mortality that still occurs in this population. Had a practitioner identified a patient with high risk of SCD, it would be important to know which drug is effective or not in SCD prevention. However, the large amount of information and the multiple and sometimes discordant systematic reviews could be misleading [5].

Therefore, there is a need to further review and investigate if all these drug interventions prevent SCD events in high-risk patients, whether one treatment is preferable to another, or if any optimization of therapeutic strategies to those patients is possible accordingly. Thus, we decided to conduct an overview of systematic reviews to summarize and synthesize the available evidence of the effectiveness of drug interventions in the prevention of SCD in HFrEF, and categorized the evidence into effective, ineffective and unclear evidence of effectiveness.

Methods

We developed an a priori protocol for this review according to the Preferred Reporting Items for Systematic Reviews and Meta-Analyses (PRISMA) statement (**S1 File**) and registered it in the PROSPERO International prospective register of systematic reviews (CRD42017067442).

For peer review only - http://bmjopen.bmj.com/site/about/guidelines.xhtml

Data sources and search strategy

Using the Ovid online interface, we searched Medline (up to May 24, 2017), Embase (up to May 23, 2017), ISI web of science and the Cochrane library (up to May 24, 2017). We identified overviews, systematic reviews and meta-analyses of randomized clinical trials by means of a search strategy (available on **S2 File**). The search strategy was composed of a filter [6 7], a mixture of medical subject heading terms [Mesh and EMTREE in Medline and Embase respectively], text words as well as a truncation when possible without any language or publication date restriction. We did not search conference proceedings nor the grey literature. Reference lists of the included reviews were manually checked for any additional eligible studies. We contacted corresponding reviews' and primary studies' authors in the case of relevant unreported data. If judged necessary, we intended to update the included reviews by searching primary studies published after the systematic review publication date. Apart from authors' expertise in the field, we decided to update if the most up-to-date review of a drug intervention was published more than 5 years ago and/or new clinical trials are not integrated into the evidence.

Selection criteria and data abstraction

Studies were eligible if they were overviews, systematic reviews and meta-analyses of randomized clinical trials that evaluated the effectiveness of drug interventions in HFrEF patients. Reviews were included if they examined the effectiveness of the following drugs: BBs, ACE-i, ARBs, anti-aldosterones or mineralocorticoid-receptor antagonists, amiodarone, antiarrythmics, combined ARB/neprilysin inhibitors, statins and fish oil supplementation. The selected reviews should have contained at least one of the aforementioned HF therapy and had evaluated SCD and/or all-cause mortality prevention as outcomes. We used Endnote and Rayyan[8] to remove duplicates during the selection based on titles and abstracts, and full-text screening.

The abstracted data included eligibility criteria, population type, ejection fraction, study design (including intervention and comparator arms), follow-up duration, and authors' evaluation of outcomes. Two reviewers (MA&SA) independently abstracted data. We resolved discrepancies by consensus or by adding a third reviewer's judgment, when necessary.

Quality assessment of the included reviews

Methodological quality of the included reviews

Two authors independently used the AMSTAR measurement tool to assess systematic reviews included in our overview. The AMSTAR checklist comprises eleven questions (**S3 table**) and each

For peer review only - http://bmjopen.bmj.com/site/about/guidelines.xhtml

question accounted for one score point [9]. The answer of "yes" gave a score of 1 and zero otherwise. This increasingly adopted tool was used at the data collection step as stipulated in the overview protocol[10]. If the authors of included reviews failed to publish their protocol, we deducted a score of one. In addition, we scored "yes" if the authors mentioned that two reviewers were involved in the study screening, selection or data extraction.

Quality of evidence in the included reviews

Two authors independently used the GRADE (Grading of Recommendations, Assessment, Development and Evaluation) approach [11] to assess the quality of evidence of each intervention. GRADE is a widely accepted tool that allows the assessment of five key elements: risk of bias, inconsistency, indirectness, imprecision, and publication bias. GRADE categorizes the quality of evidence into four levels: high, moderate, low and very low. In the presence of a high risk of bias, the quality of the evidence is downgraded from high to moderate and so on. We also reported the GRADE assessments reported by the authors of the included reviews, or assessed them otherwise. Moreover, we did not reassess the risk of bias at primary study level if authors of included reviews had sufficiently assessed their quality. In the case of the updated review of angiotensin receptor blockers, however, we assessed the quality of newly added randomized clinical trials and integrated it into the evidence synthesis.

Statistical analysis and data synthesis

We provided a narrative synthesis of the findings of the included reviews and if multiple reviews existed for the same intervention. However, in the case of ARBs, we updated the evidence and meta-analyzed the data using random-effects and fixed-effects model with Mantel-Haenszel methods [12] and reported random-effects model to account for heterogeneity. Meanwhile, we evaluated each intervention against our outcomes of interest and synthesized the evidence taking into account heterogeneity and inconsistencies between reviews. As a rule of thumb, I^2 (I-square) values of 25%, 50% and 75% correspond to low, moderate and high levels of heterogeneity, respectively [13].

For the purpose of our overview, we categorized the evidence of the included interventions into three categories: 1) effective interventions; 2) ineffective interventions; 3) uncertain evidence (conflicting or inconclusive evidence). We used relative effect [odds ratio (OR) or risk ratio (RR)] as a summary statistic from the most recent or largest published systematic reviews, and confidence intervals (CIs) of 95% with a significance level determined at two-sided alpha less than 5%.

For peer review only - http://bmjopen.bmj.com/site/about/guidelines.xhtml

Patient and Public Involvement

Our study did not involve direct contact with patients or the public

Results

Search result

According to our pre-defined eligibility criteria, our search strategy in electronic databases and manual searches resulted in 41 studies [5 14-53]. Fig 1 shows the search strategy results. At full-text level, we excluded studies that did not assess our outcome of interest (n=129), were narrative reviews (n=4), did not include HF patients (n=8), included preserved heart failure patients (n= 3), were duplicate or had no full text (n=2).

Characteristics of the included reviews

As shown in **Table 1**, the population of the included reviews consisted of HF patients with an ejection fraction ≤ 45% in most studies and a corresponding New York Heart Association (NYHA) classification ranging from I-IV. The effectiveness of each drug intervention has been assessed in at least one review. All reviews were systematic, except two reviews for anti-arrhythmic drugs. At the time of their publication, 15 out of 41 reviews (37%) had corresponding authors based in the USA, 7 (17%) in Canada, 6 (15%) in China, 3 in Chile, 2 in France, 2 in the UK, and the 6 remaining in other countries.

The disclosure and reporting of financial resources or funding varied from one study to another. Twenty-one reviews (51%) did not report the source of funding. Ten reviews (24 %) reported financial supports that included governments, academic institutions, and device industry. Six reviews declared financial resources as none or no external funds. Three reviews reported industry sponsorship for at least one author. One review [52] stated that one author obtained funds for the review without clarifying the source (**S4 table**). We also reported findings summary of each review as stated by their respective authors (**Table 1**).

For peer review only - http://bmjopen.bmj.com/site/about/guidelines.xhtml

Table 1: Summary of characteristics of included studies of heart failure (ordered by intervention)

Author (year); country; reference	Review type	Intervention/ Comparator	Population type; Ejection fraction (%); NYHA	Study design n; participants n	Mean follow-up/Range (months)	Authors' Findings summary	AMSTAR score
Al-Gobari M., et al. (2013), France [15≠]	Systematic review & meta-analysis	Beta-blockers /Placebo; "usual care"	HF; <45% except one study < 62%; I-IV	RCTs n= 30; n = 24,779	Mean: 11.51	Beta-blockers significantly reduced SCD, cardiovascular death and all-cause mortality	6
Chatterjee S., et al. (2013); USA [14]	Systematic review & meta-analysis	Beta-blockers / Placebo; beta-blocker; "usual care"	HF; <45%; II-IV	RCTs n=21 ; n= 23,122	median: 12	The study confirmed mortality benefits of BBs compared to placebo or usual care in heart failure with reduced ejection fraction.	8
Brophy JM. Et al., (2001); Canada [16]	Meta-analysis	Beta-blockers / Placebo; "usual care"	CHF; <45%; I-IV	RCTs n= 22 ; n= 10,135	Range: 3 to 23	This study reported a reduction in mortality and morbidity in CHF.	4
Lee S. et al., (2001), USA [17]	systematic review & meta-analysis	Beta-blockers / Placebo	HF; <30%; II-III	RCTs n=6 ; n= 9335	Range : 12 to 23	The authors recommended use of beta-blockers in HF with reduced ejection fraction and NYHA II-III.	4
Bonet S., et al. (2000), USA [18]	Meta-analysis	Beta-blockers / Placebo ; "usual care"	HF; <45%; NA	RCTs n= 21 ; n= 5,849	Median: 6	Beta-blockers reduce total mortality by reducing pump failure and sudden cardiac death events. Vasodilating beta-blockers have perhaps greater effects on overall mortality than nonvasodilating agents.	4
Heidenreich P.A., et al. (1997); USA [19]	Meta-analysis	Beta-blockers / Placebo; "usual care"	HF; <30%; I-IV	RCTs n= 17 ; n= 3,039	Range: 3 to 24	Beta-blockers significantly reduced all-cause mortality but showed a trend toward better reduction in nonsudden cardiac death compared to sudden cardiac death.	5
Le HH. et al., (2016); France [20≠]	Systematic review & meta-analysis	Anti-aldosterone / Placebo; "usual care"	HF, post-MI; <40%- >50%; I-IV	RCTs n=25; n = 19,333	Range: 3 to 39.6	In HF, anti-aldosterones or mineralocorticoid receptor blockers reduced SCD (subgroup analysis: 5 RCTs), all-cause mortality (subgroup analysis: 10 RCTs) and cardiovascular, all-cause and cardiovascular hospitalization. Adverse effects (hyperkalemia, degradation of renal function, gynecomastia) were, however, significantly higher in the treated group compared to placebo.	7
Bapoje SR. et al. (2013); USA [53]	Systematic review & meta-analysis	Anti-aldosterone / Placebo; "usual care"	HF; <45%; I-IV	RCTs n= 8; n= 11,875	Range: 3 to 24	Mineralocorticoid receptor antagonists (or aldosterone antagonists) reduced the risk of SCD in patients with left ventricular dysfunction.	8
Wei J., et al. (2010); China [21]	Meta-analysis	Anti-aldosterone / Placebo; "usual care"	HF; <45%; NA1	RCTs n= 6 (two are not double blind), n= 00000	Range: 2 to 24	Two [Pitt et al, 1995 and Pitt et al 2003] of the six-included studies showed a significant reduction of SCD in the group of Spironolactone vs placebo and the group of Eplerenone vs. Placebo, cited respectively.	5
Solomon SD., et al. (2016); USA [22≠]	Meta-analysis	Sacubitril; valsartan / ACE-i	HF; <30%; II-IV	RCTs n= 3; n= 14742	Range 6 to 27	The authors concluded that combined neprilysin/RAS inhibition reduced all-cause mortality in HFrEF.	7
Flather MD et al., (2000); Canada [24]	Systematic review	ACE-i / Placebo	CHF; post-MI <45%; NA	RCTs n= 5, n= 12,763	Range: 15 to 42	This meta-analysis showed a lower risk of death in ACE-i treated group compared to placebo.	NA
Garg R. et al., (1995); Canada [23≠]	Systematic review & meta-analysis	ACE-i / Placebo	CHF; <45% ; I-IV	RCTs n= 32, n= 7,105	Range: 3 to 42	Overall, this study reported a significant reduction of total mortality (attributed mainly to less progressive HF deaths) and hospitalization for worsening HF.	2

For peer review only
For peer review only - http://bmjopen.bmj.com/site/about/guidelines.xhtml

Rain & Rada, (2015) ; Chile [25]	Systematic review	ARB/ ACE-i	HF ; <45% - < 35% ; II-IV	RCTS = 8 ; n= 5201	NA	The authors concluded that ARBs are probably as effective in mortality reduction as ACE-i with probably less withdrawal rate due to adverse effects.	NA
Heran et al., (2012) ; Canada [26*]	Systematic review & meta-analysis (Cochrane)	ARB (or ARB + ACE-i) / Placebo ; ACE-i	HF ; ≤40% ; II-IV	RCTS n= 24 ; n= 25,051	Range: 1 to 49.5	Compared to placebo or in addition to ACE-i, ARBs did not reduce all-cause mortality.	10
Shibata et al., (2008) ; Canada [27]	Systematic review & meta-analysis	ARB/ Placebo ; ACE-i	HF ; <40% ; I-IV	RCTs n= 7 ; n= 27,495	Range: 11 to 41	Compared to ACE-i or used in combination, ARBs provided no beneficial effects on mortality. A 17% reduction in hospitalizations was observed.	4
Lee et al., (2004) ; USA [28]	Meta-analysis	ARB/ Placebo ; ACE-i	CHF , AMI ; ≤45% ; II–IV	RCTs n= 24 ; n= 38,080	Range: 1 to 41	Compared to ACE-i, ARBs do not differ in efficacy for reducing all-cause mortality in CHF and AMI patients.	7
Dimopoulos et al., (2004) ; UK [29]	Meta-analysis	ARB/ Placebo ; ACE-i	CHF; <40% ; II–IV	RCTs n= 4; n = 7666	Mean: 31	ARBs can be used to prevent events in ACE-i-treated heart failure patients who are not suitable for beta-blockers.	3
Jong P., et al. (2002), Canada [30]	Systematic review & meta-analysis	ARB (or ARB + ACE-i) / Placebo ; ACE-i	HF; ≤35% - ≤45%; II–IV	RCTs n= 17; n = 12,469	Range: 1 to 23	The authors could not conclude any superiority of ARBs versus controls, stating this might be due to the use of ACE-i as a comparator or background treatment in the majority of included trials.	8
Rain & Rada, (2017); Chile [31]	systematic review	Statins / Placebo ; “usual care”	HF; <45%; I-IV	RCTs n= 25; n = NR	NA	The authors summarized that statins do not decrease mortality in chronic heart failure and might lead to a small reduction in hospital admissions for HF.	NA
Al-Gobari M., et al. (2017), Switzerland [5≠]	Systematic review & meta-analysis	Statins / Placebo ; “usual care”	HF, ischaemic/nonischaemic; NA; I-IV NA	RCTs n= 24; n = 11,463	Range: 1 to 46.8	Statins do not significantly reduced SCD and all-cause mortality. They may or may not reduce hospitalizations due to worsening heart failure (HWHF)	7
Bonsu KO, et al. (2015), Malaysia [32]	Meta-analysis	Statins / Placebo ; “usual care”	HF; <45%; I-IV	RCTs n= 13; n = 10,966	Range: 3 to 46.8	Lipophilic statins showed significant decrease in all-cause mortality, cardiovascular mortality and hospitalization for worsening heart failure.	8
Wang JQ., et al. (2014); China [33]	Meta-analysis	Statins / Placebo ; “usual care”	HF; NA; NA	RCTs n= 6 (9: observational studies), n= 10,016	Range: 12 to 46.8	The authors concluded that statins reduce sudden cardiac death and all-cause mortality in HF.	5
Liu G. et al. (2014); China [34]	Meta-analysis	Statins / Placebo ; “usual care”	HF; <45%; I-IV	RCTs n=13 ; n = 1,532	Range: 3 to 35.5	The authors reported significant decrease in all-cause mortality but recommended cautious interpretation and further research.	7
Rahimi K. et al. (2012); UK [39]	Meta-analysis	Statins / Placebo ; “usual care”	HF, MI, Primary prevention, diabetes, ACS, CHD; NA; NA	RCTs n= 37 n= 155,020		Statins have a modest effect on SCD but no substantial protective effect on ventricular arrhythmic events.	6
Zhang S. et al. (2011); China [35]	Meta-analysis	Statins / Placebo ; “usual care”	HF; <45%; I-IV	RCTs n=13 ; n = 10,447	Range: 2 to 46.8	This meta-analysis concluded of no difference between treatment groups but benefits may occur in some specific populations and with a specific statin.	7
Xu M. et al. (2010); China [36]	Meta-analysis	Statins / Placebo ; “usual care”	HF; <45%; I-IV	RCTs n= 7, n= 540	Range: 3 to 31	The authors suggested that atorvastatin treatment is effective and reduce all-cause mortality and hospitalization for worsening HF.	6
Lipinski MJ. (2009); USA [37]	Meta-analysis	Statins / Placebo ; “usual care”	HF; <45%; I-IV	RCTS n= 10; n= 10,192	Range 3 to 47	The authors stated that statins are safe and improve LVEF and decrease hospitalization for worsening HF.	7
Levantesi G. et al. (2007); Italy [38]	Meta-analysis	Statins / Placebo ; “usual care”	Secondary prevention; NA; NA	RCTs n= 10 n= 22,275	Range: 6 to 73.2	Statins were associated with a significant risk reduction for SCD (in secondary prevention settings).	3
Claro JC., et al. (2015), Chile [40≠]	Systematic review & meta-	Amiodarone / Placebo; “usual care”	Sub-analysis : heart failure ; NA; NA	RCTs n= 11, n= 5,006	NA	In HF sub-population, amiodarone showed a statistically significant reduction for SCD but not for all-cause mortality. Authors judged the quality of the evidence for the whole	10

For peer review only - http://bmjopen.bmj.com/site/about/guidelines.xhtml

	analysis (Cochrane)					population (primary prevention) as low to moderate and for secondary prevention population as very low.	
Santangeli P. et al. (2012); USA [41]	systematic review	Amiodarone / Placebo	cardiovascular disease; NA; NA	NA	NA	Amiodarone has less favorable net clinical benefits for prophylaxis of SCD because of adverse effects.	5
Piccini JP., et al. (2009); USA [42]	Meta-analysis	Amiodarone / Placebo ; "usual care"	HF, AMI; <45%; II-IV	RCTs n= 15, n= 8,522	Range: 2 to 12	In HF sub-population, amiodarone showed a statistically significant reduction for SCD but not all-cause mortality.	7
ATMA Investigators (1997) [43]	Meta-analysis	Amiodarone / Placebo ; "usual care"	Post-MI and CHF; 31%; NA	NA	Range: 4.8 to 25.8	Amiodarone reduced arrhythmic/sudden death in high-risk patients with recent MI or CHF. All-cause mortality decreased by 13 %.	NA
Sim I. et al., (1997); USA [44]	Meta-analysis	Amiodarone / Placebo ; "usual care"	Subgroup : heart failure; <45%; NA	RCTs n=5, n= 4,125	Range: 6 to 45.6	Amiodarone reduced all-cause mortality in high SCD risk groups.	5
Das MK. et al., (2010): USA [45]	Narrative Review	Anti-arrhythmics / Placebo ; "usual care"	Subgroup : heart failure; NA; NA	NA	NA	Class I Anti-arrhythmic drugs (AADs) increased all-cause mortality and SCD in Post-MI patients. Amiodarone (class III AADs) decreased or have neutral effect on SCD. Caution is warranted to outweigh risks of proarrhythmia and other adverse effects.	NA
Hilleman DE. et al., (2001); USA [46]	Narrative Review	Anti-arrhythmics / Placebo ; "usual care"	HF; <45%; NA	RCTs n= 6 n= 10440	Range: 6.5 to 45	Beta-blockers (Bisoprolol, Carvedilol, Metoprolol) reduced total mortality and SCD in HF. Class I antiarryhthmics increased mortality and SCD in a post hoc analysis of SPAF-I study. Amiodarone had mixed results and dofetilide did not reduce mortality or SCD.	NA
Rizos EC., et al. (2012); Greece [47]	systematic review & meta-analysis	Omega 3 Fatty acids / Placebo ; "usual care"	cardiovascular diseases; NA; NA	RCTs n= 20; n= 68,680	NA	Omega-3 PUFAs supplementation were not associated with a lower risk of all-cause mortality or sudden cardiac death	8
Kotwal S. et al. (2012); Australia [48]	systematic review & meta-analysis	Omega 3 Fatty acids / Placebo ; "usual care"	cardiovascular diseases, heart failure admissions; NA; NA	RCTs n= 20; n= 62,851	Range: 6 to 72	The authors concluded that there is no clear effect on total mortality and sudden death outcomes.	7
Kwak SM., et al. (2012); Korea [49]	Meta-analysis	Omega 3 Fatty acids / Placebo ; "usual care"	secondary prevention of cardiovascular disease; NA; NA	RCTs n=14; n= 20,485	NA	This meta-analysis concluded of insufficient evidence.	8
Chen Q., et al. (2011); China [50]	Meta-analysis	Omega 3 Fatty acids / Placebo ; "usual care"	cardiovascular disease; NA; NA	RCTs n= 10; n= 33,429	NA	Omega-3 fatty acids did not appear to reduce SCD under guideline-adjusted treatment for CVD secondary prevention.	7
Marik PE., et al. (2009); USA [51]	systematic review	Omega 3 dietary supplements / Placebo; olive oil ; corn oil, sunflower oil ; "usual care"	cardiovascular disease; NA; NA	RCTs n= 11; n= 39,044	Range: 12 to 55.2	Dietary supplementation with omega-3 fatty acids reduced SCD and all-cause mortality	4
Wang C., et al. (2006); USA [52]	systematic review	n-3 Fatty acids / Placebo/olive oil ; corn oil, sunflower oil ; "usual care"	Primary and secondary prevention; NA; NA	RCTs n= 12; n= 32,981	Range: 12 to 48	The authors concluded of a significant reduction in all-cause mortality and SCD with n-3 Fatty acids from fish or fish-oil supplements but not α-linolenic acid.	6

≠: studies considered in Table 2 as "reference" studies. *: updated in this study. NA: not applicable

For peer review only - http://bmjopen.bmj.com/site/about/guidelines.xhtml

For peer review only

Risk of bias and quality of reviews

As shown in **Table 1**, the AMSTAR scores for quality assessment of the included reviews widely ranged from 2 to 10 (out of 11). All reviews had one score less because of non-listing of excluded primary studies except Cochrane reviews [26 40] which scored 10 because of non-inclusion of grey literature in the search strategy in one review and missing information for funding resources of included primary studies in another, cited respectively (**S3 Table**). We did not assess the AMSTAR score for six studies, of which two [45 46] were narrative reviews, two[24 43] were individual participant or patient data (IPD) meta-analyses, and the other two [25 31] were overviews of reviews.

The risk of bias of the included primary studies within reviews remained as judged by the original reviews' authors with the exception of the newly added randomized trials in the update of the angiotensin receptor blockers review that we assessed (GRADE) (**Table 2**). The quality of evidence for beta-blockers and anti-aldosterone agents obtained a high quality on the GRADE scale, while angiotensin-converting enzyme inhibitors (ACE-i), amiodarone and statins obtained a moderate quality. However, combined ARB/neprilysin inhibitors had a moderate and high quality for SCD and all-cause mortality outcomes respectively, whereas angiotensin receptor blockers had a low quality of evidence (**Table 2**).

Table 2: Summary of findings and Grade evaluation for sudden cardiac death (SCD) and all-cause mortality prevention

Outcome	Intervention / comparison	Assumed risk with comparator	Corresponding risk with intervention	Relative effect (95% CI)	Number of participants (No of studies)	Quality of the evidence (GRADE)	Comments
**Sudden cardiac death (SCD)**							
	Beta-blockers / placebo	77 per 1000	54 per 1000 (49 to 60)	OR 0.69 (0.62 to 0.77)	24,779 (30 RCTs)	⊕⊕⊕⊕ high*	I^2=0% (P=0.57)
	Anti-aldosterone inhibitor / Placebo ; "usual care"	61 per 1000	49 per 1000 (41 to 60)	RR 0.81 (0.67 to 0.98)	8,301 (5 RCTs)	⊕⊕⊕⊕ high*	I^2=8% (P=0.36)
	ARB;Neprilysin inhibitor/ ACE-i	74 per 1000	60 per 1000 (51 to 70)	RR 0.81 (0.69 to 0.95)	8,399 (1 RCT)	⊕⊕⊕⊖ moderate[e]	

	ACE-i / Placebo	59 per 1000	54 per 1000 (43 to 65)	OR 0.91 (0.73 to 1.11)	6,988 (30 RCT)	⊕⊕⊕⊝ moderate[b]	I^2=0% (P=0.94)
	ARB (or ARB + ACE-i) / Placebo ; ACE-i	93 per 1000	94 per 1000 (76 to 117)	RR 1.01 (0.81 to 1.25)	13,884 (5 RCTs)	⊕⊕⊝⊝ Low[b,c]	I^2=71% (P=0.007)
	Statins / placebo ; "usual care"	108 per 1000	100 per 1000 (76 to 131) [99 per 1000 (72 to 131)]	RR 0.92 (0.7 to 1.21) [OR 0.90 (0.64 to 1.24)]	10,077 (8 RCTs)	⊕⊕⊕⊝ moderate[a]	I^2=42.6% (P=0.094
	Amiodarone / Placebo; "usual care"	118 per 1000	93 per 1000 (79 to 110)	RR 0.79 (0.67 to 0.93)	5,006 (11 RCTs)	⊕⊕⊝⊝ Low[a,b]	
	Omega 3 Fatty acids / Placebo ; "usual care"	93 per 1000	88 per 1000 (77 to 102)	RR 0.94 (0.82 to 1.09)	6,975 (1 RCT)	⊕⊕⊕⊝ moderate[e]	
**All-cause mortality**							
	Beta-blockers / placebo	178 per 1000	127 per 1000 (113 to 141)	OR 0.67 (0.59 to 0.76)	24,779 (26 RCTs)	⊕⊕⊕⊕ high*	I^2= 40% (P=0.02)
	Anti-aldosterone inhibitor / Placebo ; "usual care"	200 per 1000	162 per 1000 (148 to 176)	RR 0.81 (0.74 to 0.88)	9,019 (10 RCTs)	⊕⊕⊕⊕ high*	I^2=0% (P=0.56)
	ARB;Neprilysin inhibitor/ ACE-i	183 per 1000	158 per 1000 (145 to 172)	RR 0.86 (0.79 to 0.94)	14,742 (3 RCTs)	⊕⊕⊕⊝ moderate[b]	I^2=0% (P=0.42)
	ACE-i / Placebo	219 per 1000	178 per 1000 (158 to 198)	OR 0.77 (0.67 to 0.88)	7,105 (32 RCT)	⊕⊕⊕⊝ moderate[b]	I^2=0% (P=0.95)
	ARB (or ARB + ACE-i) / Placebo ; ACE-i	183 per 1000	177 per 1000 (161 to 197)	RR 0.97 (0.88 to 1.08)	19,510 (27 RCTs)	⊕⊕⊝⊝ Low[b,d]	I^2=24% (P=0.14)
	Statins / placebo ; "usual care"	273 per 1000	240 per 1000 (205 to 278) [233 per 1000 (199 to 273)]	RR 0.88 (0.75 to 1.02) OR 0.81 (0.66 to 1)	11,024 (13 RCTs)	⊕⊕⊕⊝ moderate[a]	I^2=37.7% (P=0.083)
	Amiodarone / Placebo ; "usual care"	264 per 1000	237 per 1000 (211 to 266)	RR 0.90 (0.80 to 1.01)	5,006 (11 RCTs)	⊕⊕⊝⊝ Low[a,b]	
	Omega 3 Fatty acids / Placebo ; "usual care"	291 per 1000	274 per 1000 (253 to 294)	RR 0.94 (0.87 to 1.01)	6,975 (1 RCT)	⊕⊕⊕⊝ moderate[e]	

For peer review only

[a] Likelihood of Publication bias presence with an asymmetric funnel plot.

[b] The studies reported to generally have a moderate to high risk of bias due to allocation concealment and blinding reporting.

[c] Inconsistent results ranged from no effect to insignificant increase of events (I^2 = 71%)

[d] Most studies have small sample and wide confidence intervals including no effect with appreciable harm or benefit.

[e] Estimation comes from one single clinical trial. No data obtained from other relevant studies for this outcome

* Although graded high, this might be downgraded into moderate if we strictly consider the risk of bias of primary studies other than an overall estimation.

RR: risk ratio; OR: odds ratio; CI: confidence interval; GRADE: Grading of Recommendations, Assessment, Development and Evaluation; I^2: between-study variance due to heterogeneity.

For peer review only

Up-to-dateness of included reviews

Most retrieved evidence was published within the last 10 years (2008 and on) and 7 (out of 9) drug interventions with updated systematic reviews were within the last 5 years (2012 and on). Moreover, we updated the pooled results for ARBs, which resulted in slightly different results compared to the original Cochrane review [26].

Effectiveness of interventions

We report below the summaries of the effectiveness of the drug interventions considered that we have categorized into effective, ineffective, and uncertain effectiveness (inconclusive or conflicting evidence).

Effective interventions

Beta-blockers (BBs)

Meta-analyses and systematic reviews of randomized controlled trials [14-19] provided overwhelming evidence that beta-blockers (BBs) decrease the risk of SCD and all-cause mortality in HFrEF patients. The quality of the evidence was rated high with a relative effect of 0.69 for SCD [OR, 95% CI (0.62 to 0.77)], and of 0.67 for all-cause mortality [OR, 95% CI (0.59 to 0.76)] (**Table 2**).

Anti-aldosterone agents

Published studies about mineralocorticoid-receptor antagonists (antimineralocorticoids) or (so-called) anti-aldosterones appeared effective in SCD and all-cause mortality prevention [20 21 53]. However, in a recent systematic review [20], adverse effects (hyperkalemia, degradation of renal function, gynecomastia) were significantly higher in the anti-aldosterone-treated group compared to placebo. The quality of the evidence was rated high with relative effect for SCD [RR 0.81, 95% CI (0.67 to 0.98)], and all-cause mortality [RR 0.81, 95% CI (0.74 to 0.88)] (**Table 2**).

Angiotensin receptor blocker (ARB)/neprilysin inhibitor

One meta-analysis [22] estimated the effects of combined neprilysin renin-aldosterone system inhibition and reported a reduction in SCD and all-cause mortality. The finding was principally derived from one RCT (PARADIGM-HF) [4] that showed about 16% reduction of all-cause mortality in favor of sacubitril/valsartan (LCZ696 previously) compared to enalapril (an ACE-i). This mortality reduction was attributed to a decline on both SCD (20%) and pump failure deaths [54]. **Table 2** shows the relative effect for SCD [risk ratio (RR) 0.81, 95% confidence interval (CI) (0.69 to 0.95)], and all-cause mortality [RR 0.86, 95% CI (0.79 to 0.94)]. The moderate quality of the evidence for SCD outcome

For peer review only - http://bmjopen.bmj.com/site/about/guidelines.xhtml

For peer review only

was due to estimation from one single clinical trial and absence of data from other included studies. All-cause mortality was, however, rated as high with a possibility of downgrading in case of a detectable publication bias or unestablished class effect.

Ineffective Interventions

Angiotensin-converting enzyme inhibitors (ACE-i)

Although two systematic reviews [23 24], with an AMSTAR score of 2/11 and 3/11 respectively, reported a decline in total mortality and less progressive HF deaths, SCD events did not significantly decrease [OR 0.91, 95% CI (0.73 to 1.12)]. The quality of the evidence was rated as moderate because of the unclear or high risk of bias in included primary studies (**Table 2**).

Angiotensin receptor blockers (ARBs)

As shown in **Fig 2** and **Fig 3**, we updated a Cochrane review [26] by including more eligible primary studies such as SUPPORT trial [55] comparing ARBs to controls which resulted in slightly different effect size for SCD [RR 1.01, 95% CI (0.81 to 1.25)], and all-cause mortality [RR 0.97, 95% CI (0.88 to 1.08)]. In stratified analyses, ARBs compared to placebo remained ineffective for all-cause mortality [RR 0.79, 95% CI (0.55 to 1.13)]. Similarly, ARBs, compared to ACE-I or in combination versus ACE-i alone, were not superior in all-cause mortality reduction [RR 0.87, 95% CI (0.56 to 1.36); RR 0.99, 95% CI (0.90 to 1.09), respectively] (**Fig 2**). The quality of the evidence is rated as low because of risk of bias, imprecision and inconsistency (I^2 = 71, P=0.007 for SCD outcome) (**Table 2**). Data were limited for studies reporting SCD, in particular those comparing ARBs versus placebo, or versus ACE-i (**Fig 2**). In addition, the funnel plot for all-cause mortality outcome showed no evidence of publication bias (**Fig 4**). No estimation of publication bias and no funnel plot was drawn for SCD as only five studies reported this outcome.

Statins

Published systematic reviews and meta-analyses about statins in HF were inconsistent [5 31-39] with a recent tendency toward inefficacy in total mortality and SCD prevention. The quality of the evidence was rated as moderate because of a likelihood of publication bias revealed on the most up-to-date systematic review [5] (**Table 2**).

Unclear evidence of effectiveness

The evidence of effectiveness of the drug interventions reported below was considered uncertain due either to conflicting or inconclusive evidence.

For peer review only - http://bmjopen.bmj.com/site/about/guidelines.xhtml

Amiodarone & anti-arrhythmic drugs (AADs)

Recently published systematic reviews[40 42] for amiodarone showed a significant reduction for SCD but not for all-cause mortality with less favorable net clinical benefits[41]. Other older reviews [43 44] of minor quality (AMSTAR of 3/11 and 5/11 cited respectively) reported a decline of both SCD and all-cause mortality. The quality of evidence for amiodarone was rated as low because of the unclear or high risk of bias and potential publication bias in primary studies (**Table 2**). No systematic review for AADs of other classes or drugs (other than amiodarone) were retrieved. Nevertheless, two narrative reviews [45 46] reported that class I anti-arrhythmics increased SCD and all-cause mortality. These narrative reviews called for caution regarding the mixed results of amiodarone and its adverse effects.

Omega-3 polyunsaturated fatty acids (PUFA) and fish oil supplementation

No systematic review was exclusively conducted in heart failure patients for this intervention. One primary study[56], known as GISSI-Prevenzione HF, recruited patients with chronic heart failure and reported a lower mortality events in the n-3 polyunsaturated fatty acids (PUFA) group compared to the placebo group. The authors reported an adjusted hazard ratio of 0.91 [95.5% CI 0.833 to 0.998], p=0.041). However, relative risk in our analysis remained statistically insignificant [RR 0.94, 95% CI (0.87 to 1.01), p=0.10] and [RR 0.94, 95% CI (0.81 to 1.09), p=0.42] for all-cause mortality and SCD, respectively. Our assessment of the quality of the evidence involving GISSI-Prevenzione HF was moderate because of an absence of data of any other relevant studies (**Table 2**). In addition, some recent systematic reviews [47-50] included patients regardless of their cardiovascular disease and concluded of no clear effect, insufficient evidence or no reduction on SCD and all-cause mortality outcomes. Meanwhile, some older studies [51 52] reported that omega-3 fatty acids and fish-oil supplements (other than α-linolenic acid [52]) reduced SCD and all-cause mortality.

For peer review only

Discussion

Our assessment of the effectiveness of drug interventions to prevent SCD in HFrEF patients indicated that beta-blockers (BBs), antialdosterone agents, as well as combined angiotensin receptor blocker (ARB)/neprilysin inhibitors were effective.

Previously reported meta-analyses and systematic reviews of RCTs [14-19] indicated that beta-blockers are effective in the prevention of SCD and all-cause mortality in HFrEF. However, although they were increasingly used as a usual “routine” care in the compared arms of the more recently published clinical trials[57], BBs stayed underused for long time and may still be [58]. Mineralocorticoid receptor antagonists or antialdosterone drugs have been reported effective in HFrEF by reducing SCD and all-

For peer review only - http://bmjopen.bmj.com/site/about/guidelines.xhtml

cause mortality [20 21 53 59]. Our summary of finding and consistency of results supports this claim with a high quality of evidence. Only one retrieved meta-analysis [22] supported the effectiveness of combined ARB/neprilysin inhibitor. Although the authors acknowledged the limitation of their meta-analysis, which was not based on a systematic review, but merely pooling three well-known trials published in high impact journals (i.e., IMPRESS [60], OVERTURE [61] and Paradigm-HF [4]). The quality of the evidence is, however, moderate for SCD and high for all-cause mortality, albeit our inability to assess any presence of a class effect or a potential publication bias.

We found that ACE-i showed a total mortality reduction in clinical trials and systematic reviews of heart failure patients [23 24]. However, our overview showed that ACE-i, surprisingly, did not significantly decrease SCD with a moderate quality of evidence.

In addition, we found that neither ARBs nor statins reduced SCD and/or all-cause mortality. Our findings for ARBs were in agreement with Jong and colleagues [30], Shibata et al. [27] and Dimopoulos et al. [29], but in contradiction to Lee VC et al. [28] and Rain & Rada's conclusions [25]. Our up-to-date meta-analysis for ARBs included only five primary studies, but large-scale trials, that reported SCD events. The pooled estimate showed inefficacy of ARBs compared to control in the prevention of SCD [RR 1.01, 95% CI (0.81 to 1.25). Moreover, the addition of current trials such as SUPPORT [55], improved the statistical power of detecting an effect if existed and the summary statistic moved from a borderline effect to an insignificant result (**Fig 2**). Of note, Jong and colleagues [30] attributed this inefficacy of ARBs in HF to the background treatment with ACE-i.

Within the current evidence, ARBs should not be seen as interchangeable with ACE-i, which also showed a neutral effect on SCD, without a proper reason. Therefore, in a high-risk SCD patient, another therapeutic strategy should be sought and an ARB/neprilysin inhibitor might be an alternative in patients similar to those of the Paradigm-HF trial[4].

The addition of statins to the therapy regimen of heart failure patients had no survival benefits. Actually, a recent systematic review and meta-analysis indicated that statins did not reduce SCD nor all-cause mortality [5]. Our current study reached the same conclusion with similar quality of evidence.

Our overview showed unclear evidence of effectiveness of Omega-3 polyunsaturated fatty acids (PUFA), fish oil supplementation and AADs. The latter intervention had an evidence originated from only narrative reviews, as we did not identify any systematic reviews. Also, only one n-3 PUFA clinical trial [56] was conducted in HF patients and reported a statistically significant mortality reduction; this result was not supported by other trials and recent systematic reviews [47-50] , a finding that justified our

For peer review only - http://bmjopen.bmj.com/site/about/guidelines.xhtml

conclusion of unclear evidence. Moreover, no other data or systematic reviews conducted in HF were retrieved by our electronic and manual searches.

AADs are classified into four categories [45]: sodium channel blocking drugs (class I), BBs (class II), potassium channel blockers (class III), and calcium channel blockers (class IV). We found inconclusive evidence of effectiveness of all categories, with the exception of BBs. The evidence of effectiveness of class I, III and IV is inconclusive, neutral or even detrimental to patients as for class I AADs [45 46]. Amiodarones, which present class I, II, III, and IV effects, reported mixed results with potential SCD prevention with adverse effects [42] and potentially, but rare [62], life-threatening proarrhythmias [45].

Our overview has some limitations. First, we limited the scope of our study to drug treatment, thus excluding devices like implantable cardioverter defibrillators. We believe that non-drug devices should be tackled in future research. ESC Guideline (2016)[63] and others (e.g. www.uptodate.com) recommend the use of ICDs for only ≤ 35% HF patients and only after optimization of drug therapy. In fact, SCDs occur in both reduced and preserved heart failure. Our overview might help to optimize therapy as a first step before introducing ICDs, which applies to a limited HF subpopulation, regardless of costs. Second, we may have failed to include other drug interventions used in HFrEF. Such drug candidates include digoxin, I_f-channel blockers (ivabradine), hydralazine/isosorbide dinitrate, nitroglycerin, and phosphodiesterase 3 or 5 inhibitors. However, our overview included most commonly prescribed and evidence-based pharmacological therapy in HF as pre-specified in our published protocol[10]. Third, we did not use specific drug names in our literature search strategy, in order to avoid omitting a therapy that evaluated SCD and/or all-cause mortality prevention in heart failure patients. Fourth, we based our analyses on existing systematic reviews and meta-analyses and we updated only one meta-analysis. Consequently, we were unable to update the evidence for ACE-inhibitors. Furthermore, as indicated by the AMSTAR score, the methodological quality of some of the existing reviews was suboptimal. Fifth, we did not assess the safety of the evaluated drug interventions, nor the contra-indications for their prescription, drug-drug interactions, as well as treatment adherence. Indeed, we considered that these important aspects were out of the scope of our analysis. Sixth, we were unable to do a sensitivity analysis, initially suggested in our protocol, for ischaemic versus non-ischaemic heart failure due to limited data availability. Finally, a potential source of bias relates to authors of this overview being the authors of three of the included reviews [15 20 64]. However, the adopted methodology is in line with systematic reviews guidelines and ensured a

For peer review only - http://bmjopen.bmj.com/site/about/guidelines.xhtml

double check of data and methodological evaluation by at least two reviewers and a published protocol [10].

It is noteworthy that high quality evidence does not necessarily imply strong recommendations, and strong recommendations can arise from low quality evidence [65]. Therefore, when one intervention is graded high, it is not our intention to say that it is highly recommended, as we did not assess the level of recommendation in our study. In fact, level of recommendation not only depends on the strength of evidence, but also (among others) on values and preferences of patients, net benefits and cost-effectiveness of a particular intervention.

Implications for practice

Our study summarizes and synthesizes the effectiveness of most evidence-based drug interventions in HF patients with reduced ejection fraction (HFrEF) for SCD prevention. It classified drug interventions according to the current evidence of their effectiveness. This categorization could help health professionals and patients making evidence-based decisions based on updated knowledge, particularly whenever a high-risk SCD patient is identified. Currently, there is no an established strategy to deal with patients at high risk of SCD. In such patients, a particular attention should be considered and a careful selection of available therapeutic options is needed. Furthermore, there might be a shift towards an alternative therapeutic strategy based on SCD prevention-effective drugs in light of our findings.

Conclusion

Our overview indicates that only three drug interventions (BBs, anti-aldosterones, combined ARB/neprilysin inhibitors) significantly reduce SCD and improve overall survival among individuals with heart failure and reduced ejection fraction. On the other hand, there is no evidence of effectivness of ARBs to reduce neither all-cause mortality nor SCD, and ACE-i do not significantly reduce SCD events. When the goal of drug therapy is to reduce SCD, especially in high-risk patients, our synthesis supports the use of the most effective regimen.

Author contribution

Design and conception: MA, FG, BB. (MA is the guarantor). Project administration: BB. Writing original draft: MA. Critical analysis: MA, SA, FG, BB. Data curation: MA, SA. Statistical analysis: MA. Proofread and approved the final draft: MA, SA, FG, BB.

For peer review only - http://bmjopen.bmj.com/site/about/guidelines.xhtml

For peer review only

Competing interests

We declare that some authors of this overview are also authors of some of the included reviews. However, at least two reviewers systematically checked and validated the extracted data including study qualities. We declare that no other competing interests exist.

Funding

MA, the corresponding author, received a financial grant (2014.0193) from The Federal Department of Economic Affairs, Education and Research (EAER), Switzerland, also from Département universitaire de médecine et santé communautaires (DUMSC), Lausanne university hospital (CHUV), Switzerland. The former funders had no role in study design or conception, study analysis, writing or preparation of the manuscript.

Ethics committee approval

This study did not need to ask for ethical approval and no direct patient involvement occurred.

Data sharing

All data are available in the manuscript and its supported files. Any more information can be requested from the corresponding author.

Acknowledgements

We thank Emilie Zuercher for her technical assistance.

Supporting materials

- S1 File: PRISMA checklist
- S2 File: Search strategy equations and result
- S3 Table: AMSTAR scores.
- S4 Table: Funding data of included reviews

Abbreviations

- ACE-i: angiotensin-converting enzyme inhibitor
- ACS: acute coronary syndrome
- ARBs: angiotensin receptor blockers

For peer review only

- ATMA: amiodarone trial meta-analysis
- BBs: beta-blockers
- CHD: coronary heart disease
- CHF: chronic heart disease
- GRADE: Grading of Recommendations, Assessment, Development and Evaluation
- HF: heart failure
- HFrEF: heart failure with reduced ejection fraction
- LVD: left ventricular dysfunction
- MI: Myocardial Infarction
- NA: not available or not applicable.
- NR: not reported
- NYHA: New York Heart Association class
- Post-MI: post myocardial infarction
- PUFAs: polyunsaturated fatty acids
- RAS: renin angiontensin system
- RCTs: randomized controlled trials
- SCD: sudden cardiac death
- SPAF-I: Stroke prevention Atrial Fibrillation Study

References

1. Thom T, Haase N, Rosamond W, et al. Heart disease and stroke statistics--2006 update: a report from the American Heart Association Statistics Committee and Stroke Statistics Subcommittee. *Circulation* 2006;113(6):e85-151. doi: 10.1161/circulationaha.105.171600 [published Online First: 2006/01/13]
2. Tomaselli GF, Zipes DP. What causes sudden death in heart failure? *Circulation research* 2004;95(8):754-63. doi: 10.1161/01.RES.0000145047.14691.db [published Online First: 2004/10/16]
3. Ponikowski P, Voors AA, Anker SD, et al. 2016 ESC Guidelines for the diagnosis and treatment of acute and chronic heart failure: The Task Force for the diagnosis and treatment of acute and chronic heart failure of the European Society of Cardiology (ESC)Developed with the special contribution of the Heart Failure Association (HFA) of the ESC. *European heart journal* 2016;37(27):2129-200. doi: 10.1093/eurheartj/ehw128 [published Online First: 2016/05/22]
4. McMurray JJ, Packer M, Desai AS, et al. Angiotensin-neprilysin inhibition versus enalapril in heart failure. *N Engl J Med* 2014;371(11):993-1004. doi: 10.1056/NEJMoa1409077 [published Online First: 2014/09/02]
5. Al-Gobari M, Le HH, Fall M, et al. No benefits of statins for sudden cardiac death prevention in patients with heart failure and reduced ejection fraction: A meta-analysis of randomized controlled trials. *Plos One* 2017;12(2)
6. Lee E, Dobbins M, Decorby K, et al. An optimal search filter for retrieving systematic reviews and meta-analyses. *BMC medical research methodology* 2012;12:51. doi: 10.1186/1471-2288-12-51 [published Online First: 2012/04/20]
7. Wilczynski NL, Haynes RB. EMBASE search strategies achieved high sensitivity and specificity for retrieving methodologically sound systematic reviews. *Journal of clinical epidemiology* 2007;60(1):29-33. doi: 10.1016/j.jclinepi.2006.04.001 [published Online First: 2006/12/13]
8. Ouzzani M, Hammady H, Fedorowicz Z, et al. Rayyan-a web and mobile app for systematic reviews. *Systematic reviews* 2016;5(1):210. doi: 10.1186/s13643-016-0384-4 [published Online First: 2016/12/07]

For peer review only - http://bmjopen.bmj.com/site/about/guidelines.xhtml

9. Shea BJ, Grimshaw JM, Wells GA, et al. Development of AMSTAR: a measurement tool to assess the methodological quality of systematic reviews. *BMC medical research methodology* 2007;7:10. doi: 10.1186/1471-2288-7-10 [published Online First: 2007/02/17]
10. Al-Gobari M A-AS, Gueyffier F, Burnand B. Effectiveness of drug interventions to prevent sudden cardiac death in patients with heart failure and reduced ejection fraction: an overview of systematic reviews. PROSPERO 2017:CRD42017067442 2017; Available from http://www.crd.york.ac.uk/PROSPERO/display_record.asp?ID=CRD42017067442. 2017
11. Kunz R, Burnand B, Schünemann HJ. Das GRADE-System. *Der Internist* 2008;49(6):673-80. doi: 10.1007/s00108-008-2141-9
12. Mantel N, Haenszel W. Statistical aspects of the analysis of data from retrospective studies of disease. *Journal of the National Cancer Institute* 1959;22(4):719-48. [published Online First: 1959/04/01]
13. Higgins JP, Thompson SG, Deeks JJ, et al. Measuring inconsistency in meta-analyses. *BMJ (Clinical research ed)* 2003;327(7414):557-60. doi: 10.1136/bmj.327.7414.557 [published Online First: 2003/09/06]
14. Chatterjee S, Biondi-Zoccai G, Abbate A, et al. Benefits of beta blockers in patients with heart failure and reduced ejection fraction: network meta-analysis. *BMJ (Clinical research ed)* 2013;346:no pagination.
15. Al-Gobari M, El Khatib C, Pillon F, et al. Beta-blockers for the prevention of sudden cardiac death in heart failure patients: a meta-analysis of randomized controlled trials. *Bmc Cardiovascular Disorders* 2013;13
16. Brophy JM, Joseph L, Rouleau JL. Beta-blockers in congestive heart failure. A Bayesian meta-analysis. *Annals of internal medicine* 2001;134(7):550-60. [published Online First: 2001/04/03]
17. Lee S, Spencer A. Beta-blockers to reduce mortality in patients with systolic dysfunction: A meta-analysis. *Journal of Family Practice* 2001;50(6):499-504.
18. Bonet S, Agusti A, Arnau JM, et al. Beta-adrenergic blocking agents in heart failure: benefits of vasodilating and nonvasodilating agents according to patients' characteristics: a meta-analysis of clinical trials (Structured abstract). *Archives of Internal Medicine* 2000;160(5):621-27.
19. Heidenreich PA, Lee TT, Massie BM. Effect of beta-blockade on mortality in patients with heart failure: A meta-analysis of randomized clinical trials. *Journal of the American College of Cardiology* 1997;30(1):27-34.
20. Le HH, El-Khatib C, Mombled M, et al. Impact of Aldosterone Antagonists on Sudden Cardiac Death Prevention in Heart Failure and Post-Myocardial Infarction Patients: A Systematic Review and Meta-Analysis of Randomized Controlled Trials. *Plos One* 2016;11(2)
21. Wei J, Ni J, Huang D, et al. The effect of aldosterone antagonists for ventricular arrhythmia: a meta-analysis. *Clinical cardiology* 2010;33(9):572-7.
22. Solomon SD, Claggett B, McMurray JJ, et al. Combined neprilysin and renin-angiotensin system inhibition in heart failure with reduced ejection fraction: a meta-analysis. *European journal of heart failure* 2016;18(10):1238-43. doi: 10.1002/ejhf.603 [published Online First: 2016/07/02]
23. Garg R, Yusuf S. Overview of randomized trials of angiotensin-converting enzyme inhibitors on mortality and morbidity in patients with heart failure. Collaborative Group on ACE Inhibitor Trials. *Jama* 1995;273(18):1450-6. [published Online First: 1995/05/10]
24. Flather MD, Yusuf S, Kober L, et al. Long-term ACE-inhibitor therapy in patients with heart failure or left-ventricular dysfunction: a systematic overview of data from individual patients. ACE-Inhibitor Myocardial Infarction Collaborative Group. *Lancet* 2000;355(9215):1575-81. [published Online First: 2000/05/23]
25. Rain C, Rada G. Is there any difference between angiotensin converting enzyme inhibitors and angiotensin receptor blockers for heart failure? *Medwave* 2015;15 Suppl 1:e6177. doi: 10.5867/medwave.2015.06.6177 [published Online First: 2015/08/08]
26. Heran BS, Musini VM, Bassett K, et al. Angiotensin receptor blockers for heart failure. *The Cochrane database of systematic reviews* 2012(4):Cd003040. doi: 10.1002/14651858.CD003040.pub2 [published Online First: 2012/04/20]
27. Shibata MC, Tsuyuki RT, Wiebe N. The effects of angiotensin-receptor blockers on mortality and morbidity in heart failure: a systematic review. *International journal of clinical practice* 2008;62(9):1397-402. doi: 10.1111/j.1742-1241.2008.01806.x [published Online First: 2008/09/17]

For peer review only - http://bmjopen.bmj.com/site/about/guidelines.xhtml

28. Lee VC, Rhew DC, Dylan M, et al. Meta-analysis: angiotensin-receptor blockers in chronic heart failure and high-risk acute myocardial infarction. *Annals of internal medicine* 2004;141(9):693-704. [published Online First: 2004/11/03]
29. Dimopoulos K, Salukhe TV, Coats AJ, et al. Meta-analyses of mortality and morbidity effects of an angiotensin receptor blocker in patients with chronic heart failure already receiving an ACE inhibitor (alone or with a beta-blocker). *International journal of cardiology* 2004;93(2-3):105-11. doi: 10.1016/j.ijcard.2003.10.001 [published Online First: 2004/02/21]
30. Jong P, Demers C, McKelvie RS, et al. Angiotensin receptor blockers in heart failure: meta-analysis of randomized controlled trials. *J Am Coll Cardiol* 2002;39(3):463-70. [published Online First: 2002/02/02]
31. Rain C, Rada G. Are statins beneficial for chronic heart failure? First update. *Medwave* 2017;17(Suppl2):e6853. doi: 10.5867/medwave.2017.6853 [published Online First: 2017/04/30]
32. Osei Bonsu K, Reidpath DD, Kadirvelu A. Hydrophilic versus lipophilic statin therapy on outcomes in patients with heart failure: A systematic review and an adjusted indirect comparison meta-analysis of randomised trials. *European heart journal* 2015;36:546.
33. Wang JQ, Wu GR, Wang Z, et al. Long-term Clinical Outcomes of Statin Use for Chronic Heart Failure: A Meta-analysis of 15 Prospective Studies. *Heart Lung and Circulation* 2014;23(2):105-13.
34. Liu G, Zheng XX, Xu YL, et al. Effects of lipophilic statins for heart failure: a meta-analysis of 13 randomised controlled trials. *Heart, lung & circulation* 2014;23(10):970-7. doi: 10.1016/j.hlc.2014.05.005 [published Online First: 2014/06/24]
35. Zhang S, Zhang L, Sun A, et al. Efficacy of statin therapy in chronic systolic cardiac insufficiency: a meta-analysis. *European journal of internal medicine* 2011;22(5):478-84. doi: 10.1016/j.ejim.2011.06.003 [published Online First: 2011/09/20]
36. Xu M, Yuan G, Wei F. Effect of atorvastatin in patients with chronic heart failure - insights from randomized clinical trials. *Archives of medical science : AMS* 2010;6(6):866-73.
37. Lipinski MJ, Cauthen CA, Biondi-Zoccai GG, et al. Meta-analysis of randomized controlled trials of statins versus placebo in patients with heart failure. *The American journal of cardiology* 2009;104(12):1708-16. doi: 10.1016/j.amjcard.2009.07.055 [published Online First: 2009/12/08]
38. Levantesi G, Scarano M, Marfisi R, et al. Meta-analysis of effect of statin treatment on risk of sudden death. *The American journal of cardiology* 2007;100(11):1644-50. doi: 10.1016/j.amjcard.2007.07.015 [published Online First: 2007/11/27]
39. Rahimi K, Majoni W, Merhi A, et al. Effect of statins on ventricular tachyarrhythmia, cardiac arrest, and sudden cardiac death: A meta-analysis of published and unpublished evidence from randomized trials. *European heart journal* 2012;33(13):1571-81.
40. Claro JC, Candia R, Rada G, et al. Amiodarone versus other pharmacological interventions for prevention of sudden cardiac death. *Cochrane Database of Systematic Reviews* 2015;0(12)
41. Santangeli P, Di Biase L, Burkhardt JD, et al. Examining the safety of amiodarone. *Expert Opinion on Drug Safety* 2012;11(2):191-214.
42. Piccini JP, Berger JS, O'Connor CM. Amiodarone for the prevention of sudden cardiac death: a meta-analysis of randomized controlled trials. *European heart journal* 2009;30(10):1245-53.
43. Effect of prophylactic amiodarone on mortality after acute myocardial infarction and in congestive heart failure: meta-analysis of individual data from 6500 patients in randomised trials (Structured abstract). *Lancet* 1997;350(2):1417-24.
44. Sim I, McDonald KM, Lavori PW, et al. Quantitative overview of randomized trials of amiodarone to prevent sudden cardiac death. *Circulation* 1997;96(9):2823-29.
45. Das MK, Zipes DP. Antiarrhythmic and Nonantiarrhythmic Drugs for Sudden Cardiac Death Prevention. *Journal of Cardiovascular Pharmacology* 2010;55(5):438-49.
46. Hilleman DE, Bauman AL. Role of antiarrhythmic therapy in patients at risk for sudden cardiac death: an evidence-based review. *Pharmacotherapy* 2001;21(5):556-75. [published Online First: 2001/05/15]
47. Rizos E, Ntzani E, Bika E, et al. Association between omega-3 fatty acid supplementation and risk of major cardiovascular disease events: a systematic review and meta-analysis (Structured abstract). *Jama* 2012;308(10):1024-33.

For peer review only - http://bmjopen.bmj.com/site/about/guidelines.xhtml

48. Kotwal S, Jun M, Sullivan D, et al. Omega 3 Fatty acids and cardiovascular outcomes: systematic review and meta-analysis. *Circulation Cardiovascular quality and outcomes* 2012;5(6):808-18. doi: 10.1161/circoutcomes.112.966168 [published Online First: 2012/11/01]
49. Kwak SM, Myung SK, Lee YJ, et al. Efficacy of omega-3 fatty acid supplements (eicosapentaenoic acid and docosahexaenoic acid) in the secondary prevention of cardiovascular disease: A meta-analysis of randomized, double-blind, placebo-controlled trials. *Archives of Internal Medicine* 2012;172(9):686-94.
50. Chen Q, Cheng L, Xiao T, et al. Effects of omega-3 fatty acid for sudden cardiac death prevention in patients with cardiovascular disease: a contemporary meta-analysis of randomized, controlled trials (Structured abstract). *Cardiovascular Drugs and Therapy* 2011;25(3):259-65.
51. Marik PE, Varon J. Omega-3 dietary supplements and the risk of cardiovascular events: a systematic review. *Clinical cardiology* 2009;32(7):365-72.
52. Wang C, Harris WS, Chung M, et al. n-3 Fatty acids from fish or fish-oil supplements, but not alpha-linolenic acid, benefit cardiovascular disease outcomes in primary- and secondary-prevention studies: a systematic review. *The American journal of clinical nutrition* 2006;84(1):5-17. [published Online First: 2006/07/11]
53. Bapoje SR, Bahia A, Hokanson JE, et al. Effects of Mineralocorticoid Receptor Antagonists on the Risk of Sudden Cardiac Death in Patients With Left Ventricular Systolic Dysfunction A Meta-analysis of Randomized Controlled Trials. *Circulation-Heart Failure* 2013;6(2):166-+.
54. Desai AS, McMurray JJ, Packer M, et al. Effect of the angiotensin-receptor-neprilysin inhibitor LCZ696 compared with enalapril on mode of death in heart failure patients. *European heart journal* 2015;36(30):1990-7. doi: 10.1093/eurheartj/ehv186 [published Online First: 2015/05/30]
55. Sakata Y, Shiba N, Takahashi J, et al. Clinical impacts of additive use of olmesartan in hypertensive patients with chronic heart failure: the supplemental benefit of an angiotensin receptor blocker in hypertensive patients with stable heart failure using olmesartan (SUPPORT) trial. *European heart journal* 2015;36(15):915-23. doi: 10.1093/eurheartj/ehu504 [published Online First: 2015/02/02]
56. Tavazzi L, Maggioni AP, Marchioli R, et al. Effect of n-3 polyunsaturated fatty acids in patients with chronic heart failure (the GISSI-HF trial): a randomised, double-blind, placebo-controlled trial. *Lancet* 2008;372(9645):1223-30. doi: 10.1016/s0140-6736(08)61239-8 [published Online First: 2008/09/02]
57. Shen L, Jhund PS, Petrie MC, et al. Declining Risk of Sudden Death in Heart Failure. *The New England journal of medicine* 2017;377(1):41-51. doi: 10.1056/NEJMoa1609758 [published Online First: 2017/07/06]
58. Busson A, Thilly N, Laborde-Casterot H, et al. Effectiveness of guideline-consistent heart failure drug prescriptions at hospital discharge on 1-year mortality: Results from the EPICAL2 cohort study. *European journal of internal medicine* 2018 doi: 10.1016/j.ejim.2017.12.005 [published Online First: 2018/01/07]
59. Anand K, Mooss AN, Mohiuddin SM. Aldosterone inhibition reduces the risk of sudden cardiac death in patients with heart failure. *Journal of the Renin-Angiotensin-Aldosterone System* 2006;7(1):15-19.
60. Rouleau JL, Pfeffer MA, Stewart DJ, et al. Comparison of vasopeptidase inhibitor, omapatrilat, and lisinopril on exercise tolerance and morbidity in patients with heart failure: IMPRESS randomised trial. *Lancet* 2000;356(9230):615-20. [published Online First: 2000/09/01]
61. Packer M, Califf RM, Konstam MA, et al. Comparison of omapatrilat and enalapril in patients with chronic heart failure: the Omapatrilat Versus Enalapril Randomized Trial of Utility in Reducing Events (OVERTURE). *Circulation* 2002;106(8):920-6. [published Online First: 2002/08/21]
62. Doggrell SA. Amiodarone - Waxed and waned and waxed again. *Expert Opinion on Pharmacotherapy* 2001;2(11):1877-90.
63. Ponikowski P, Voors AA, Anker SD, et al. 2016 ESC Guidelines for the diagnosis and treatment of acute and chronic heart failure: The Task Force for the diagnosis and treatment of acute and chronic heart failure of the European Society of Cardiology (ESC). Developed with the special contribution of the Heart Failure Association (HFA) of the ESC. *European journal of heart failure* 2016;18(8):891-975. doi: 10.1002/ejhf.592 [published Online First: 2016/05/22]
64. Al-Gobari M, Le HH, Fall M, et al. No benefits of statins for sudden cardiac death prevention in patients with heart failure and reduced ejection fraction: A meta-analysis of randomized controlled trials. *PloS one* 2017;12(2):e0171168. doi: 10.1371/journal.pone.0171168 [published Online First: 2017/02/07]

For peer review only - http://bmjopen.bmj.com/site/about/guidelines.xhtml

65. Guyatt GH, Oxman AD, Vist GE, et al. GRADE: an emerging consensus on rating quality of evidence and strength of recommendations. *BMJ : British Medical Journal* 2008;336(7650):924-26. doi: 10.1136/bmj.39489.470347.AD

Legends of figures

Fig 1: flow chart for search result

Fig 2: Efficacy of angiotensin receptor blockers (ARBs) compared with placebo**,** angiotensin-converting enzyme inhibitor (ACE-i), or combined in heart failure and reduced ejection fraction (HFrEF) for the prevention of **all-cause mortality**.

Fig 3: Efficacy of angiotensin receptor blockers (ARBs) compared with placebo**,** angiotensin-converting enzyme inhibitor (ACE-i), or combined in heart failure and reduced ejection fraction (HFrEF) for the prevention of **sudden cardiac death (SCD).**

Fig 4: Funnel plot of SE (log odds ratio) by odds ratio to evaluate publication bias for the efficacy of angiotensin receptor blockers (ARBs) compared with control in heart failure and reduced ejection fraction (HFrEF) for the prevention of **all-cause mortality.**

For peer review only

For peer review only - http://bmjopen.bmj.com/site/about/guidelines.xhtml

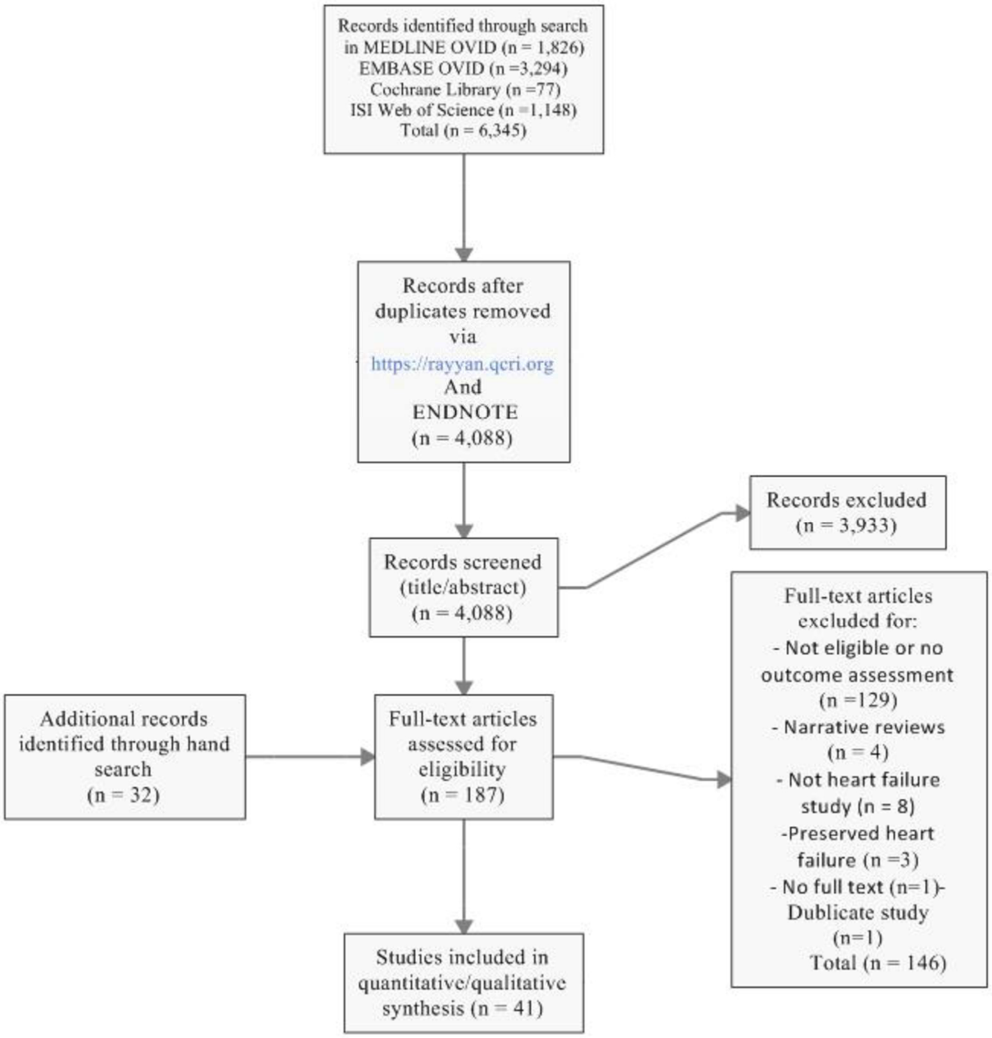

Fig 1: flow chart for search result.

160x167mm (300 x 300 DPI)

Study or Subgroup	ARBs Events	ARBs Total	Control Events	Control Total	Weight	Odds Ratio M-H, Fixed, 95% CI
**1.1.1 ARBs vs Placebo**						
ARCH-J 2003	2	148	3	144	0.2%	0.64 [0.11, 3.91]
CHARM-Alternative 2003	265	1013	296	1015	17.3%	0.86 [0.71, 1.05]
Crozier 1995	4	125	0	29	0.1%	2.19 [0.11, 41.72]
Mazayev 1998	1	75	1	26	0.1%	0.34 [0.02, 5.60]
Mitrovic 2003	5	174	2	44	0.2%	0.62 [0.12, 3.31]
Sharma 2000, III-In'l	3	254	9	131	0.9%	0.16 [0.04, 0.61]
Sharma 2000, III-US	4	237	4	114	0.4%	0.47 [0.12, 1.92]
SPICE 2000	6	179	3	91	0.3%	1.02 [0.25, 4.16]
STRECH 1999	10	633	1	211	0.1%	3.37 [0.43, 26.49]
Weber 1997	4	125	0	29	0.1%	2.19 [0.11, 41.72]
**Subtotal (95% CI)**		**2963**		**1834**	**19.8%**	**0.84 [0.70, 1.00]**
Total events	304		319			
Heterogeneity: Chi² = 9.87, df = 9 (P = 0.36); I² = 9%						
Test for overall effect: Z = 1.91 (P = 0.06)						
**1.1.2 ARBs vs ACE-i**						
Dickstein 1995	2	108	2	58	0.2%	0.53 [0.07, 3.85]
ELITE 1997	17	352	32	370	2.4%	0.54 [0.29, 0.98]
ELITE II 2000	280	1578	250	1574	16.3%	1.14 [0.95, 1.38]
HEAVEN 2002	1	70	5	71	0.4%	0.19 [0.02, 1.68]
Lang 1997	6	78	0	38	0.0%	6.90 [0.38, 125.83]
Mazayev 1998	1	75	0	15	0.1%	0.62 [0.02, 16.05]
REPLACE 2001	4	301	2	77	0.2%	0.51 [0.09, 2.81]
RESOLVD 1999	20	327	4	109	0.4%	1.71 [0.57, 5.12]
**Subtotal (95% CI)**		**2889**		**2312**	**20.1%**	**1.06 [0.90, 1.26]**
Total events	331		295			
Heterogeneity: Chi² = 11.47, df = 7 (P = 0.12); I² = 39%						
Test for overall effect: Z = 0.71 (P = 0.48)						
**1.1.3 ARBs+ACE-i vs ACE-i alone**						
ADEPT 2001	0	18	0	18		Not estimable
CHARM-Added 2003	377	1276	412	1272	23.0%	0.88 [0.74, 1.04]
Hamroff 1999	0	16	1	17	0.1%	0.33 [0.01, 8.79]
RESOLVD 1999	29	332	4	109	0.4%	2.51 [0.86, 7.31]
SUPPORT 2015	98	578	85	568	5.6%	1.16 [0.85, 1.59]
Tonkon 2000	0	57	0	52		Not estimable
V-HeFT 1999	2	55	0	28	0.0%	2.66 [0.12, 57.39]
Val-HeFT 2001	495	2511	484	2499	30.8%	1.02 [0.89, 1.18]
Yasumura 2004	1	49	1	57	0.1%	1.17 [0.07, 19.16]
**Subtotal (95% CI)**		**4892**		**4620**	**60.2%**	**0.99 [0.90, 1.09]**
Total events	1002		987			
Heterogeneity: Chi² = 6.98, df = 6 (P = 0.32); I² = 14%						
Test for overall effect: Z = 0.20 (P = 0.84)						
**Total (95% CI)**		**10744**		**8766**	**100.0%**	**0.97 [0.90, 1.05]**
Total events	1637		1601			
Heterogeneity: Chi² = 32.09, df = 24 (P = 0.12); I² = 25%						
Test for overall effect: Z = 0.65 (P = 0.52)						
Test for subgroup differences: Chi² = 3.77, df = 2 (P = 0.15), I² = 47.0%						

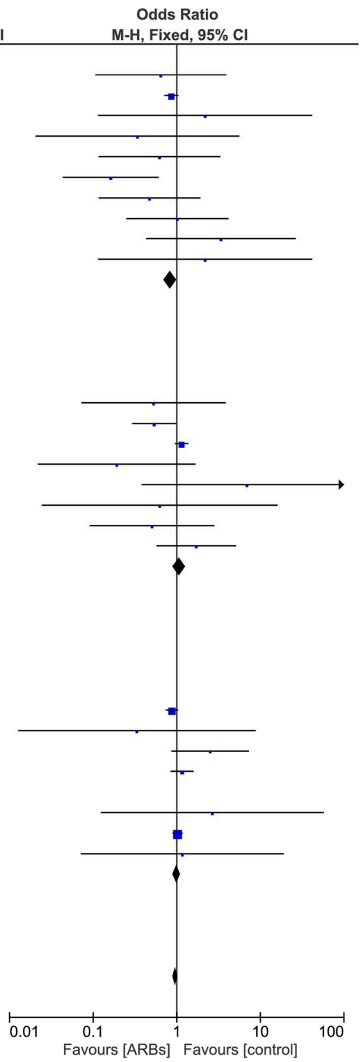

Fig 2: Efficacy of angiotensin receptor blockers (ARBs) compared with placebo, angiotensin-converting enzyme inhibitor (ACE-i), or combined in heart failure and reduced ejection fraction (HFrEF) for the prevention of all-cause mortality.

190x201mm (300 x 300 DPI)

Study or Subgroup	Experimental Events	Experimental Total	Control Events	Control Total	Weight	Risk Ratio M-H, Random, 95% CI
**1.15.1 ARB versus placebo**						
CHARM-Alternative 2003	80	1013	111	1015	21.0%	0.72 [0.55, 0.95]
**Subtotal (95% CI)**		**1013**		**1015**	**21.0%**	**0.72 [0.55, 0.95]**
Total events	80		111			
Heterogeneity: Not applicable						
Test for overall effect: Z = 2.33 (P = 0.02)						
**1.15.2 ARB versus ACE-i**						
ELITE II 2000	130	1578	101	1574	22.2%	1.28 [1.00, 1.65]
**Subtotal (95% CI)**		**1578**		**1574**	**22.2%**	**1.28 [1.00, 1.65]**
Total events	130		101			
Heterogeneity: Not applicable						
Test for overall effect: Z = 1.96 (P = 0.05)						
**1.15.3 ARB + ACE-i versus ACE-i alone**						
CHARM-Added 2003	150	1276	168	1272	24.5%	0.89 [0.72, 1.09]
SUPPORT 2015	18	578	8	568	5.7%	2.21 [0.97, 5.04]
Val-HeFT 2001	262	2511	258	2499	26.7%	1.01 [0.86, 1.19]
**Subtotal (95% CI)**		**4365**		**4339**	**56.9%**	**1.01 [0.80, 1.28]**
Total events	430		434			
Heterogeneity: Tau² = 0.02; Chi² = 4.72, df = 2 (P = 0.09); I² = 58%						
Test for overall effect: Z = 0.11 (P = 0.92)						
**Total (95% CI)**		**6956**		**6928**	**100.0%**	**1.01 [0.81, 1.25]**
Total events	640		646			
Heterogeneity: Tau² = 0.04; Chi² = 13.96, df = 4 (P = 0.007); I² = 71%						
Test for overall effect: Z = 0.06 (P = 0.95)						
Test for subgroup differences: Chi² = 9.26, df = 2 (P = 0.010), I² = 78.4%						

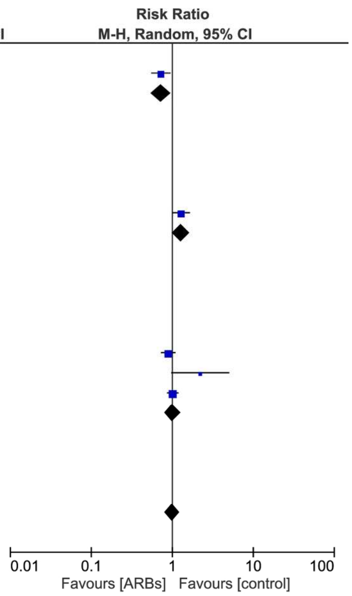

Fig 3: Efficacy of angiotensin receptor blockers (ARBs) compared with placebo, angiotensin-converting enzyme inhibitor (ACE-i), or combined in heart failure and reduced ejection fraction (HFrEF) for the prevention of sudden cardiac death (SCD).

190x113mm (300 x 300 DPI)

view only

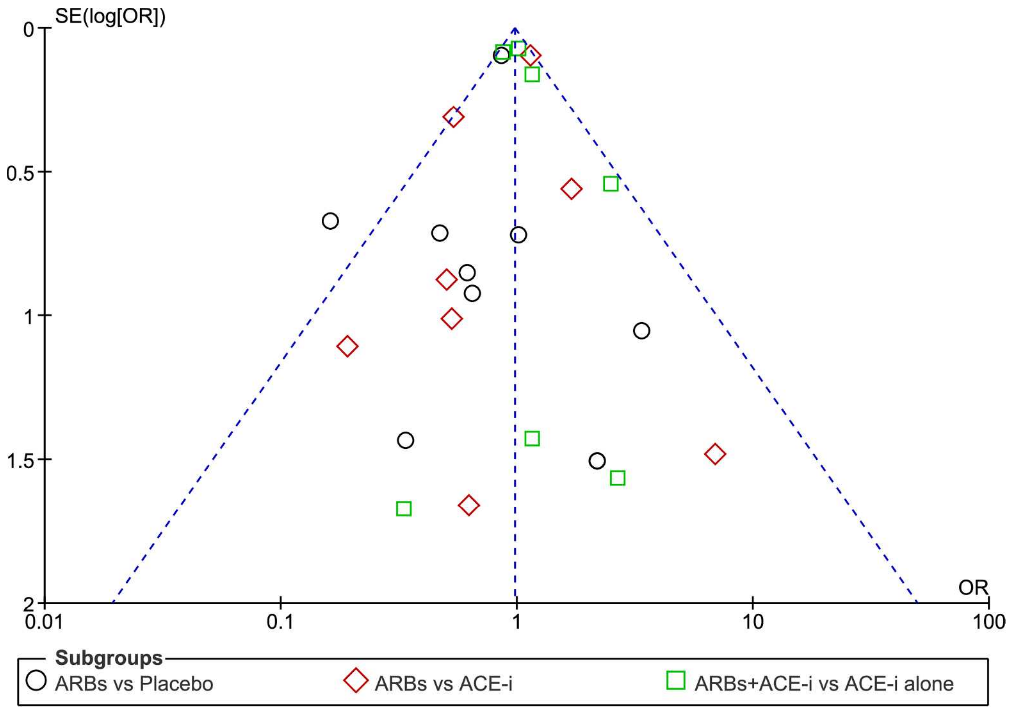

Fig 4: Funnel plot of SE (log odds ratio) by odds ratio to evaluate publication bias for the efficacy of angiotensin receptor blockers (ARBs) compared with control in heart failure and reduced ejection fraction (HFrEF) for the prevention of all-cause mortality.

190x133mm (300 x 300 DPI)

S1 File: PRISMA Checklist

Section/topic	#	Checklist item	Reported on page #
**TITLE**			
Title	1	Identify the report as a systematic review, meta-analysis, or both.	1
**ABSTRACT**			
Structured summary	2	Provide a structured summary including, as applicable: background; objectives; data sources; study eligibility criteria, participants, and interventions; study appraisal and synthesis methods; results; limitations; conclusions and implications of key findings; systematic review registration number.	2
**INTRODUCTION**			
Rationale	3	Describe the rationale for the review in the context of what is already known.	4
Objectives	4	Provide an explicit statement of questions being addressed with reference to participants, interventions, comparisons, outcomes, and study design (PICOS).	4
**METHODS**			
Protocol and registration	5	Indicate if a review protocol exists, if and where it can be accessed (e.g., Web address), and, if available, provide registration information including registration number.	4
Eligibility criteria	6	Specify study characteristics (e.g., PICOS, length of follow-up) and report characteristics (e.g., years considered, language, publication status) used as criteria for eligibility, giving rationale.	5
Information sources	7	Describe all information sources (e.g., databases with dates of coverage, contact with study authors to identify additional studies) in the search and date last searched.	5
Search	8	Present full electronic search strategy for at least one database, including any limits used, such that it could be repeated.	S2 File
Study selection	9	State the process for selecting studies (i.e., screening, eligibility, included in systematic review, and, if applicable, included in the meta-analysis).	5
Data collection process	10	Describe method of data extraction from reports (e.g., piloted forms, independently, in duplicate) and any processes for obtaining and confirming data from investigators.	5
Data items	11	List and define all variables for which data were sought (e.g., PICOS, funding sources) and any assumptions and simplifications made.	5
Risk of bias in individual studies	12	Describe methods used for assessing risk of bias of individual studies (including specification of whether this was done at the study or outcome level), and how this information is to be used in any data synthesis.	6
Summary measures	13	State the principal summary measures (e.g., risk ratio, difference in means).	6
Synthesis of results	14	Describe the methods of handling data and combining results of studies, if done, including measures of consistency (e.g., I^2) for each meta-analysis.	6

For peer review only - http://bmjopen.bmj.com/site/about/guidelines.xhtml

S1 File: PRISMA Checklist

Section/topic	#	Checklist item	Reported on page #
Risk of bias across studies	15	Specify any assessment of risk of bias that may affect the cumulative evidence (e.g., publication bias, selective reporting within studies).	6
Additional analyses	16	Describe methods of additional analyses (e.g., sensitivity or subgroup analyses, meta-regression), if done, indicating which were pre-specified.	6
**RESULTS**			
Study selection	17	Give numbers of studies screened, assessed for eligibility, and included in the review, with reasons for exclusions at each stage, ideally with a flow diagram.	7
Study characteristics	18	For each study, present characteristics for which data were extracted (e.g., study size, PICOS, follow-up period) and provide the citations.	Table 1
Risk of bias within studies	19	Present data on risk of bias of each study and, if available, any outcome level assessment (see item 12).	Tables 1&2 and S3
Results of individual studies	20	For all outcomes considered (benefits or harms), present, for each study: (a) simple summary data for each intervention group (b) effect estimates and confidence intervals, ideally with a forest plot.	8-10
Synthesis of results	21	Present the main results of the review. If meta-analyses are done, include for each, confidence intervals and measures of consistency.	8-10
Risk of bias across studies	22	Present results of any assessment of risk of bias across studies (see Item 15).	S3 Table & Fig 4
Additional analysis	23	Give results of additional analyses, if done (e.g., sensitivity or subgroup analyses, meta-regression [see Item 16]).	9 (Fig 2-3)
**DISCUSSION**			
Summary of evidence	24	Summarize the main findings including the strength of evidence for each main outcome; consider their relevance to key groups (e.g., healthcare providers, users, and policy makers).	10-11
Limitations	25	Discuss limitations at study and outcome level (e.g., risk of bias), and at review-level (e.g., incomplete retrieval of identified research, reporting bias).	12
Conclusions	26	Provide a general interpretation of the results in the context of other evidence, and implications for future research.	13
**FUNDING**			
Funding	27	Describe sources of funding for the systematic review and other support (e.g., supply of data); role of funders for the systematic review.	14

For peer review only - http://bmjopen.bmj.com/site/about/guidelines.xhtml

From: Moher D, Liberati A, Tetzlaff J, Altman DG, The PRISMA Group (2009). Preferred Reporting Items for Systematic Reviews and Meta-Analyses: The PRISMA Statement. PLoS Med 6(7): e1000097. doi:10.1371/journal.pmed1000097. For more information, visit: **www.prisma-statement.org**.

Database(s): **Ovid MEDLINE(R) Epub Ahead of Print, In-Process & Other Non-Indexed Citations, Ovid MEDLINE(R) Daily and Ovid MEDLINE(R)** 1946 to 25/05/2017
Search Strategy: 24.05.2017

#	Searches	Results
1	exp heart failure/ or (((heart or cardia* or myocardial) adj3 failure*) or (((heart or cardia*) adj3 decompensation*) or ((heart or cardia*) adj3 insufficienc*))).mp.	189098
2	review.ab.	1050955
3	review.pt.	2299071
4	meta-analysis.ab.	82199
5	meta-analysis.pt.	80375
6	or/2-5	2743700
7	letter.pt.	971739
8	comment.pt.	690989
9	editorial.pt.	439919
10	or/7-9	1585046
11	6 not 10	2708683
12	1 and 11	39715
13	(sudden adj3 (cardiac or death*)).ti,ab,kw.	42355
14	12 and 13	1826

For peer review only

Database(s): **Embase** 1974 to 2017 May 23rd
Search Strategy:

#	Searches	Results
1	exp heart failure/ or (((heart or cardia* or myocardial) adj3 failure*) or (((heart or cardia*) adj3 decompensation*) or ((heart or cardia*) adj3 insufficienc*))).mp.	444143
2	meta-analy:.mp.	202659
3	search:.tw.	417814
4	review.pt.	2246810
5	or/2-4	2642821
6	exp Sudden Cardiac Death/ or Sudden Death/ or exp heart arrest/	107134
7	5 and 6	14446
8	1 and 7	9747
9	(sudden adj3 (cardiac or death*)).mp.	76511
10	limit 9 to abstracts	58853
11	8 and 10	3294

For peer review only

S3 Table: AMSTAR scores for included studies

For peer review only

Author (year); country; reference	Q 1	Q 2	Q 3	Q 4	Q 5	Q 6	Q 7	Q 8	Q 9	Q 10	Q 11	AMSTAR score
Al-Gobari M., et al. (2013), France [14]	No	No	Yes	No	No	Yes	Yes	Yes	Yes	Yes	No	6
Chatterjee S., et al. (2013); USA [13]	No	Yes	Yes	Yes	No	Yes	Yes	Yes	Yes	Yes	No	8
Brophy JM. Et al., (2001); Canada [15]	No	No	Yes	No	No	Yes	No	No	Yes	Yes	No	4
Lee S. et al., (2001), USA [16]	No	No	Yes	Yes	No	Yes	No	No	Yes	No	No	4
Bonet S., et al. (2000), USA [17]	No	Yes	Yes	No	No	Yes	No	No	Yes	No	No	4
Heidenreich P.A., et al. (1997); USA [18]	No	Yes	No	Yes	Yes	Yes	No	No	Yes	No	No	5
Le HH. et al., (2016); France [19]	No	Yes	Yes	No	No	Yes	Yes	Yes	Yes	Yes	No	7
Bapoje SR. et al. (2013); USA [52]	No	Yes	Yes	Yes	No	Yes	Yes	Yes	Yes	Yes	No	8
Wei J., et al. (2010); China [20]	No	Yes	Yes	Yes	No	Yes	No	No	Yes	No	No	5
Solomon SD., et al. (2016); USA [21]	No	Yes	Yes	No	No	Yes	Yes	Yes	yes	No	Yes	7
Flather MD et al., (2000); Canada [23]												NA*
Garg R. et al., (1995); Canada [22]	No	Yes	No	No	No	No	No	No	Yes	No	No	2
Rain & Rada, (2015) ; Chile [24*]												NA*
Heran et al., (2012) ; Canada [25]	Yes	Yes	Yes	No	Yes	Yes	Yes	Yes	Yes	Yes	Yes	10
Shibata et al., (2008) ; Canada [26]	No	Yes	No	Yes	No	Yes	No	No	Yes	No	No	4
Lee et al., (2004) ; USA [27]	No	Yes	Yes	Yes	No	Yes	Yes	No	Yes	Yes	No	7
Dimopoulos et al., (2004) ; UK [28]	No	Yes	No	No	No	Yes	No	No	Yes	No	No	3
Jong P., et al. (2002), Canada [29]	Yes	Yes	Yes	No	No	Yes	Yes	Yes	Yes	Yes	No	8
Rain & Rada, (2017); Chile [30*]												NA*
Al-Gobari M., et al. (2017), Switzerland [5]	No	Yes	Yes	No	No	Yes	Yes	Yes	Yes	Yes	No	7
Bonsu KO, et al. (2015), Malaysia [31]	Yes	Yes	Yes	No	No	Yes	Yes	Yes	Yes	Yes	No	8

For peer review only

Wang JQ., et al. (2014); China [32]	No	Yes	Yes	No	No	Yes	Yes	No	No	Yes	No	5
Liu G. et al. (2014); China [33]	No	Yes	Yes	No	No	Yes	Yes	Yes	Yes	Yes	No	7
Rahimi K. et al. (2012); UK [38]	No	Yes	Yes	No	No	Yes	Yes	Yes	Yes	No	No	6
Zhang S. et al. (2011); China [34]	No	Yes	Yes	No	No	Yes	Yes	Yes	Yes	Yes	No	7
Xu M. et al. (2010); China [35]	No	Yes	Yes	Yes	No	Yes	No	No	Yes	Yes	No	6
Lipinski MJ. (2009); USA [36]	No	Yes	Yes	No	No	Yes	Yes	Yes	Yes	Yes	No	7
Levantesi G. et al. (2007); Italy [37]	No	Yes	No	No	No	Yes	No	No	Yes	No	No	3
Claro JC., et al. (2015), Chile [39]	Yes	Yes	Yes	Yes	Yes	Yes	Yes	Yes	Yes	Yes	No	10
Santangeli P. et al. (2012); USA [40]	No	Yes	Yes	Yes	No	Yes	No	No	Yes	No	No	5
Piccini JP., et al. (2009); USA [41]	No	Yes	Yes	No	No	Yes	Yes	Yes	Yes	Yes	No	7
ATMA Investigators (1997) [42]												NA*
Sim I. et al., (1997); USA [43]	No	Yes	Yes	Yes	No	Yes	No	No	Yes	No	No	5
Das MK. et al., (2010): USA [44]*												NA*
Hilleman DE. et al., (2001); USA [45]*												NA*
Rizos EC., et al. (2012); Greece [46]	No	Yes	Yes	No	No	Yes	Yes	Yes	Yes	Yes	Yes	8
Kotwal S. et al. (2012); Australia [47]	No	Yes	Yes	No	No	Yes	Yes	Yes	Yes	Yes	No	7
Kwak SM., et al. (2012); Korea [48]	No	Yes	Yes	No	No	Yes	Yes	Yes	Yes	Yes	Yes	8
Chen Q., et al. (2011); China [49]	No	Yes	Yes	No	No	Yes	Yes	Yes	Yes	Yes	No	7
Marik PE., et al. (2009); USA [50]	No	Yes	Yes	No	No	Yes	No	No	Yes	No	No	4
Wang C., et al. (2006); USA [51]	No	No	Yes	Yes	No	Yes	Yes	Yes	Yes	No	No	6

Q 1: Was an 'a priori' design provided?

Q 2: Was there duplicate study selection and data extraction?

Q 3: Was a comprehensive literature search performed?

Q 4: Was the status of publication (i.e. grey literature) used as an inclusion criterion?

Q 5: Was a list of studies (included and excluded) provided?

Q 6: Were the characteristics of the included studies provided?

Q 7: Was the scientific quality of the included studies assessed and documented?

Q 8: Was the scientific quality of the included studies used appropriately in formulating conclusions?

Q 9: Were the methods used to combine the findings of studies appropriate?

Q 10: Was the likelihood of publication bias assessed?

Q 11: Was the conflict of interest included?

* narrative review, overview or individual patient data meta-analysis (not applicable)

For peer review only

For peer review only

S4 Table: Funding data of included reviews of heart failure patients

Author (year); country; reference	Review type	Intervention/ Comparator	Funding
Al-Gobari M., et al. (2013), France [14]	Systematic review & meta-analysis	Beta-blockers /Placebo; "usual care"	YEMEN LNG company: Private company but non-drug-industry., UMR 5558, BEBL.
Chatterjee S., et al. (2013); USA [13]	Systematic review & meta-analysis	Beta-blockers / Placebo; beta-blocker; "usual care"	None
Brophy JM. Et al., (2001); Canada [15]	Meta-analysis	Beta-blockers / Placebo; "usual care"	Les Fonds de la Recherche en Santé du Québec. Government (non-industry)
Lee S. et al., (2001), USA [16]	systematic review & meta-analysis	Beta-blockers / Placebo	No data reported
Bonet S., et al. (2000), USA [17]	Meta-analysis	Beta-blockers / Placebo ; "usual care"	No data reported
Heidenreich P.A., et al. (1997); USA [18]	Meta-analysis	Beta-blockers / Placebo; "usual care"	No data reported
Le HH. et al., (2016); France [19]	Systematic review & meta-analysis	Anti-aldosterone / Placebo; "usual care"	Non-industry (Academic and Government)
Bapoje SR. et al. (2013); USA [52]	SR and MA	Anti-aldosterone / Placebo; "usual care"	Not reported for included studies. No external fund for the review.
Wei J., et al. (2010); China [20]	Meta-analysis	Anti-aldosterone / Placebo; "usual care"	No data reported
Solomon SD., et al. (2016); USA [21]	Meta-analysis	Sacubitril; valsartan / ACE-i	Data reported for included studies: industry. No funding existed for the review itself.
Flather MD et al., (2000); Canada [23]	Systematic review	ACE-i / Placebo	Industry support: Medical Research Council of Canada and support from Merck Frosst Canada, Merck Sharpe and Dohme, USA, Bristol Myers, Squibb, USA, Zeneca, UK, and Hoechst Marion Roussel, Frankfurt, Germany.
Garg R. et al., (1995); Canada [22]	Systematic review & meta-analysis	ACE-i / Placebo	No data reported
Rain & Rada, (2015) ; Chile [24]	Systematic review	ARB/ ACE-i	No data reported
Heran et al., (2012) ; Canada [25]	Systematic review & meta-analysis (Cochrane)	ARB (or ARB + ACE-i) / Placebo ; ACE-i	Included studies: 23 (96%) industry-sponsored and remaining unknown. Review. External: NIHR, UK Cochrane (Non-industry)
Shibata et al., (2008) ; Canada [26]	Systematic review & meta-analysis	ARB/ Placebo ; ACE-i	No funding exists. Not reported for included studies.
Lee et al., (2004) ; USA [27]	Meta-analysis	ARB/ Placebo ; ACE-i	Industry support: Stock ownership or options (other than mutual funds): D.C. Rhew (Merck, Pfizer); Grants received: Zynx Health Incorporated has received grants from AstraZeneca, Aventis, Bristol-Myers Squibb, Merck, Novartis, and Pfizer.
Dimopoulos et al., (2004) ; UK [28]	Meta-analysis	ARB/ Placebo ; ACE-i	No data reported
Jong P., et al. (2002), Canada [29]	Systematic review & meta-analysis	ARB (or ARB + ACE-i) / Placebo ; ACE-i	No data reported
Rain & Rada, (2017); Chile [30]	systematic review	Statins / Placebo ; "usual care"	No data reported
Al-Gobari M., et al. (2017), Switzerland [5]	Systematic review & meta-analysis	Statins / Placebo ; "usual care"	Yes for the review: non-industry (Governments). Not reported for included studies.

For peer review only

Bonsu KO, et al. (2015), Malaysia [31]	Meta-analysis	Statins / Placebo ; "usual care"	No data reported
Wang JQ., et al. (2014); China [32]	Meta-analysis	Statins / Placebo ; "usual care"	None (reported) but not for included studies.
Liu G. et al. (2014); China [33]	Meta-analysis	Statins / Placebo ; "usual care"	Non-industry-sponsored but no data for included studies.
Rahimi K. et al. (2012); UK [38]	Meta-analysis	Statins / Placebo ; "usual care"	Funding for included studies reported. Some are industry. No funding for the review itself from industry but from Academy for some authors.
Zhang S. et al. (2011); China [34]	Meta-analysis	Statins / Placebo ; "usual care"	Non-industry-sponsored but no data for included studies. Academic, doctoral program…etc
Xu M. et al. (2010); China [35]	Meta-analysis	Statins / Placebo ; "usual care"	No data reported
Lipinski MJ. (2009); USA [36]	Meta-analysis	Statins / Placebo ; "usual care"	No data reported
Levantesi G. et al. (2007); Italy [37]	Meta-analysis	Statins / Placebo ; "usual care"	No data reported
Claro JC., et al. (2015), Chile [39]	Systematic review & meta-analysis (Cochrane)	Amiodarone / Placebo; "usual care"	Funding for the review: Government (non-industry). Not reported for included studies.
Santangeli P. et al. (2012); USA [40]	systematic review	Amiodarone / Placebo	No data reported
Piccini JP., et al. (2009); USA [41]	Meta-analysis	Amiodarone / Placebo ; "usual care"	No external fund for the review. No data reported for included studies.
ATMA Investigators (1997) [42]	Meta-analysis	Amiodarone / Placebo ; "usual care"	No data reported
Sim I. et al., (1997); USA [43]	Meta-analysis	Amiodarone / Placebo ; "usual care"	No data reported
Das MK. et al., (2010): USA [44]	Narrative Review	Anti-arrhythmics / Placebo ; "usual care"	No data reported
Hilleman DE. et al., (2001); USA [45]	Narrative Review	Anti-arrhythmics / Placebo ; "usual care"	No data reported
Rizos EC., et al. (2012); Greece [46]	systematic review & meta-analysis	Omega 3 Fatty acids / Placebo ; "usual care"	Funding of included studies is reported. Conflict of interest too (for one author): industry sponsorship not related to those who manufacture or market Omega-3 suppl.
Kotwal S. et al. (2012); Australia [47]	systematic review & meta-analysis	Omega 3 Fatty acids / Placebo ; "usual care"	Reported but not for included studies. Non-industry.
Kwak SM., et al. (2012); Korea [48]	Meta-analysis	Omega 3 Fatty acids / Placebo ; "usual care"	All included trials sponsored by industry but not reported for the review itself.
Chen Q., et al. (2011); China [49]	Meta-analysis	Omega 3 Fatty acids / Placebo ; "usual care"	No data reported
Marik PE., et al. (2009); USA [50]	systematic review	Omega 3 dietary supplements / Placebo; olive oil ; corn oil, sunflower oil ; "usual care"	No data reported
Wang C., et al. (2006); USA [51]	systematic review	n-3 Fatty acids / Placebo/olive oil ; corn oil, sunflower oil ; "usual care"	Internal by saying who obtained funds but no specific source. No data for included studies.

For peer review only

BMJ Open

Effectiveness of drug interventions to prevent sudden cardiac death in patients with heart failure and reduced ejection fraction: an overview of systematic reviews

Journal:	*BMJ Open*
Manuscript ID	bmjopen-2017-021108.R2
Article Type:	Research
Date Submitted by the Author:	12-Jun-2018
Complete List of Authors:	Al-Gobari, Muaamar; Lausanne university Hospital (CHUV), Institute of social & preventive medicine (IUMSP), Cochrane Switzerland<br>Al-Aqeel, Sinaa; King Saud University , Clinical Pharmacy<br>Gueyffier, François; Université Claude Bernard Lyon 1, UMR5558; Hospices Civils de Lyon, Service de Pharmacologie clinique<br>Burnand, Bernard; Lausanne university Hospital (CHUV), Institute of social & preventive medicine (IUMSP), Cochrane Switzerland
**Primary Subject Heading**:	Cardiovascular medicine
Secondary Subject Heading:	Evidence based practice
Keywords:	cardiac failure, sudden death, umbrella review, meta-analysis, guidelines, treatment

SCHOLARONE™ Manuscripts

only

Effectiveness of drug interventions to prevent sudden cardiac death in patients with heart failure and reduced ejection fraction: an overview of systematic reviews

Muaamar Al-Gobari[1*], Sinaa Al-Aqeel[2], François Gueyffier[3], Bernard Burnand[1]

[1] Institute of social & preventive medicine (IUMSP) and Cochrane Switzerland, Lausanne University Hospital (CHUV), Lausanne, Switzerland

[2] Clinical Pharmacy Department, College of Pharmacy, King Saud University, Riyadh, Kingdom of Saudi Arabia.

[3] Laboratoire de Biologie et Biométrie Evolutive-Equipe Modélisation des Effets Thérapeutiques, UMR 5558 Université Claude Bernard Lyon1, Lyon, France

*** Corresponding Author Information:**

Institute of social & preventive medicine (IUMSP)

Biopôle 2, SV-A

Route de la Corniche 10

CH-1010 Lausanne, Switzerland

Tel. +41 21 314 51 45

E-mail: muaamar.algobari@gmail.com

For peer review only - http://bmjopen.bmj.com/site/about/guidelines.xhtml

Abstract

Objectives

To summarize and synthesize the current evidence regarding the effectiveness of drug interventions to prevent sudden cardiac death (SCD) and all-cause mortality in heart failure with reduced ejection fraction (HFrEF) patients.

Design

Overview of systematic reviews.

Data sources

Medline, Embase, ISI Web of Science and Cochrane Library from inception to May 2017; manual search of references of included studies for potentially relevant reviews.

Eligibility criteria for study selection

We reviewed the effectiveness of drug interventions for SCD and all-cause mortality prevention in HFrEF patients. We included overviews, systematic reviews and meta-analyses of randomized controlled trials of beta-blockers, angiotensin-converting enzyme inhibitors (ACE-i), angiotensin receptor blockers (ARBs), anti-aldosterones or mineralocorticoid-receptor antagonists, amiodarone, other antiarrhythmic drugs, combined angiotensin receptor blocker (ARB)/neprilysin inhibitors, statins and fish oil supplementation.

Review methods

Two independent reviewers extracted data and assessed the methodological quality of the reviews and the quality of evidence for the primary studies for each drug intervention, using AMSTAR and GRADE respectively.

Results

We identified 41 reviews. Beta-blockers, anti-aldosterones, and combined ARB/neprilysin inhibitors appeared effective to prevent SCD and all-cause mortality. ACE-i significantly reduced all-cause mortality but not SCD events. ARBs and statins were ineffective where antiarrhythmic drugs and omega-3 fatty acids had unclear evidence of effectiveness for prevention of SCD and all-cause mortality.

For peer review only - http://bmjopen.bmj.com/site/about/guidelines.xhtml

Conclusions

This comprehensive overview of systematic reviews confirms that beta-blockers, anti-aldosterone agents and combined ARB/neprilysin inhibitors are effective on SCD prevention but not ACE-i or ARBs. In patients with high risk of SCD, an alternative therapeutic strategy should be explored in future research.

Systematic Review Registration: PROSPERO 2017: CRD42017067442

Key Words

cardiac failure, sudden death, umbrella review, treatment, pharmacotherapy, meta-analysis, guidelines, evidence-based medicine

Strengths and limitations of this study

- A major strength of our study is that it summarizes and synthesizes the effectiveness of most evidence-based drug interventions in heart failure patients with reduced ejection fraction for sudden cardiac death (SCD) prevention and classified drug interventions according to the current evidence of their effectiveness.
- Our study used data from published studies and no data from unpublished studies.
- Our study reviews most heart failure drugs on the prevention of SCD and all-cause mortality but limited in scope for not including some drugs such as digoxin, ivabradine, and non-drug interventions/devices such as implantable cardioverter defibrillators.

For peer review only

For peer review only - http://bmjopen.bmj.com/site/about/guidelines.xhtml

Introduction

Heart failure (HF) morbidity and mortality constitute an important burden for patients and for the healthcare systems in both developed and developing countries [1]. HF patients are frequently hospitalized and not only have a high mortality risk from a bad prognosis, but also unexpectedly, termed sudden cardiac death (SCD). In people diagnosed with HF, SCD occurs at 6–9 times the rate of the general population. Almost 20% and 80% of patients die within one year and eight years of initial diagnosis, respectively [1 2]. Risk factors of SCD were reported to be similar to cardiovascular diseases. However, the most studied and proven predictor of SCD in HF patients is left ventricular ejection fraction (LVEF)[3]. Potential drug interventions in heart failure with reduced ejection fraction (HFrEF) patients include beta-blockers (BBs), angiotensin-converting enzyme inhibitors (ACE-i), angiotensin receptor blockers (ARBs), anti-aldosterones or mineralocorticoid-receptor antagonists, amiodarone, other antiarrhythmic agents, combined angiotensin receptor blocker/neprilysin inhibitors, statins and fish oil supplementation [4]. Some of these interventions aimed at improving survival and reducing total mortality and SCD in HF. For instance, a newly licensed drug (Sacubitril/valsartan) in Paradigm-HF trial showed around 20% SCD reduction compared to enalapril [5].

Nevertheless, optimal strategies for SCD prevention in HF are warranted if we take into account the high portion of mortality that still occurs in this population. Had a practitioner identified a patient with high risk of SCD, it would be important to know which drug is effective or not in SCD prevention other than non-drug interventions such as implantable cardioverter defibrillators (ICDs). However, the large amount of information and the multiple and sometimes discordant systematic reviews on drug interventions could be misleading [6].

Therefore, it is vital to identify the pharmacological agents that confer the greatest benefit in SCD risk reduction particularly in high-risk patients and if any optimization of therapeutic strategies to those patients is possible accordingly. Thus, we decided to conduct an overview of systematic reviews to summarize and synthesize the available evidence about the effectiveness of drug interventions in the prevention of SCD in HFrEF, and categorized the evidence into effective, ineffective and unclear evidence of effectiveness.

For peer review only - http://bmjopen.bmj.com/site/about/guidelines.xhtml

Methods

We developed an a priori protocol for this review according to the Preferred Reporting Items for Systematic Reviews and Meta-Analyses (PRISMA) statement (**S1 File**) and registered it in the PROSPERO International prospective register of systematic reviews (CRD42017067442).

Data sources and search strategy

Using the Ovid online interface, we searched Medline (up to May 24, 2017), Embase (up to May 23, 2017), ISI web of science and the Cochrane library (up to May 24, 2017). We identified overviews, systematic reviews and meta-analyses of randomized clinical trials by means of a search strategy (available on **S2 File**). The search strategy was composed of a filter [7 8], a mixture of medical subject heading terms [Mesh and EMTREE in Medline and Embase respectively], text words as well as a truncation when possible without any language or publication date restriction. We did not search conference proceedings nor the grey literature. Reference lists of the included reviews were manually checked for any additional eligible studies. We contacted corresponding reviews' and primary studies' authors to seek for relevant unreported data. If judged necessary, we intended to update the included reviews by searching primary studies published after the systematic review publication date. Apart from authors' expertise in the field, we decided to update if the most up-to-date review of a drug intervention was published more than 5 years ago and/or new clinical trials are not integrated into the evidence.

Selection criteria and data abstraction

Studies were eligible if they were overviews, systematic reviews and meta-analyses of randomized clinical trials that evaluated the effectiveness of drug interventions in HFrEF patients. Reviews were included if they examined the effectiveness of the following drugs: BBs, ACE-i, ARBs, anti-aldosterones or mineralocorticoid-receptor antagonists, amiodarone, antiarrythmics, combined ARB/neprilysin inhibitors, statins and fish oil supplementation. The selected reviews should have contained at least one of the aforementioned HF therapy and had evaluated SCD and/or all-cause mortality prevention as outcomes. We used Endnote and Rayyan[9] to remove duplicates during the selection based on titles and abstracts, and full-text screening.

The abstracted data included eligibility criteria, population type, ejection fraction, study design (including intervention and comparator arms), follow-up duration, and authors' evaluation of outcomes. Two reviewers (MA&SA) independently abstracted data. We resolved discrepancies by consensus or by adding a third reviewer's judgment, when necessary.

For peer review only - http://bmjopen.bmj.com/site/about/guidelines.xhtml

Quality assessment of the included reviews

Methodological quality of the included reviews

Two authors (MA&SA) independently used the AMSTAR measurement tool to assess systematic reviews included in our overview. The AMSTAR checklist comprises eleven questions (**S3 table**) and each question accounted for one score point [10]. The answer of "yes" gave a score of 1 and zero otherwise. This increasingly adopted tool was used at the data collection step as stipulated in the overview protocol[11]. If the authors of included reviews failed to publish their protocol, we deducted a score of one. In addition, we scored "yes" if the authors mentioned that two reviewers were involved in the study screening, selection or data extraction.

Quality of evidence in the included reviews

Two authors (MA&SA) independently used the GRADE (Grading of Recommendations, Assessment, Development and Evaluation) approach [12] to assess the quality of evidence of each intervention. GRADE is a widely accepted tool that allows the assessment of five key elements: risk of bias, inconsistency, indirectness, imprecision, and publication bias. GRADE categorizes the quality of evidence into four levels: high, moderate, low and very low. In the presence of a high risk of bias, the quality of the evidence is downgraded from high to moderate and so on. We also reported the GRADE assessments reported by the authors of the included reviews, or assessed them otherwise. Moreover, we did not reassess the risk of bias at primary study level if authors of included reviews had sufficiently assessed their quality. In the case of the updated review of angiotensin receptor blockers, however, we assessed the quality of newly added randomized clinical trials and integrated it into the evidence synthesis.

Statistical analysis and data synthesis

We provided a narrative synthesis of the findings of the included reviews and if multiple reviews existed for the same intervention. However, in the case of ARBs, we updated the evidence and meta-analyzed the data using random-effects and fixed-effects model with Mantel-Haenszel methods [13] and reported random-effects model to account for heterogeneity. Meanwhile, we evaluated each intervention against our outcomes of interest and synthesized the evidence taking into account heterogeneity and inconsistencies between reviews. As a rule of thumb, I^2 (I-square) values of 25%, 50% and 75% correspond to low, moderate and high levels of heterogeneity, respectively [14].

For peer review only - http://bmjopen.bmj.com/site/about/guidelines.xhtml

For the purpose of our overview, we categorized the evidence of the included interventions into three categories: 1) effective interventions; 2) ineffective interventions; 3) uncertain evidence (conflicting or inconclusive evidence). We used relative effect [odds ratio (OR) or risk ratio (RR)] as a summary statistic from the most recent or largest published systematic reviews, and confidence intervals (CIs) of 95% with a significance level determined at two-sided alpha less than 5%.

Patient and Public Involvement

Our study did not involve direct contact with patients or the public

For peer review

Results

Search result

According to our pre-defined eligibility criteria, our search strategy in electronic databases and manual searches resulted in 41 studies [6 15-54]. Fig 1 shows the search strategy results. At full-text level, we excluded studies that did not assess our outcome of interest (n=129), were narrative reviews (n=4), did not include HF patients (n=8), included preserved heart failure patients (n= 3), were duplicate or had no full text (n=2).

Characteristics of the included reviews

As shown in **Table 1**, the population of the included reviews consisted of HF patients with an ejection fraction ≤ 45% in most studies and a corresponding New York Heart Association (NYHA) classification ranging from I-IV. The effectiveness of each drug intervention has been assessed in at least one review. All reviews were systematic, except two reviews for anti-arrhythmic drugs. At the time of their publication, 15 out of 41 reviews (37%) had corresponding authors based in the USA, 7 (17%) in Canada, 6 (15%) in China, 3 in Chile, 2 in France, 2 in the UK, and the 6 remaining in other countries.

The disclosure and reporting of financial resources or funding varied from one study to another. Twenty-one reviews (51%) did not report the source of funding. Ten reviews (24 %) reported financial supports that included governments, academic institutions, and device industry. Six reviews declared financial resources as none or no external funds. Three reviews reported industry sponsorship for at

For peer review only - http://bmjopen.bmj.com/site/about/guidelines.xhtml

least one author. One review [53] stated that one author obtained funds for the review without clarifying the source (**S4 table**). We also reported findings summary of each review as stated by their respective authors (**Table 1**).

For peer review only

For peer review only - http://bmjopen.bmj.com/site/about/guidelines.xhtml

Table 1: Summary of characteristics of included studies of heart failure (ordered by intervention)

Author (year); country; reference	Review type	Intervention/ Comparator	Population type; Ejection fraction (%); NYHA	Study design n; participants n	Mean follow-up/Range (months)	Authors' Findings summary	AMSTAR score
Al-Gobari M., et al. (2013), France [16≠]	Systematic review & meta-analysis	Beta-blockers /Placebo; "usual care"	HF; <45% except one study < 62%; I-IV	RCTs n= 30; n = 24,779	Mean: 11.51	Beta-blockers significantly reduced SCD, cardiovascular death and all-cause mortality	6
Chatterjee S., et al. (2013); USA [15]	Systematic review & meta-analysis	Beta-blockers / Placebo; beta-blocker; "usual care"	HF; <45%; II-IV	RCTs n=21 ; n= 23,122	median: 12	The study confirmed mortality benefits of BBs compared to placebo or usual care in heart failure with reduced ejection fraction.	8
Brophy JM. Et al., (2001); Canada [17]	Meta-analysis	Beta-blockers / Placebo; "usual care"	CHF; <45%; I-IV	RCTs n= 22 ; n= 10,135	Range: 3 to 23	This study reported a reduction in mortality and morbidity in CHF.	4
Lee S. et al., (2001), USA [18]	systematic review & meta-analysis	Beta-blockers / Placebo	HF; <30%; II-III	RCTs n=6 ; n= 9335	Range : 12 to 23	The authors recommended use of beta-blockers in HF with reduced ejection fraction and NYHA II-III.	4
Bonet S., et al. (2000), USA [19]	Meta-analysis	Beta-blockers / Placebo ; "usual care"	HF; <45%; NA	RCTs n= 21 ; n= 5,849	Median: 6	Beta-blockers reduce total mortality by reducing pump failure and sudden cardiac death events. Vasodilating beta-blockers have perhaps greater effects on overall mortality than nonvasodilating agents.	4
Heidenreich P.A., et al. (1997); USA [20]	Meta-analysis	Beta-blockers / Placebo; "usual care"	HF; <30%; I-IV	RCTs n= 17 ; n= 3,039	Range: 3 to 24	Beta-blockers significantly reduced all-cause mortality but showed a trend toward better reduction in nonsudden cardiac death compared to sudden cardiac death.	5
Le HH. et al., (2016); France [21≠]	Systematic review & meta-analysis	Anti-aldosterone / Placebo; "usual care"	HF, post-MI; <40%- >50%; I-IV	RCTs n=25; n = 19,333	Range: 3 to 39.6	In HF, anti-aldosterones or mineralocorticoid receptor blockers reduced SCD (subgroup analysis: 5 RCTs), all-cause mortality (subgroup analysis: 10 RCTs) and cardiovascular, all-cause and cardiovascular hospitalization. Adverse effects (hyperkalemia, degradation of renal function, gynecomastia) were, however, significantly higher in the treated group compared to placebo.	7
Bapoje SR. et al. (2013); USA [54]	Systematic review & meta-analysis	Anti-aldosterone / Placebo; "usual care"	HF; <45%; I-IV	RCTs n= 8; n= 11,875	Range: 3 to 24	Mineralocorticoid receptor antagonists (or aldosterone antagonists) reduced the risk of SCD in patients with left ventricular dysfunction.	8
Wei J., et al. (2010); China [22]	Meta-analysis	Anti-aldosterone / Placebo; "usual care"	HF; <45%; NA1	RCTs n= 6 (two are not double blind), n= 00000	Range: 2 to 24	Two [Pitt et al, 1995 and Pitt et al 2003] of the six-included studies showed a significant reduction of SCD in the group of Spironolactone vs placebo and the group of Eplerenone vs. Placebo, cited respectively.	5
Solomon SD., et al. (2016); USA [23≠]	Meta-analysis	Sacubitril; valsartan / ACE-i	HF; <30%; II-IV	RCTs n= 3; n= 14742	Range 6 to 27	The authors concluded that combined neprilysin/RAS inhibition reduced all-cause mortality in HFrEF.	7
Flather MD et al., (2000); Canada [25]	Systematic review	ACE-i / Placebo	CHF; post-MI <45%; NA	RCTs n= 5, n= 12,763	Range: 15 to 42	This meta-analysis showed a lower risk of death in ACE-i treated group compared to placebo.	NA
Garg R. et al., (1995); Canada [24≠]	Systematic review & meta-analysis	ACE-i / Placebo	CHF; <45% ; I-IV	RCTs n= 32, n= 7,105	Range: 3 to 42	Overall, this study reported a significant reduction of total mortality (attributed mainly to less progressive HF deaths) and hospitalization for worsening HF.	2

For peer review only - http://bmjopen.bmj.com/site/about/guidelines.xhtml

Rain & Rada, (2015) ; Chile [26]	Systematic review	ARB/ ACE-i	HF ; <45% - < 35% ; II-IV	RCTS = 8 ; n= 5201	NA	The authors concluded that ARBs are probably as effective in mortality reduction as ACE-i with probably less withdrawal rate due to adverse effects.	NA
Heran et al., (2012) ; Canada [27*]	Systematic review & meta-analysis (Cochrane)	ARB (or ARB + ACE-i) / Placebo ; ACE-i	HF ; ≤40% ; II-IV	RCTS n= 24 ; n= 25,051	Range: 1 to 49.5	Compared to placebo or in addition to ACE-i, ARBs did not reduce all-cause mortality.	10
Shibata et al., (2008) ; Canada [28]	Systematic review & meta-analysis	ARB/ Placebo ; ACE-i	HF ; <40% ; I-IV	RCTs n= 7 ; n= 27,495	Range: 11 to 41	Compared to ACE-i or used in combination, ARBs provided no beneficial effects on mortality. A 17% reduction in hospitalizations was observed.	4
Lee et al., (2004) ; USA [29]	Meta-analysis	ARB/ Placebo ; ACE-i	CHF , AMI ; ≤45% ; II–IV	RCTs n= 24 ; n= 38,080	Range: 1 to 41	Compared to ACE-i, ARBs do not differ in efficacy for reducing all-cause mortality in CHF and AMI patients.	7
Dimopoulos et al., (2004) ; UK [30]	Meta-analysis	ARB/ Placebo ; ACE-i	CHF; <40% ; II–IV	RCTs n= 4; n = 7666	Mean: 31	ARBs can be used to prevent events in ACE-i-treated heart failure patients who are not suitable for beta-blockers.	3
Jong P., et al. (2002), Canada [31]	Systematic review & meta-analysis	ARB (or ARB + ACE-i) / Placebo ; ACE-i	HF; ≤35% - ≤45%; II–IV	RCTs n= 17; n = 12,469	Range: 1 to 23	The authors could not conclude any superiority of ARBs versus controls, stating this might be due to the use of ACE-i as a comparator or background treatment in the majority of included trials.	8
Rain & Rada, (2017); Chile [32]	systematic review	Statins / Placebo ; "usual care"	HF; <45%; I-IV	RCTs n= 25; n = NR	NA	The authors summarized that statins do not decrease mortality in chronic heart failure and might lead to a small reduction in hospital admissions for HF.	NA
Al-Gobari M., et al. (2017), Switzerland [6≠]	Systematic review & meta-analysis	Statins / Placebo ; "usual care"	HF, ischaemic/nonischaemic; NA; I-IV NA	RCTs n= 24; n = 11,463	Range: 1 to 46.8	Statins do not significantly reduced SCD and all-cause mortality. They may or may not reduce hospitalizations due to worsening heart failure (HWHF)	7
Bonsu KO, et al. (2015), Malaysia [33]	Meta-analysis	Statins / Placebo ; "usual care"	HF; <45%; I-IV	RCTs n= 13; n = 10,966	Range: 3 to 46.8	Lipophilic statins showed significant decrease in all-cause mortality, cardiovascular mortality and hospitalization for worsening heart failure.	8
Wang JQ., et al. (2014); China [34]	Meta-analysis	Statins / Placebo ; "usual care"	HF; NA; NA	RCTs n= 6 (9: observational studies), n= 10,016	Range: 12 to 46.8	The authors concluded that statins reduce sudden cardiac death and all-cause mortality in HF.	5
Liu G. et al. (2014); China [35]	Meta-analysis	Statins / Placebo ; "usual care"	HF; <45%; I-IV	RCTs n=13 ; n = 1,532	Range: 3 to 35.5	The authors reported significant decrease in all-cause mortality but recommended cautious interpretation and further research.	7
Rahimi K. et al. (2012); UK [40]	Meta-analysis	Statins / Placebo ; "usual care"	HF, MI, Primary prevention, diabetes, ACS, CHD; NA; NA	RCTs n= 37 n= 155,020		Statins have a modest effect on SCD but no substantial protective effect on ventricular arrhythmic events.	6
Zhang S. et al. (2011); China [36]	Meta-analysis	Statins / Placebo ; "usual care"	HF; <45%; I-IV	RCTs n=13 ; n = 10,447	Range: 2 to 46.8	This meta-analysis concluded of no difference between treatment groups but benefits may occur in some specific populations and with a specific statin.	7
Xu M. et al. (2010); China [37]	Meta-analysis	Statins / Placebo ; "usual care"	HF; <45%; I-IV	RCTs n= 7, n= 540	Range: 3 to 31	The authors suggested that atorvastatin treatment is effective and reduce all-cause mortality and hospitalization for worsening HF.	6
Lipinski MJ. (2009); USA [38]	Meta-analysis	Statins / Placebo ; "usual care"	HF; <45%; I-IV	RCTS n= 10; n= 10,192	Range 3 to 47	The authors stated that statins are safe and improve LVEF and decrease hospitalization for worsening HF.	7
Levantesi G. et al. (2007); Italy [39]	Meta-analysis	Statins / Placebo ; "usual care"	Secondary prevention; NA; NA	RCTs n= 10 n= 22,275	Range: 6 to 73.2	Statins were associated with a significant risk reduction for SCD (in secondary prevention settings).	3
Claro JC., et al. (2015), Chile [41≠]	Systematic review & meta-	Amiodarone / Placebo; "usual care"	Sub-analysis : heart failure ; NA; NA	RCTs n= 11, n= 5,006	NA	In HF sub-population, amiodarone showed a statistically significant reduction for SCD but not for all-cause mortality. Authors judged the quality of the evidence for the whole	10

For peer review only

For peer review only - http://bmjopen.bmj.com/site/about/guidelines.xhtml

	analysis (Cochrane)					population (primary prevention) as low to moderate and for secondary prevention population as very low.	
Santangeli P. et al. (2012); USA [42]	systematic review	Amiodarone / Placebo	cardiovascular disease; NA; NA	NA	NA	Amiodarone has less favorable net clinical benefits for prophylaxis of SCD because of adverse effects.	5
Piccini JP., et al. (2009); USA [43]	Meta-analysis	Amiodarone / Placebo ; "usual care"	HF, AMI; <45%; II-IV	RCTs n= 15, n= 8,522	Range: 2 to 12	In HF sub-population, amiodarone showed a statistically significant reduction for SCD but not all-cause mortality.	7
ATMA Investigators (1997) [44]	Meta-analysis	Amiodarone / Placebo ; "usual care"	Post-MI and CHF; 31%; NA	NA	Range: 4.8 to 25.8	Amiodarone reduced arrhythmic/sudden death in high-risk patients with recent MI or CHF. All-cause mortality decreased by 13 %.	NA
Sim I. et al., (1997); USA [45]	Meta-analysis	Amiodarone / Placebo ; "usual care"	Subgroup : heart failure; <45%; NA	RCTs n=5, n= 4,125	Range: 6 to 45.6	Amiodarone reduced all-cause mortality in high SCD risk groups.	5
Das MK. et al., (2010): USA [46]	Narrative Review	Anti-arrhythmics / Placebo ; "usual care"	Subgroup : heart failure; NA; NA	NA	NA	Class I Anti-arrhythmic drugs (AADs) increased all-cause mortality and SCD in Post-MI patients. Amiodarone (class III AADs) decreased or have neutral effect on SCD. Caution is warranted to outweigh risks of proarrhythmia and other adverse effects.	NA
Hilleman DE. et al., (2001); USA [47]	Narrative Review	Anti-arrhythmics / Placebo ; "usual care"	HF; <45%; NA	RCTs n= 6 n= 10440	Range: 6.5 to 45	Beta-blockers (Bisoprolol, Carvedilol, Metoprolol) reduced total mortality and SCD in HF. Class I antiarryhthmics increased mortality and SCD in a post hoc analysis of SPAF-I study. Amiodarone had mixed results and dofetilide did not reduce mortality or SCD.	NA
Rizos EC., et al. (2012); Greece [48]	systematic review & meta-analysis	Omega 3 Fatty acids / Placebo ; "usual care"	cardiovascular diseases; NA; NA	RCTs n= 20; n= 68,680	NA	Omega-3 PUFAs supplementation were not associated with a lower risk of all-cause mortality or sudden cardiac death	8
Kotwal S. et al. (2012); Australia [49]	systematic review & meta-analysis	Omega 3 Fatty acids / Placebo ; "usual care"	cardiovascular diseases, heart failure admissions; NA; NA	RCTs n= 20; n= 62,851	Range: 6 to 72	The authors concluded that there is no clear effect on total mortality and sudden death outcomes.	7
Kwak SM., et al. (2012); Korea [50]	Meta-analysis	Omega 3 Fatty acids / Placebo ; "usual care"	secondary prevention of cardiovascular disease; NA; NA	RCTs n=14; n= 20,485	NA	This meta-analysis concluded of insufficient evidence.	8
Chen Q., et al. (2011); China [51]	Meta-analysis	Omega 3 Fatty acids / Placebo ; "usual care"	cardiovascular disease; NA; NA	RCTs n= 10; n= 33,429	NA	Omega-3 fatty acids did not appear to reduce SCD under guideline-adjusted treatment for CVD secondary prevention.	7
Marik PE., et al. (2009); USA [52]	systematic review	Omega 3 dietary supplements / Placebo; olive oil ; corn oil, sunflower oil ; "usual care"	cardiovascular disease; NA; NA	RCTs n= 11; n= 39,044	Range: 12 to 55.2	Dietary supplementation with omega-3 fatty acids reduced SCD and all-cause mortality	4
Wang C., et al. (2006); USA [53]	systematic review	n-3 Fatty acids / Placebo/olive oil ; corn oil, sunflower oil ; "usual care"	Primary and secondary prevention; NA; NA	RCTs n= 12; n= 32,981	Range: 12 to 48	The authors concluded of a significant reduction in all-cause mortality and SCD with n-3 Fatty acids from fish or fish-oil supplements but not α-linolenic acid.	6

≠: studies considered in Table 2 as "reference" studies. *: updated in this study. NA: not applicable

For peer review only

Risk of bias and quality of reviews

As shown in **Table 1**, the AMSTAR scores for quality assessment of the included reviews widely ranged from 2 to 10 (out of 11). All reviews had one score less because of non-listing of excluded primary studies except Cochrane reviews [27 41] which scored 10 because of non-inclusion of grey literature in the search strategy in one review and missing information for funding resources of included primary studies in another, cited respectively (**S3 Table**). We did not assess the AMSTAR score for six studies, of which two [46 47] were narrative reviews, two[25 44] were individual participant or patient data (IPD) meta-analyses, and the other two [26 32] were overviews of reviews.

The risk of bias of the included primary studies within reviews remained as judged by the original reviews' authors with the exception of the newly added randomized trials in the update of the angiotensin receptor blockers review that we assessed (GRADE) (**Table 2**). The quality of evidence for beta-blockers and anti-aldosterone agents obtained a high quality on the GRADE scale, while angiotensin-converting enzyme inhibitors (ACE-i), amiodarone and statins obtained a moderate quality. However, combined ARB/neprilysin inhibitors had a moderate and high quality for SCD and all-cause mortality outcomes respectively, whereas angiotensin receptor blockers had a low quality of evidence (**Table 2**).

Table 2: Summary of findings and Grade evaluation for sudden cardiac death (SCD) and all-cause mortality prevention

Drug interventions for SCD and all-cause mortality prevention in heart failure patients							
Outcome	Intervention / comparison	Assumed risk with comparator	Corresponding risk with intervention	Relative effect (95% CI)	Number of participants (No of studies)	Quality of the evidence (GRADE)	Comments
**Sudden cardiac death**							
	Beta-blockers / placebo	77 per 1000	54 per 1000 (49 to 60)	OR 0.69 (0.62 to 0.77)	24,779 (26 RCTs)	⊕⊕⊕⊕ high*	I^2=0% (P=0.57)
	Anti-aldosterone inhibitor / Placebo ;	61 per 1000	49 per 1000 (41 to 60)	RR 0.81 (0.67 to 0.98)	8,301 (5 RCTs)	⊕⊕⊕⊕ high*	I^2=8% (P=0.36)

For peer review only - http://bmjopen.bmj.com/site/about/guidelines.xhtml

	"usual care"						
	ARB;Neprilysin inhibitor/ ACE-i	74 per 1000	60 per 1000 (51 to 70)	RR 0.81 (0.69 to 0.95)	8,399 (1 RCsT)	⊕⊕⊕⊖ moderate[e]	
	ACE-i / Placebo	59 per 1000	54 per 1000 (43 to 65)	OR 0.91 (0.73 to 1.11)	6,988 (30 RCTs)	⊕⊕⊕⊖ moderate[b]	I^2=0% (P=0.94)
	ARB (or ARB + ACE-i) / Placebo ; ACE-i	See comment	See comment	Not estimable	13,884 (5 RCTs)	⊕⊕⊖⊖ Low[b,c]	I^2=78% (P=0.010). Overall, we did not pool the studies because of heterogeneity
	Statins / placebo ; "usual care"	108 per 1000	100 per 1000 (76 to 131) [99 per 1000 (72 to 131)]	RR 0.92 (0.7 to 1.21) [OR 0.90 (0.64 to 1.24)]	10,077 (8 RCTs)	⊕⊕⊕⊖ moderate[a]	I^2=42.6% (P=0.094
	Amiodarone / Placebo; "usual care"	118 per 1000	93 per 1000 (79 to 110)	RR 0.79 (0.67 to 0.93)	5,006 (11 RCTs)	⊕⊕⊖⊖ Low[a,b]	
	Omega 3 Fatty acids / Placebo ; "usual care"	93 per 1000	88 per 1000 (77 to 102)	RR 0.94 (0.82 to 1.09)	6,975 (1 RCT)	⊕⊕⊕⊖ moderate[e]	
**All-cause mortality**							
	Beta-blockers / placebo	178 per 1000	127 per 1000 (113 to 141)	OR 0.67 (0.59 to 0.76)	24,779 (26 RCTs)	⊕⊕⊕⊕ high*	I^2= 40% (P=0.02)
	Anti-aldosterone inhibitor / Placebo ; "usual care"	200 per 1000	162 per 1000 (148 to 176)	RR 0.81 (0.74 to 0.88)	9,019 (10 RCTs)	⊕⊕⊕⊕ high*	I^2=0% (P=0.56)
	ARB;Neprilysin inhibitor/ ACE-i	183 per 1000	158 per 1000 (145 to 172)	RR 0.86 (0.79 to 0.94)	14,742 (3 RCTs)	⊕⊕⊕⊕ high	I^2=0% (P=0.42)

For peer review only - http://bmjopen.bmj.com/site/about/guidelines.xhtml

For peer review only

	ACE-i / Placebo	219 per 1000	178 per 1000 (158 to 198)	OR 0.77 (0.67 to 0.88)	7,105 (32 RCTs)	⊕⊕⊕⊖ moderate[b]	I^2=0% (P=0.95)
	ARB (or ARB + ACE-i) / Placebo ; ACE-i	183 per 1000	177 per 1000 (161 to 197)	RR 0.97 (0.88 to 1.08)	19,510 (27 RCTs)	⊕⊕⊖⊖ Low[b,d]	I^2=24% (P=0.14)
	Statins / placebo ; "usual care"	273 per 1000	240 per 1000 (205 to 278) [233 per 1000 (199 to 273)]	RR 0.88 (0.75 to 1.02) OR 0.81 (0.66 to 1)	11,024 (13 RCTs)	⊕⊕⊕⊖ moderate[a]	I^2=37.7% (P=0.083)
	Amiodarone / Placebo ; "usual care"	264 per 1000	237 per 1000 (211 to 266)	RR 0.90 (0.80 to 1.01)	5,006 (11 RCTs)	⊕⊕⊖⊖ Low[a,b]	
	Omega 3 Fatty acids / Placebo ; "usual care"	291 per 1000	274 per 1000 (253 to 294)	RR 0.94 (0.87 to 1.01)	6,975 (1 RCT)	⊕⊕⊕⊖ moderate[e]	

[a] Likelihood of Publication bias presence with an asymmetric funnel plot.

[b]The studies reported to generally have a moderate to high risk of bias due to allocation concealment and blinding reporting.

[c] Inconsistent results ranged from no effect to insignificant increase of events (I^2 = 71%)

[d] Most studies have small sample and wide confidence intervals including no effect with appreciable harm or benefit.

[e] Estimation comes from one single clinical trial. No data obtained from other relevant studies for this outcome

* Although graded high, this might be downgraded into moderate if we strictly consider the risk of bias of primary studies other than an overall estimation.

RR: risk ratio; OR: odds ratio; CI: confidence interval; GRADE: Grading of Recommendations, Assessment, Development and Evaluation; I^2: between-study variance due to heterogeneity.

For peer review only - http://bmjopen.bmj.com/site/about/guidelines.xhtml

For peer review only

For peer review only

For peer review only - http://bmjopen.bmj.com/site/about/guidelines.xhtml

Up-to-dateness of included reviews

Most retrieved evidence was published within the last 10 years (2008 and on) and seven (out of nine) drug interventions with updated systematic reviews were within the last five years (2012 and on). Moreover, we updated the pooled results for ARBs, which resulted in slightly different results compared to the original Cochrane review [27].

Effectiveness of interventions

We report below the summaries of our evaluation on the effectiveness of the drug interventions considered that we have categorized into effective, ineffective, and uncertain effectiveness (inconclusive or conflicting evidence).

Effective interventions

Beta-blockers (BBs)

Meta-analyses and systematic reviews of randomized controlled trials [15-20] provided overwhelming evidence that beta-blockers (BBs) decrease the risk of SCD and all-cause mortality in HFrEF patients. The quality of the evidence was rated high with a relative effect of 0.69 for SCD [OR, 95% CI (0.62 to 0.77)], and of 0.67 for all-cause mortality [OR, 95% CI (0.59 to 0.76)] (**Table 2**).

Anti-aldosterone agents

Published studies about mineralocorticoid-receptor antagonists (antimineralocorticoids) or (so-called) anti-aldosterones appeared effective in SCD and all-cause mortality prevention [21 22 54]. However, in a recent systematic review [21], adverse effects (hyperkalemia, degradation of renal function, gynecomastia) were significantly higher in the anti-aldosterone-treated group compared to placebo. The quality of the evidence was rated high with relative effect for SCD [RR 0.81, 95% CI (0.67 to 0.98)], and all-cause mortality [RR 0.81, 95% CI (0.74 to 0.88)] (**Table 2**).

Angiotensin receptor blocker (ARB)/neprilysin inhibitor

One meta-analysis [23] estimated the effects of combined neprilysin renin-aldosterone system inhibition and reported a reduction in SCD and all-cause mortality. The finding was principally derived from one RCT (PARADIGM-HF) [5] that showed about 16% reduction of all-cause mortality in favor of sacubitril/valsartan (LCZ696 previously) compared to enalapril (an ACE-i). This mortality reduction was attributed to a decline on both SCD (20%) and pump failure deaths [55]. **Table 2** shows the relative effect for SCD [risk ratio (RR) 0.81, 95% confidence interval (CI) (0.69 to 0.95)], and all-cause mortality [RR 0.86, 95% CI (0.79 to 0.94)]. The moderate quality of the evidence for SCD outcome

For peer review only - http://bmjopen.bmj.com/site/about/guidelines.xhtml

was due to estimation from one single clinical trial and absence of data from other included studies. All-cause mortality was, however, rated as high with a possibility of downgrading in case of a detectable publication bias or unestablished class effect.

Ineffective Interventions

Angiotensin-converting enzyme inhibitors (ACE-i)

Although two systematic reviews [24 25], with an AMSTAR score of 2/11 and 3/11 respectively, reported a decline in total mortality and less progressive HF deaths, SCD events did not significantly decrease [OR 0.91, 95% CI (0.73 to 1.12)]. The quality of the evidence was rated as moderate because of the unclear or high risk of bias in included primary studies (**Table 2**).

Angiotensin receptor blockers (ARBs)

As shown in **Fig 2** and **Fig 3**, we updated a Cochrane review [27] by including more eligible primary studies such as SUPPORT trial.[56] Comparing ARBs to controls resulted in slightly different effect size estimation. Eventually, we did not combine the different control groups to account for heterogeneity. In stratified analyses, ARBs compared to placebo remained ineffective for all-cause mortality [RR 0.79, 95% CI (0.55 to 1.13)]. Similarly, ARBs, compared to ACE-I or in combination versus ACE-i alone, were not superior in all-cause mortality reduction [RR 0.87, 95% CI (0.56 to 1.36); RR 0.99, 95% CI (0.90 to 1.09), respectively] (**Fig 2**). The quality of the evidence is rated as low because of risk of bias, imprecision and inconsistency ($I^2 \approx 74$, P=0.010 for SCD outcome) (**Table 2**). Data were limited for studies reporting SCD, in particular those comparing ARBs versus placebo, or versus ACE-i (**Fig 2**). In addition, the funnel plot for all-cause mortality outcome showed no evidence of publication bias (**Fig 4**). No estimation of publication bias and no funnel plot was drawn for SCD as only five studies reported this outcome.

Statins

Published systematic reviews and meta-analyses about statins in HF were inconsistent [6 32-40] with a recent tendency toward inefficacy in total mortality and SCD prevention. The quality of the evidence was rated as moderate because of a likelihood of publication bias revealed on the most up-to-date systematic review [6] (**Table 2**).

<u>Unclear evidence of effectiveness</u>

The evidence of effectiveness of the drug interventions reported below was considered uncertain due either to conflicting or inconclusive evidence.

For peer review only - http://bmjopen.bmj.com/site/about/guidelines.xhtml

Amiodarone & anti-arrhythmic drugs (AADs)

Recently published systematic reviews[41 43] for amiodarone showed a significant reduction for SCD but not for all-cause mortality with less favorable net clinical benefits[42]. Other older reviews [44 45] of minor quality (AMSTAR of 3/11 and 5/11 cited respectively) reported a decline of both SCD and all-cause mortality. The quality of evidence for amiodarone was rated as low because of the unclear or high risk of bias and potential publication bias in primary studies (**Table 2**). No systematic review for AADs of other classes or drugs (other than amiodarone) were retrieved. Nevertheless, two narrative reviews [46 47] reported that class I anti-arrhythmics increased SCD and all-cause mortality. These narrative reviews called for caution regarding the mixed results of amiodarone and its adverse effects.

Omega-3 polyunsaturated fatty acids (PUFA) and fish oil supplementation

No systematic review was exclusively conducted in heart failure patients for this intervention. One primary study[57], known as GISSI-Prevenzione HF, recruited patients with chronic heart failure and reported a lower mortality events in the n-3 polyunsaturated fatty acids (PUFA) group compared to the placebo group. The authors reported an adjusted hazard ratio of 0.91 [95.5% CI 0.833 to 0.998], p=0.041). However, relative risk in our analysis remained statistically insignificant [RR 0.94, 95% CI (0.87 to 1.01), p=0.10] and [RR 0.94, 95% CI (0.81 to 1.09), p=0.42] for all-cause mortality and SCD, respectively. Our assessment of the quality of the evidence involving GISSI-Prevenzione HF was moderate because of an absence of data of any other relevant studies (**Table 2**). In addition, some recent systematic reviews [48-51] included patients regardless of their cardiovascular disease and concluded of no clear effect, insufficient evidence or no reduction on SCD and all-cause mortality outcomes. Meanwhile, some older studies [52 53] reported that omega-3 fatty acids and fish-oil supplements (other than α-linolenic acid [53]) reduced SCD and all-cause mortality.

Discussion

Our assessment of the effectiveness of drug interventions to prevent SCD in HFrEF patients indicated that beta-blockers (BBs), antialdosterone agents, as well as combined angiotensin receptor blocker (ARB)/neprilysin inhibitors were effective.

Previously reported meta-analyses and systematic reviews of RCTs [15-20] indicated that beta-blockers are effective in the prevention of SCD and all-cause mortality in HFrEF. However, although they were increasingly used as a usual “routine” care in the compared arms of the more recently published clinical trials[58], BBs stayed underused for long time and may still be [59]. Mineralocorticoid receptor antagonists or antialdosterone drugs have been reported effective in HFrEF by reducing SCD and all-

For peer review only - http://bmjopen.bmj.com/site/about/guidelines.xhtml

cause mortality [21 22 54 60]. Our summary of the findings and the consistency of the results support this claim with a high quality of evidence. Only one retrieved meta-analysis [23] supported the effectiveness of combined ARB/neprilysin inhibitor. The authors acknowledged the limitation of their meta-analysis, which was not based on a systematic review, but merely pooling three well-known trials published in high impact journals (i.e., IMPRESS [61], OVERTURE [62] and Paradigm-HF [5]). The quality of the evidence is, however, moderate for SCD and high for all-cause mortality, albeit our inability to assess any presence of a class effect or a potential publication bias.

We found that ACE-i showed a total mortality reduction in clinical trials and systematic reviews of heart failure patients [24 25]. However, our overview showed that ACE-i, surprisingly, did not significantly decrease SCD with a moderate quality of evidence.

In addition, we found that neither ARBs nor statins reduced SCD and/or all-cause mortality. Our findings for ARBs were in agreement with Jong and colleagues [31], Shibata et al. [28] and Dimopoulos et al. [30], but in contradiction to Lee VC et al. [29] and Rain & Rada's conclusions [26]. Our up-to-date meta-analysis for ARBs included only five primary studies, but large-scale trials, that reported SCD events. Eventually, we did not pool all the different comparators together but separately estimated the effect size for each group to account for the heterogeneity. Moreover, the addition of current trials such as SUPPORT [56], improved the statistical power of detecting an effect if existed and the summary statistic remained statistically insignificant (**Fig 2**). Of note, Jong and colleagues [31] attributed this inefficacy of ARBs in HF to the background treatment with ACE-i.

Within the current evidence, ARBs should not be seen as interchangeable with ACE-i, which also showed a neutral effect on SCD, without a proper reason. Therefore, in a high-risk SCD patient, another therapeutic strategy should be sought and an ARB/neprilysin inhibitor might be an alternative in patients similar to those of the Paradigm-HF trial[5].

The addition of statins to the therapy regimen of heart failure patients had no survival benefits. Actually, a recent systematic review and meta-analysis indicated that statins did not reduce SCD nor all-cause mortality [6]. Our current study reached the same conclusion with similar quality of evidence.

Our overview showed unclear evidence of effectiveness of Omega-3 polyunsaturated fatty acids (PUFA), fish oil supplementation and AADs. The latter intervention had an evidence originated from only narrative reviews, as we did not identify any systematic reviews. Also, only one n-3 PUFA clinical trial [57] was conducted in HF patients and reported a statistically significant mortality reduction; this result was not supported by other trials and recent systematic reviews [48-51], a finding that justified our

For peer review only - http://bmjopen.bmj.com/site/about/guidelines.xhtml

conclusion of unclear evidence. Moreover, no other data or systematic reviews conducted in HF were retrieved by our electronic and manual searches.

AADs are classified into four categories [46]: sodium channel blocking drugs (class I), BBs (class II), potassium channel blockers (class III), and calcium channel blockers (class IV). We found inconclusive evidence of effectiveness of all categories, with the exception of BBs. The evidence of effectiveness of class I, III and IV is inconclusive, neutral or even detrimental to patients as for class I AADs [46 47]. Amiodarones, which present class I, II, III, and IV effects, reported mixed results with potential SCD prevention with adverse effects [43] and potentially, but rare [63], life-threatening proarrhythmias [46].

Our overview has some limitations. First, we limited the scope of our study to drug treatment, thus excluding devices like implantable cardioverter defibrillators. We believe that non-drug devices should be tackled in future research. ESC Guideline (2016)[64] and others (e.g. www.uptodate.com) recommend the use of ICDs for only ≤ 35% HF patients and only after optimization of drug therapy. In fact, SCDs occur in both reduced and preserved heart failure. Our overview might help to optimize therapy as a first step before introducing ICDs, which applies to a limited HF subpopulation, regardless of costs. Second, we may have failed to include other drug interventions used in HFrEF. Such drug candidates include digoxin, I_f-channel blockers (ivabradine), hydralazine/isosorbide dinitrate, nitroglycerin, and phosphodiesterase 3 or 5 inhibitors. However, our overview included most commonly prescribed and evidence-based pharmacological therapy in HF as pre-specified in our published protocol[11]. Third, we did not use specific drug names in our literature search strategy, in order to avoid omitting a therapy that evaluated SCD and/or all-cause mortality prevention in heart failure patients. Fourth, we based our analyses on existing systematic reviews and meta-analyses and we updated only one meta-analysis. Consequently, we were unable to update the evidence for ACE-inhibitors. Furthermore, as indicated by the AMSTAR score, the methodological quality of some of the existing reviews was suboptimal. Fifth, we did not assess the safety of the evaluated drug interventions, nor the contra-indications for their prescription, drug-drug interactions, as well as treatment adherence. Indeed, we considered that these important aspects were out of the scope of our analysis. Sixth, we were unable to do a sensitivity analysis, initially suggested in our protocol, for ischaemic versus non-ischaemic heart failure due to limited data availability. Finally, a potential source of bias relates to authors of this overview being the authors of three of the included reviews [16 21 65]. However, the adopted methodology is in line with systematic reviews guidelines and ensured a

For peer review only - http://bmjopen.bmj.com/site/about/guidelines.xhtml

double check of data and methodological evaluation by at least two reviewers and a published protocol[11].

It is noteworthy that high quality evidence does not necessarily imply strong recommendations, and strong recommendations can arise from low quality evidence [66]. Therefore, when one intervention is graded high, it is not our intention to say that it is highly recommended, as we did not assess the level of recommendation in our study. In fact, level of recommendation not only depends on the strength of evidence, but also (among others) on values and preferences of patients, net benefits and cost-effectiveness of a particular intervention.

Implications for practice

Our study summarizes and synthesizes the effectiveness of most evidence-based drug interventions in HF patients with reduced ejection fraction (HFrEF) for SCD prevention. It classified drug interventions according to the current evidence of their effectiveness. This categorization could help health professionals and patients making evidence-based decisions based on updated knowledge, particularly whenever a high-risk SCD patient is identified. Currently, there is no an established strategy to deal with patients at high risk of SCD. In such patients, a particular attention should be considered and a careful selection of available therapeutic options is needed. Furthermore, there might be a shift towards an alternative therapeutic strategy based on SCD prevention-effective drugs in light of our findings.

Conclusion

Our overview indicates that only three drug interventions (BBs, anti-aldosterones, combined ARB/neprilysin inhibitors) significantly reduce SCD and improve overall survival among individuals with heart failure and reduced ejection fraction. On the other hand, there is no evidence of effectiveness of ARBs to reduce neither all-cause mortality nor SCD (with a low quality of evidence), and ACE-i do not significantly reduce SCD events. When the goal of drug therapy is to reduce SCD, especially in high-risk patients, our synthesis supports the use of the most effective regimen.

Author contribution

Design and conception: MA, FG, BB. (MA is the guarantor). Project administration: BB. Writing original draft: MA. Critical analysis: MA, SA, FG, BB. Data curation: MA, SA. Statistical analysis: MA. Proofread and approved the final draft: MA, SA, FG, BB.

For peer review only

For peer review only - http://bmjopen.bmj.com/site/about/guidelines.xhtml

Competing interests

We declare that some authors of this overview are also authors of some of the included reviews. However, at least two reviewers systematically checked and validated the extracted data including study qualities. We declare that no other competing interests exist.

Funding

MA, the corresponding author, received a financial grant (2014.0193) from The Federal Department of Economic Affairs, Education and Research (EAER), Switzerland, also from Département universitaire de médecine et santé communautaires (DUMSC), Lausanne university hospital (CHUV), Switzerland. The former funders had no role in study design or conception, study analysis, writing or preparation of the manuscript.

Ethics committee approval

This study did not need to ask for ethical approval and no direct patient involvement occurred.

Data sharing

All data are available in the manuscript and its supported files. Any more information can be requested from the corresponding author.

Acknowledgements

We thank Emilie Zuercher for her technical assistance.

Supporting materials

- S1 File: PRISMA checklist
- S2 File: Search strategy equations and result
- S3 Table: AMSTAR scores.
- S4 Table: Funding data of included reviews

Abbreviations

- ACE-i: angiotensin-converting enzyme inhibitor
- ACS: acute coronary syndrome
- ARBs: angiotensin receptor blockers

For peer review only - http://bmjopen.bmj.com/site/about/guidelines.xhtml

- ATMA: amiodarone trial meta-analysis
- BBs: beta-blockers
- CHD: coronary heart disease
- CHF: chronic heart disease
- GRADE: Grading of Recommendations, Assessment, Development and Evaluation
- HF: heart failure
- HFrEF: heart failure with reduced ejection fraction
- LVD: left ventricular dysfunction
- MI: Myocardial Infarction
- NA: not available or not applicable.
- NR: not reported
- NYHA: New York Heart Association class
- Post-MI: post myocardial infarction
- PUFAs: polyunsaturated fatty acids
- RAS: renin angiontensin system
- RCTs: randomized controlled trials
- SCD: sudden cardiac death
- SPAF-I: Stroke prevention Atrial Fibrillation Study
-
-

References

1. Thom T, Haase N, Rosamond W, et al. Heart disease and stroke statistics--2006 update: a report from the American Heart Association Statistics Committee and Stroke Statistics Subcommittee. *Circulation* 2006;113(6):e85-151. doi: 10.1161/circulationaha.105.171600 [published Online First: 2006/01/13]
2. Tomaselli GF, Zipes DP. What causes sudden death in heart failure? *Circulation research* 2004;95(8):754-63. doi: 10.1161/01.RES.0000145047.14691.db [published Online First: 2004/10/16]
3. Klein L, Hsia H. Sudden Cardiac Death in Heart Failure. *Cardiology Clinics* 2014;32(1):135-+. doi: 10.1016/j.ccl.2013.09.008
4. Ponikowski P, Voors AA, Anker SD, et al. 2016 ESC Guidelines for the diagnosis and treatment of acute and chronic heart failure: The Task Force for the diagnosis and treatment of acute and chronic heart failure of the European Society of Cardiology (ESC)Developed with the special contribution of the Heart Failure Association (HFA) of the ESC. *European heart journal* 2016;37(27):2129-200. doi: 10.1093/eurheartj/ehw128 [published Online First: 2016/05/22]
5. McMurray JJ, Packer M, Desai AS, et al. Angiotensin-neprilysin inhibition versus enalapril in heart failure. *The New England journal of medicine* 2014;371(11):993-1004. doi: 10.1056/NEJMoa1409077 [published Online First: 2014/09/02]

For peer review only - http://bmjopen.bmj.com/site/about/guidelines.xhtml

6. Al-Gobari M, Le HH, Fall M, et al. No benefits of statins for sudden cardiac death prevention in patients with heart failure and reduced ejection fraction: A meta-analysis of randomized controlled trials. *Plos One* 2017;12(2) doi: 10.1371/journal.pone.0171168
7. Lee E, Dobbins M, Decorby K, et al. An optimal search filter for retrieving systematic reviews and meta-analyses. *BMC medical research methodology* 2012;12:51. doi: 10.1186/1471-2288-12-51 [published Online First: 2012/04/20]
8. Wilczynski NL, Haynes RB. EMBASE search strategies achieved high sensitivity and specificity for retrieving methodologically sound systematic reviews. *Journal of clinical epidemiology* 2007;60(1):29-33. doi: 10.1016/j.jclinepi.2006.04.001 [published Online First: 2006/12/13]
9. Ouzzani M, Hammady H, Fedorowicz Z, et al. Rayyan-a web and mobile app for systematic reviews. *Systematic reviews* 2016;5(1):210. doi: 10.1186/s13643-016-0384-4 [published Online First: 2016/12/07]
10. Shea BJ, Grimshaw JM, Wells GA, et al. Development of AMSTAR: a measurement tool to assess the methodological quality of systematic reviews. *BMC medical research methodology* 2007;7:10. doi: 10.1186/1471-2288-7-10 [published Online First: 2007/02/17]
11. Al-Gobari M, Al-Aqeel S, Gueyffier F, et al. Effectiveness of drug interventions to prevent sudden cardiac death in patients with heart failure and reduced ejection fraction: an overview of systematic reviews. *PROSPERO 2017:CRD42017067442* 2017; Available from http://www.crd.york.ac.uk/PROSPERO/display_record.asp?ID=CRD42017067442
12. Kunz R, Burnand B, Schünemann HJ. Das GRADE-System. *Der Internist* 2008;49(6):673-80. doi: 10.1007/s00108-008-2141-9
13. Mantel N, Haenszel W. Statistical aspects of the analysis of data from retrospective studies of disease. *Journal of the National Cancer Institute* 1959;22(4):719-48. [published Online First: 1959/04/01]
14. Higgins JP, Thompson SG, Deeks JJ, et al. Measuring inconsistency in meta-analyses. *BMJ (Clinical research ed)* 2003;327(7414):557-60. doi: 10.1136/bmj.327.7414.557 [published Online First: 2003/09/06]
15. Chatterjee S, Biondi-Zoccai G, Abbate A, et al. Benefits of beta blockers in patients with heart failure and reduced ejection fraction: network meta-analysis. *BMJ (Clinical research ed)* 2013;346:no pagination.
16. Al-Gobari M, El Khatib C, Pillon F, et al. Beta-blockers for the prevention of sudden cardiac death in heart failure patients: a meta-analysis of randomized controlled trials. *Bmc Cardiovascular Disorders* 2013;13 doi: 10.1186/1471-2261-13-52
17. Brophy JM, Joseph L, Rouleau JL. Beta-blockers in congestive heart failure. A Bayesian meta-analysis. *Annals of internal medicine* 2001;134(7):550-60. [published Online First: 2001/04/03]
18. Lee S, Spencer A. Beta-blockers to reduce mortality in patients with systolic dysfunction: A meta-analysis. *Journal of Family Practice* 2001;50(6):499-504.
19. Bonet S, Agusti A, Arnau JM, et al. Beta-adrenergic blocking agents in heart failure: benefits of vasodilating and nonvasodilating agents according to patients' characteristics: a meta-analysis of clinical trials (Structured abstract). *Archives of Internal Medicine* 2000;160(5):621-27.
20. Heidenreich PA, Lee TT, Massie BM. Effect of beta-blockade on mortality in patients with heart failure: A meta-analysis of randomized clinical trials. *Journal of the American College of Cardiology* 1997;30(1):27-34.
21. Le HH, El-Khatib C, Mombled M, et al. Impact of Aldosterone Antagonists on Sudden Cardiac Death Prevention in Heart Failure and Post-Myocardial Infarction Patients: A Systematic Review and Meta-Analysis of Randomized Controlled Trials. *Plos One* 2016;11(2) doi: 10.1371/journal.pone.0145958
22. Wei J, Ni J, Huang D, et al. The effect of aldosterone antagonists for ventricular arrhythmia: a meta-analysis. *Clinical cardiology* 2010;33(9):572-7.
23. Solomon SD, Claggett B, McMurray JJ, et al. Combined neprilysin and renin-angiotensin system inhibition in heart failure with reduced ejection fraction: a meta-analysis. *European journal of heart failure* 2016;18(10):1238-43. doi: 10.1002/ejhf.603 [published Online First: 2016/07/02]
24. Garg R, Yusuf S. Overview of randomized trials of angiotensin-converting enzyme inhibitors on mortality and morbidity in patients with heart failure. Collaborative Group on ACE Inhibitor Trials. *Jama* 1995;273(18):1450-6. [published Online First: 1995/05/10]
25. Flather MD, Yusuf S, Kober L, et al. Long-term ACE-inhibitor therapy in patients with heart failure or left-ventricular dysfunction: a systematic overview of data from individual patients. ACE-Inhibitor Myocardial Infarction Collaborative Group. *Lancet* 2000;355(9215):1575-81. [published Online First: 2000/05/23]

For peer review only - http://bmjopen.bmj.com/site/about/guidelines.xhtml

26. Rain C, Rada G. Is there any difference between angiotensin converting enzyme inhibitors and angiotensin receptor blockers for heart failure? *Medwave* 2015;15 Suppl 1:e6177. doi: 10.5867/medwave.2015.06.6177 [published Online First: 2015/08/08]
27. Heran BS, Musini VM, Bassett K, et al. Angiotensin receptor blockers for heart failure. *The Cochrane database of systematic reviews* 2012(4):Cd003040. doi: 10.1002/14651858.CD003040.pub2 [published Online First: 2012/04/20]
28. Shibata MC, Tsuyuki RT, Wiebe N. The effects of angiotensin-receptor blockers on mortality and morbidity in heart failure: a systematic review. *International journal of clinical practice* 2008;62(9):1397-402. doi: 10.1111/j.1742-1241.2008.01806.x [published Online First: 2008/09/17]
29. Lee VC, Rhew DC, Dylan M, et al. Meta-analysis: angiotensin-receptor blockers in chronic heart failure and high-risk acute myocardial infarction. *Annals of internal medicine* 2004;141(9):693-704. [published Online First: 2004/11/03]
30. Dimopoulos K, Salukhe TV, Coats AJ, et al. Meta-analyses of mortality and morbidity effects of an angiotensin receptor blocker in patients with chronic heart failure already receiving an ACE inhibitor (alone or with a beta-blocker). *International journal of cardiology* 2004;93(2-3):105-11. doi: 10.1016/j.ijcard.2003.10.001 [published Online First: 2004/02/21]
31. Jong P, Demers C, McKelvie RS, et al. Angiotensin receptor blockers in heart failure: meta-analysis of randomized controlled trials. *J Am Coll Cardiol* 2002;39(3):463-70. [published Online First: 2002/02/02]
32. Rain C, Rada G. Are statins beneficial for chronic heart failure? First update. *Medwave* 2017;17(Suppl2):e6853. doi: 10.5867/medwave.2017.6853 [published Online First: 2017/04/30]
33. Osei Bonsu K, Reidpath DD, Kadirvelu A. Hydrophilic versus lipophilic statin therapy on outcomes in patients with heart failure: A systematic review and an adjusted indirect comparison meta-analysis of randomised trials. *European Heart Journal* 2015;36:546.
34. Wang JQ, Wu GR, Wang Z, et al. Long-term Clinical Outcomes of Statin Use for Chronic Heart Failure: A Meta-analysis of 15 Prospective Studies. *Heart Lung and Circulation* 2014;23(2):105-13.
35. Liu G, Zheng XX, Xu YL, et al. Effects of lipophilic statins for heart failure: a meta-analysis of 13 randomised controlled trials. *Heart, lung & circulation* 2014;23(10):970-7. doi: 10.1016/j.hlc.2014.05.005 [published Online First: 2014/06/24]
36. Zhang S, Zhang L, Sun A, et al. Efficacy of statin therapy in chronic systolic cardiac insufficiency: a meta-analysis. *European journal of internal medicine* 2011;22(5):478-84. doi: 10.1016/j.ejim.2011.06.003 [published Online First: 2011/09/20]
37. Xu M, Yuan G, Wei F. Effect of atorvastatin in patients with chronic heart failure - insights from randomized clinical trials. *Archives of medical science : AMS* 2010;6(6):866-73.
38. Lipinski MJ, Cauthen CA, Biondi-Zoccai GG, et al. Meta-analysis of randomized controlled trials of statins versus placebo in patients with heart failure. *The American journal of cardiology* 2009;104(12):1708-16. doi: 10.1016/j.amjcard.2009.07.055 [published Online First: 2009/12/08]
39. Levantesi G, Scarano M, Marfisi R, et al. Meta-analysis of effect of statin treatment on risk of sudden death. *The American journal of cardiology* 2007;100(11):1644-50. doi: 10.1016/j.amjcard.2007.07.015 [published Online First: 2007/11/27]
40. Rahimi K, Majoni W, Merhi A, et al. Effect of statins on ventricular tachyarrhythmia, cardiac arrest, and sudden cardiac death: A meta-analysis of published and unpublished evidence from randomized trials. *European Heart Journal* 2012;33(13):1571-81.
41. Claro JC, Candia R, Rada G, et al. Amiodarone versus other pharmacological interventions for prevention of sudden cardiac death. *Cochrane Database of Systematic Reviews* 2015(12) doi: 10.1002/14651858.CD008093.pub2
42. Santangeli P, Di Biase L, Burkhardt JD, et al. Examining the safety of amiodarone. *Expert Opinion on Drug Safety* 2012;11(2):191-214.
43. Piccini JP, Berger JS, O'Connor CM. Amiodarone for the prevention of sudden cardiac death: a meta-analysis of randomized controlled trials. *European Heart Journal* 2009;30(10):1245-53. doi: 10.1093/eurheartj/ehp100
44. Effect of prophylactic amiodarone on mortality after acute myocardial infarction and in congestive heart failure: meta-analysis of individual data from 6500 patients in randomised trials (Structured abstract). *Lancet* 1997;350(2):1417-24.

For peer review only - http://bmjopen.bmj.com/site/about/guidelines.xhtml

45. Sim I, McDonald KM, Lavori PW, et al. Quantitative overview of randomized trials of amiodarone to prevent sudden cardiac death. *Circulation* 1997;96(9):2823-29.
46. Das MK, Zipes DP. Antiarrhythmic and Nonantiarrhythmic Drugs for Sudden Cardiac Death Prevention. *Journal of Cardiovascular Pharmacology* 2010;55(5):438-49. doi: 10.1097/FJC.0b013e3181d64dde
47. Hilleman DE, Bauman AL. Role of antiarrhythmic therapy in patients at risk for sudden cardiac death: an evidence-based review. *Pharmacotherapy* 2001;21(5):556-75.
48. Rizos E, Ntzani E, Bika E, et al. Association between omega-3 fatty acid supplementation and risk of major cardiovascular disease events: a systematic review and meta-analysis (Structured abstract). *Jama* 2012;308(10):1024-33.
49. Kotwal S, Jun M, Sullivan D, et al. Omega 3 Fatty acids and cardiovascular outcomes: systematic review and meta-analysis. *Circulation Cardiovascular quality and outcomes* 2012;5(6):808-18. doi: 10.1161/circoutcomes.112.966168 [published Online First: 2012/11/01]
50. Kwak SM, Myung SK, Lee YJ, et al. Efficacy of omega-3 fatty acid supplements (eicosapentaenoic acid and docosahexaenoic acid) in the secondary prevention of cardiovascular disease: A meta-analysis of randomized, double-blind, placebo-controlled trials. *Archives of Internal Medicine* 2012;172(9):686-94.
51. Chen Q, Cheng L, Xiao T, et al. Effects of omega-3 fatty acid for sudden cardiac death prevention in patients with cardiovascular disease: a contemporary meta-analysis of randomized, controlled trials (Structured abstract). *Cardiovascular Drugs and Therapy* 2011;25(3):259-65.
52. Marik PE, Varon J. Omega-3 dietary supplements and the risk of cardiovascular events: a systematic review. *Clinical cardiology* 2009;32(7):365-72.
53. Wang C, Harris WS, Chung M, et al. n-3 Fatty acids from fish or fish-oil supplements, but not alpha-linolenic acid, benefit cardiovascular disease outcomes in primary- and secondary-prevention studies: a systematic review. *The American journal of clinical nutrition* 2006;84(1):5-17. [published Online First: 2006/07/11]
54. Bapoje SR, Bahia A, Hokanson JE, et al. Effects of Mineralocorticoid Receptor Antagonists on the Risk of Sudden Cardiac Death in Patients With Left Ventricular Systolic Dysfunction A Meta-analysis of Randomized Controlled Trials. *Circulation-Heart Failure* 2013;6(2):166-+. doi: 10.1161/circheartfailure.112.000003
55. Desai AS, McMurray JJ, Packer M, et al. Effect of the angiotensin-receptor-neprilysin inhibitor LCZ696 compared with enalapril on mode of death in heart failure patients. *European heart journal* 2015;36(30):1990-7. doi: 10.1093/eurheartj/ehv186 [published Online First: 2015/05/30]
56. Sakata Y, Shiba N, Takahashi J, et al. Clinical impacts of additive use of olmesartan in hypertensive patients with chronic heart failure: the supplemental benefit of an angiotensin receptor blocker in hypertensive patients with stable heart failure using olmesartan (SUPPORT) trial. *European heart journal* 2015;36(15):915-23. doi: 10.1093/eurheartj/ehu504 [published Online First: 2015/02/02]
57. Tavazzi L, Maggioni AP, Marchioli R, et al. Effect of n-3 polyunsaturated fatty acids in patients with chronic heart failure (the GISSI-HF trial): a randomised, double-blind, placebo-controlled trial. *Lancet* 2008;372(9645):1223-30. doi: 10.1016/s0140-6736(08)61239-8 [published Online First: 2008/09/02]
58. Shen L, Jhund PS, Petrie MC, et al. Declining Risk of Sudden Death in Heart Failure. *The New England journal of medicine* 2017;377(1):41-51. doi: 10.1056/NEJMoa1609758 [published Online First: 2017/07/06]
59. Busson A, Thilly N, Laborde-Casterot H, et al. Effectiveness of guideline-consistent heart failure drug prescriptions at hospital discharge on 1-year mortality: Results from the EPICAL2 cohort study. *European journal of internal medicine* 2018 doi: 10.1016/j.ejim.2017.12.005 [published Online First: 2018/01/07]
60. Anand K, Mooss AN, Mohiuddin SM. Aldosterone inhibition reduces the risk of sudden cardiac death in patients with heart failure. *Journal of the Renin-Angiotensin-Aldosterone System* 2006;7(1):15-19. doi: 10.3317/jraas.2006.001
61. Rouleau JL, Pfeffer MA, Stewart DJ, et al. Comparison of vasopeptidase inhibitor, omapatrilat, and lisinopril on exercise tolerance and morbidity in patients with heart failure: IMPRESS randomised trial. *Lancet* 2000;356(9230):615-20. [published Online First: 2000/09/01]
62. Packer M, Califf RM, Konstam MA, et al. Comparison of omapatrilat and enalapril in patients with chronic heart failure: the Omapatrilat Versus Enalapril Randomized Trial of Utility in Reducing Events (OVERTURE). *Circulation* 2002;106(8):920-6. [published Online First: 2002/08/21]
63. Doggrell SA. Amiodarone - Waxed and waned and waxed again. *Expert Opinion on Pharmacotherapy* 2001;2(11):1877-90.

For peer review only - http://bmjopen.bmj.com/site/about/guidelines.xhtml

64. Ponikowski P, Voors AA, Anker SD, et al. 2016 ESC Guidelines for the diagnosis and treatment of acute and chronic heart failure: The Task Force for the diagnosis and treatment of acute and chronic heart failure of the European Society of Cardiology (ESC). Developed with the special contribution of the Heart Failure Association (HFA) of the ESC. *European journal of heart failure* 2016;18(8):891-975. doi: 10.1002/ejhf.592 [published Online First: 2016/05/22]
65. Al-Gobari M, Le HH, Fall M, et al. No benefits of statins for sudden cardiac death prevention in patients with heart failure and reduced ejection fraction: A meta-analysis of randomized controlled trials. *PloS one* 2017;12(2):e0171168. doi: 10.1371/journal.pone.0171168 [published Online First: 2017/02/07]
66. Guyatt GH, Oxman AD, Vist GE, et al. GRADE: an emerging consensus on rating quality of evidence and strength of recommendations. *BMJ : British Medical Journal* 2008;336(7650):924-26. doi: 10.1136/bmj.39489.470347.AD

Legends of figures

Fig 1: flow chart for search result

Fig 2: Efficacy of angiotensin receptor blockers (ARBs) compared with placebo**,** angiotensin-converting enzyme inhibitor (ACE-i), or combined in heart failure and reduced ejection fraction (HFrEF) for the prevention of **all-cause mortality**.

Fig 3: Efficacy of angiotensin receptor blockers (ARBs) compared with placebo**,** angiotensin-converting enzyme inhibitor (ACE-i), or combined in heart failure and reduced ejection fraction (HFrEF) for the prevention of **sudden cardiac death (SCD).**

Fig 4: Funnel plot of SE (log odds ratio) by odds ratio to evaluate publication bias for the efficacy of angiotensin receptor blockers (ARBs) compared with control in heart failure and reduced ejection fraction (HFrEF) for the prevention of **all-cause mortality.**

For peer review only

For peer review only - http://bmjopen.bmj.com/site/about/guidelines.xhtml

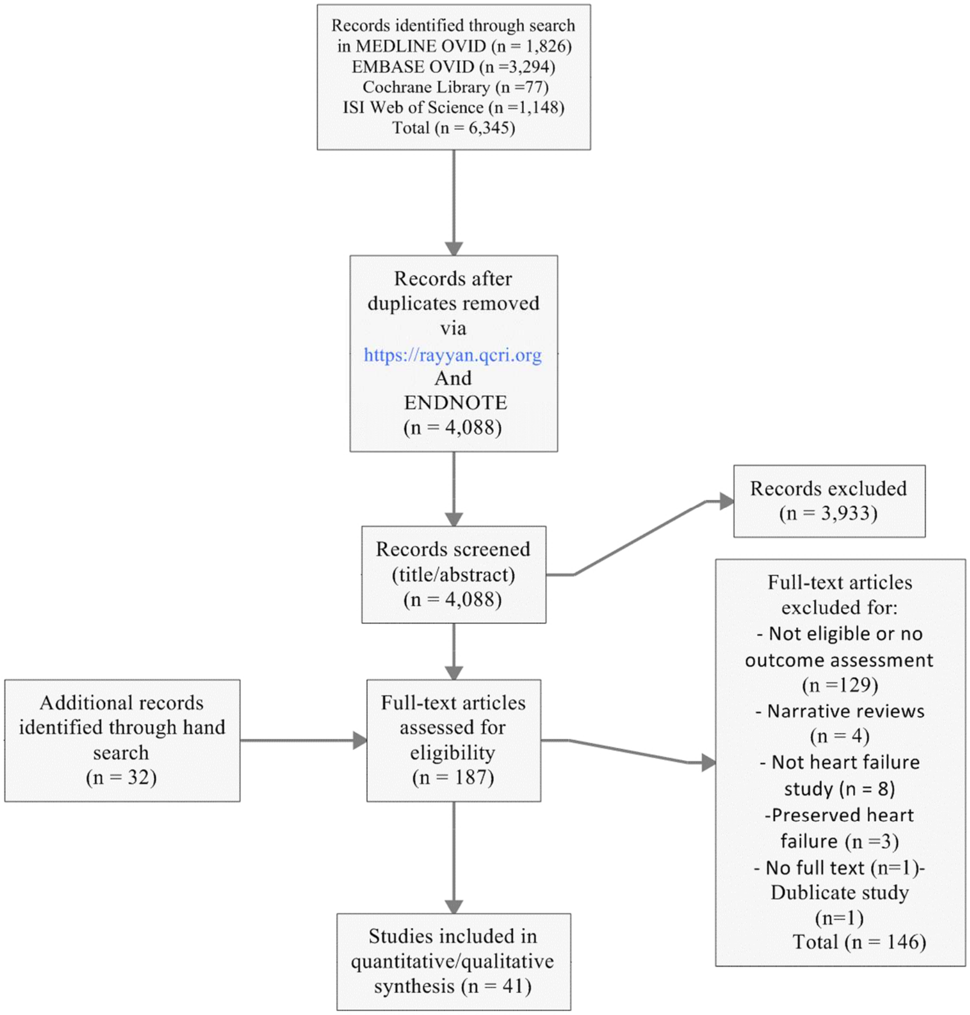

Fig 1: flow chart for search result.

103x108mm (300 x 300 DPI)

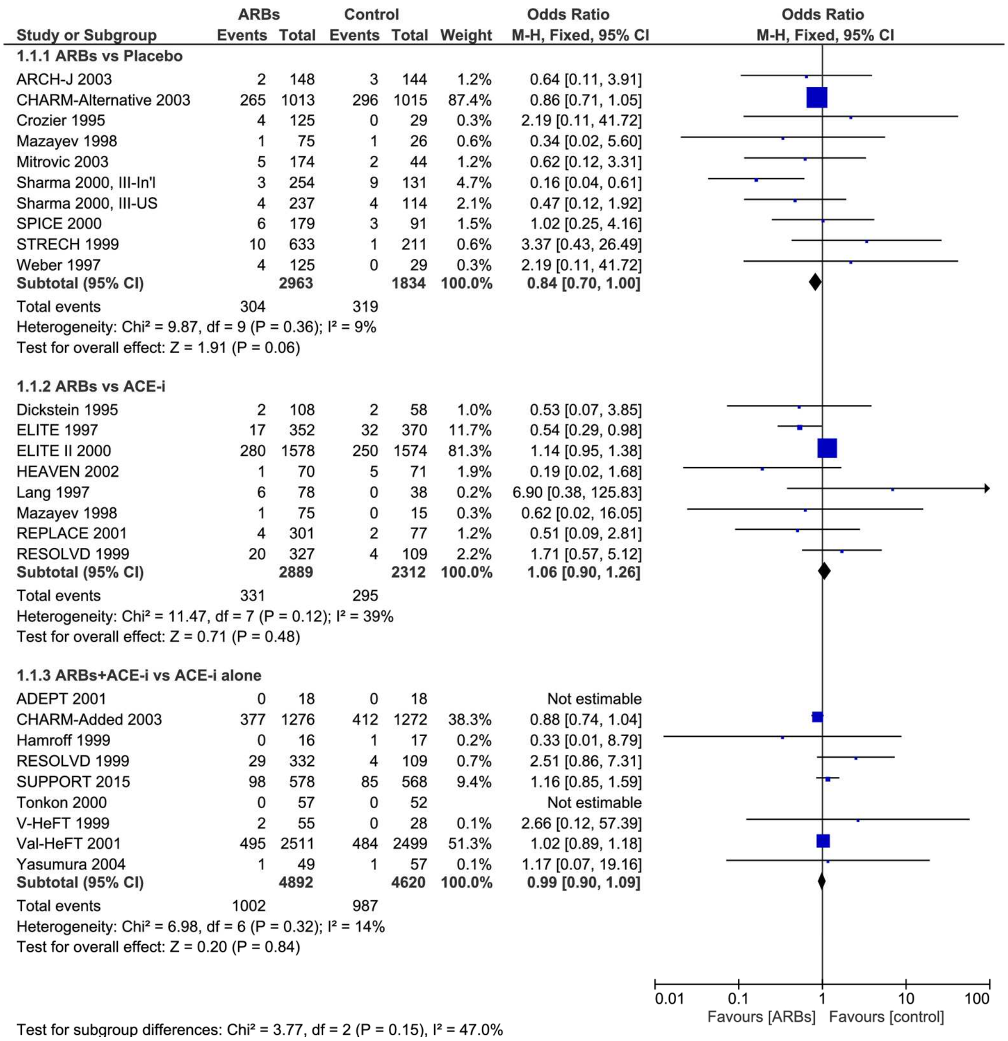

Fig 2: Efficacy of angiotensin receptor blockers (ARBs) compared with placebo, angiotensin-converting enzyme inhibitor (ACE-i), or combined in heart failure and reduced ejection fraction (HFrEF) for the prevention of all-cause mortality.

190x195mm (300 x 300 DPI)

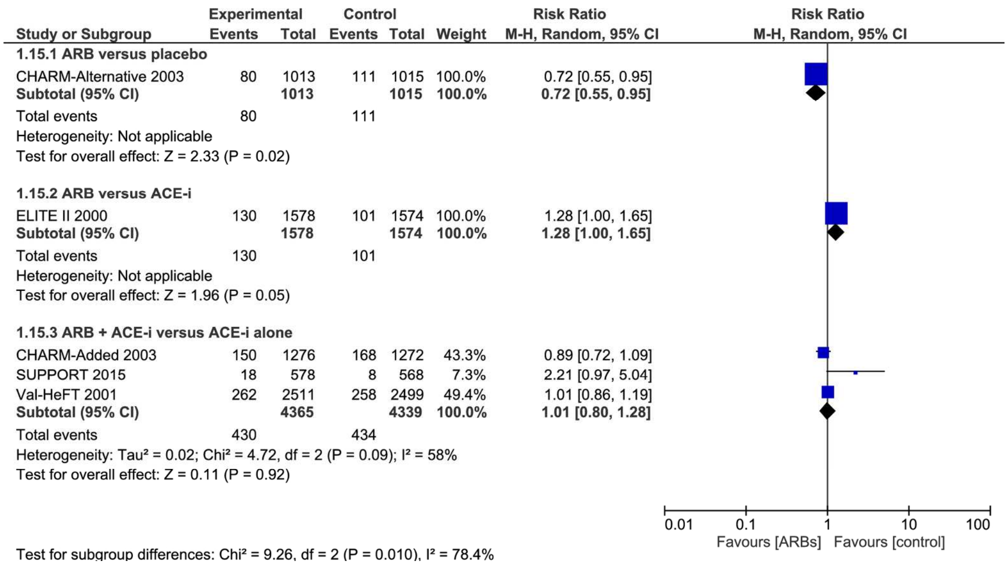

Fig 3: Efficacy of angiotensin receptor blockers (ARBs) compared with placebo, angiotensin-converting enzyme inhibitor (ACE-i), or combined in heart failure and reduced ejection fraction (HFrEF) for the prevention of sudden cardiac death (SCD).

190x105mm (300 x 300 DPI)

For peer review only

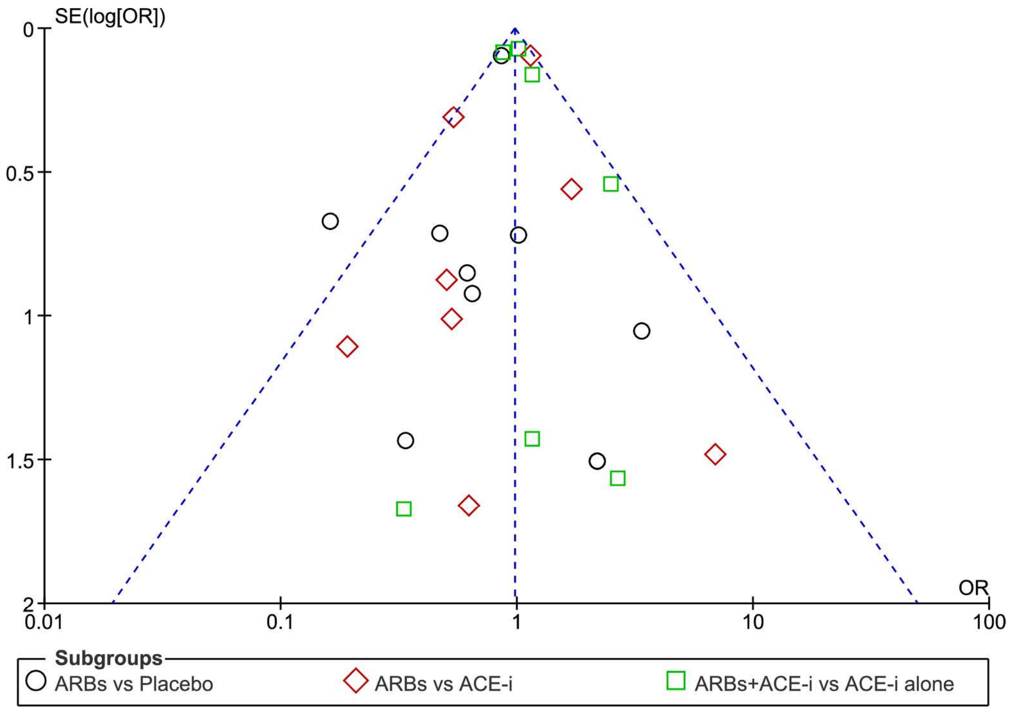

Fig 4: Funnel plot of SE (log odds ratio) by odds ratio to evaluate publication bias for the efficacy of angiotensin receptor blockers (ARBs) compared with control in heart failure and reduced ejection fraction (HFrEF) for the prevention of all-cause mortality.

190x133mm (300 x 300 DPI)

S1 File: PRISMA Checklist

Section/topic	#	Checklist item	Reported on page #
**TITLE**			
Title	1	Identify the report as a systematic review, meta-analysis, or both.	1
**ABSTRACT**			
Structured summary	2	Provide a structured summary including, as applicable: background; objectives; data sources; study eligibility criteria, participants, and interventions; study appraisal and synthesis methods; results; limitations; conclusions and implications of key findings; systematic review registration number.	2
**INTRODUCTION**			
Rationale	3	Describe the rationale for the review in the context of what is already known.	4
Objectives	4	Provide an explicit statement of questions being addressed with reference to participants, interventions, comparisons, outcomes, and study design (PICOS).	4
**METHODS**			
Protocol and registration	5	Indicate if a review protocol exists, if and where it can be accessed (e.g., Web address), and, if available, provide registration information including registration number.	4
Eligibility criteria	6	Specify study characteristics (e.g., PICOS, length of follow-up) and report characteristics (e.g., years considered, language, publication status) used as criteria for eligibility, giving rationale.	5
Information sources	7	Describe all information sources (e.g., databases with dates of coverage, contact with study authors to identify additional studies) in the search and date last searched.	5
Search	8	Present full electronic search strategy for at least one database, including any limits used, such that it could be repeated.	S2 File
Study selection	9	State the process for selecting studies (i.e., screening, eligibility, included in systematic review, and, if applicable, included in the meta-analysis).	5
Data collection process	10	Describe method of data extraction from reports (e.g., piloted forms, independently, in duplicate) and any processes for obtaining and confirming data from investigators.	5
Data items	11	List and define all variables for which data were sought (e.g., PICOS, funding sources) and any assumptions and simplifications made.	5
Risk of bias in individual studies	12	Describe methods used for assessing risk of bias of individual studies (including specification of whether this was done at the study or outcome level), and how this information is to be used in any data synthesis.	6
Summary measures	13	State the principal summary measures (e.g., risk ratio, difference in means).	6
Synthesis of results	14	Describe the methods of handling data and combining results of studies, if done, including measures of consistency (e.g., I^2) for each meta-analysis.	6

For peer review only

S1 File: PRISMA Checklist

Section/topic	#	Checklist item	Reported on page #
Risk of bias across studies	15	Specify any assessment of risk of bias that may affect the cumulative evidence (e.g., publication bias, selective reporting within studies).	6
Additional analyses	16	Describe methods of additional analyses (e.g., sensitivity or subgroup analyses, meta-regression), if done, indicating which were pre-specified.	6
**RESULTS**			
Study selection	17	Give numbers of studies screened, assessed for eligibility, and included in the review, with reasons for exclusions at each stage, ideally with a flow diagram.	7
Study characteristics	18	For each study, present characteristics for which data were extracted (e.g., study size, PICOS, follow-up period) and provide the citations.	Table 1
Risk of bias within studies	19	Present data on risk of bias of each study and, if available, any outcome level assessment (see item 12).	Tables 1&2 and S3
Results of individual studies	20	For all outcomes considered (benefits or harms), present, for each study: (a) simple summary data for each intervention group (b) effect estimates and confidence intervals, ideally with a forest plot.	8-10
Synthesis of results	21	Present the main results of the review. If meta-analyses are done, include for each, confidence intervals and measures of consistency.	8-10
Risk of bias across studies	22	Present results of any assessment of risk of bias across studies (see Item 15).	S3 Table & Fig 4
Additional analysis	23	Give results of additional analyses, if done (e.g., sensitivity or subgroup analyses, meta-regression [see Item 16]).	9 (Fig 2-3)
**DISCUSSION**			
Summary of evidence	24	Summarize the main findings including the strength of evidence for each main outcome; consider their relevance to key groups (e.g., healthcare providers, users, and policy makers).	10-11
Limitations	25	Discuss limitations at study and outcome level (e.g., risk of bias), and at review-level (e.g., incomplete retrieval of identified research, reporting bias).	12
Conclusions	26	Provide a general interpretation of the results in the context of other evidence, and implications for future research.	13
**FUNDING**			
Funding	27	Describe sources of funding for the systematic review and other support (e.g., supply of data); role of funders for the systematic review.	14

From: Moher D, Liberati A, Tetzlaff J, Altman DG, The PRISMA Group (2009). Preferred Reporting Items for Systematic Reviews and Meta-Analyses: The PRISMA Statement. PLoS Med 6(7): e1000097. doi:10.1371/journal.pmed1000097. For more information, visit: **www.prisma-statement.org**.

For peer review only - http://bmjopen.bmj.com/site/about/guidelines.xhtml

Database(s): **Ovid MEDLINE(R) Epub Ahead of Print, In-Process & Other Non-Indexed Citations, Ovid MEDLINE(R) Daily and Ovid MEDLINE(R)** 1946 to 25/05/2017
Search Strategy: 24.05.2017

#	Searches	Results
1	exp heart failure/ or (((heart or cardia* or myocardial) adj3 failure*) or (((heart or cardia*) adj3 decompensation*) or ((heart or cardia*) adj3 insufficienc*))).mp.	189098
2	review.ab.	1050955
3	review.pt.	2299071
4	meta-analysis.ab.	82199
5	meta-analysis.pt.	80375
6	or/2-5	2743700
7	letter.pt.	971739
8	comment.pt.	690989
9	editorial.pt.	439919
10	or/7-9	1585046
11	6 not 10	2708683
12	1 and 11	39715
13	(sudden adj3 (cardiac or death*)).ti,ab,kw.	42355
14	12 and 13	1826

For peer review only

Database(s): **Embase** 1974 to 2017 May 23rd
Search Strategy:

#	Searches	Results
1	exp heart failure/ or (((heart or cardia* or myocardial) adj3 failure*) or (((heart or cardia*) adj3 decompensation*) or ((heart or cardia*) adj3 insufficienc*))).mp.	444143
2	meta-analy:.mp.	202659
3	search:.tw.	417814
4	review.pt.	2246810
5	or/2-4	2642821
6	exp Sudden Cardiac Death/ or Sudden Death/ or exp heart arrest/	107134
7	5 and 6	14446
8	1 and 7	9747
9	(sudden adj3 (cardiac or death*)).mp.	76511
10	limit 9 to abstracts	58853
11	8 and 10	3294

For peer review only - http://bmjopen.bmj.com/site/about/guidelines.xhtml

S3 Table: AMSTAR scores for included studies

For peer review only

Author (year); country; reference	Q 1	Q 2	Q 3	Q 4	Q 5	Q 6	Q 7	Q 8	Q 9	Q 10	Q 11	AMSTAR score
Al-Gobari M., et al. (2013), France [14]	No	No	Yes	No	No	Yes	Yes	Yes	Yes	Yes	No	6
Chatterjee S., et al. (2013); USA [13]	No	Yes	Yes	Yes	No	Yes	Yes	Yes	Yes	Yes	No	8
Brophy JM. Et al., (2001); Canada [15]	No	No	Yes	No	No	Yes	No	No	Yes	Yes	No	4
Lee S. et al., (2001), USA [16]	No	No	Yes	Yes	No	Yes	No	No	Yes	No	No	4
Bonet S., et al. (2000), USA [17]	No	Yes	Yes	No	No	Yes	No	No	Yes	No	No	4
Heidenreich P.A., et al. (1997); USA [18]	No	Yes	No	Yes	Yes	Yes	No	No	Yes	No	No	5
Le HH. et al., (2016); France [19]	No	Yes	Yes	No	No	Yes	Yes	Yes	Yes	Yes	No	7
Bapoje SR. et al. (2013); USA [52]	No	Yes	Yes	Yes	No	Yes	Yes	Yes	Yes	Yes	No	8
Wei J., et al. (2010); China [20]	No	Yes	Yes	Yes	No	Yes	No	No	Yes	No	No	5
Solomon SD., et al. (2016); USA [21]	No	Yes	Yes	No	No	Yes	Yes	Yes	yes	No	Yes	7
Flather MD et al., (2000); Canada [23]												NA*
Garg R. et al., (1995); Canada [22]	No	Yes	No	No	No	No	No	No	Yes	No	No	2
Rain & Rada, (2015) ; Chile [24*]												NA*
Heran et al., (2012) ; Canada [25]	Yes	Yes	Yes	No	Yes	Yes	Yes	Yes	Yes	Yes	Yes	10
Shibata et al., (2008) ; Canada [26]	No	Yes	No	Yes	No	Yes	No	No	Yes	No	No	4
Lee et al., (2004) ; USA [27]	No	Yes	Yes	Yes	No	Yes	Yes	No	Yes	Yes	No	7
Dimopoulos et al., (2004) ; UK [28]	No	Yes	No	No	No	Yes	No	No	Yes	No	No	3
Jong P., et al. (2002), Canada [29]	Yes	Yes	Yes	No	No	Yes	Yes	Yes	Yes	Yes	No	8
Rain & Rada, (2017); Chile [30*]												NA*
Al-Gobari M., et al. (2017), Switzerland [5]	No	Yes	Yes	No	No	Yes	Yes	Yes	Yes	Yes	No	7
Bonsu KO, et al. (2015), Malaysia [31]	Yes	Yes	Yes	No	No	Yes	Yes	Yes	Yes	Yes	No	8

Wang JQ., et al. (2014); China [32]	No	Yes	Yes	No	No	Yes	Yes	No	No	Yes	No	5
Liu G. et al. (2014); China [33]	No	Yes	Yes	No	No	Yes	Yes	Yes	Yes	Yes	No	7
Rahimi K. et al. (2012); UK [38]	No	Yes	Yes	No	No	Yes	Yes	Yes	Yes	No	No	6
Zhang S. et al. (2011); China [34]	No	Yes	Yes	No	No	Yes	Yes	Yes	Yes	Yes	No	7
Xu M. et al. (2010); China [35]	No	Yes	Yes	Yes	No	Yes	No	No	Yes	Yes	No	6
Lipinski MJ. (2009); USA [36]	No	Yes	Yes	No	No	Yes	Yes	Yes	Yes	Yes	No	7
Levantesi G. et al. (2007); Italy [37]	No	Yes	No	No	No	Yes	No	No	Yes	No	No	3
Claro JC., et al. (2015), Chile [39]	Yes	Yes	Yes	Yes	Yes	Yes	Yes	Yes	Yes	Yes	No	10
Santangeli P. et al. (2012); USA [40]	No	Yes	Yes	Yes	No	Yes	No	No	Yes	No	No	5
Piccini JP., et al. (2009); USA [41]	No	Yes	Yes	No	No	Yes	Yes	Yes	Yes	Yes	No	7
ATMA Investigators (1997) [42]												NA*
Sim I. et al., (1997); USA [43]	No	Yes	Yes	Yes	No	Yes	No	No	Yes	No	No	5
Das MK. et al., (2010): USA [44]*												NA*
Hilleman DE. et al., (2001); USA [45]*												NA*
Rizos EC., et al. (2012); Greece [46]	No	Yes	Yes	No	No	Yes	Yes	Yes	Yes	Yes	Yes	8
Kotwal S. et al. (2012); Australia [47]	No	Yes	Yes	No	No	Yes	Yes	Yes	Yes	Yes	No	7
Kwak SM., et al. (2012); Korea [48]	No	Yes	Yes	No	No	Yes	Yes	Yes	Yes	Yes	Yes	8
Chen Q., et al. (2011); China [49]	No	Yes	Yes	No	No	Yes	Yes	Yes	Yes	Yes	No	7
Marik PE., et al. (2009); USA [50]	No	Yes	Yes	No	No	Yes	No	No	Yes	No	No	4
Wang C., et al. (2006); USA [51]	No	No	Yes	Yes	No	Yes	Yes	Yes	Yes	No	No	6

For peer review only

Q 1: Was an 'a priori' design provided?

Q 2: Was there duplicate study selection and data extraction?

Q 3: Was a comprehensive literature search performed?

Q 4: Was the status of publication (i.e. grey literature) used as an inclusion criterion?

Q 5: Was a list of studies (included and excluded) provided?

Q 6: Were the characteristics of the included studies provided?

Q 7: Was the scientific quality of the included studies assessed and documented?

Q 8: Was the scientific quality of the included studies used appropriately in formulating conclusions?

Q 9: Were the methods used to combine the findings of studies appropriate?

Q 10: Was the likelihood of publication bias assessed?

Q 11: Was the conflict of interest included?

* narrative review, overview or individual patient data meta-analysis (not applicable)

For peer review only

For peer review only

S4 Table: Funding data of included reviews of heart failure patients

Author (year); country; reference	Review type	Intervention/ Comparator	Funding
Al-Gobari M., et al. (2013), France [14]	Systematic review & meta-analysis	Beta-blockers /Placebo; "usual care"	YEMEN LNG company: Private company but non-drug-industry., UMR 5558, BEBL.
Chatterjee S., et al. (2013); USA [13]	Systematic review & meta-analysis	Beta-blockers / Placebo; beta-blocker; "usual care"	None
Brophy JM. Et al., (2001); Canada [15]	Meta-analysis	Beta-blockers / Placebo; "usual care"	Les Fonds de la Recherche en Santé du Québec. Government (non-industry)
Lee S. et al., (2001), USA [16]	systematic review & meta-analysis	Beta-blockers / Placebo	No data reported
Bonet S., et al. (2000), USA [17]	Meta-analysis	Beta-blockers / Placebo ; "usual care"	No data reported
Heidenreich P.A., et al. (1997); USA [18]	Meta-analysis	Beta-blockers / Placebo; "usual care"	No data reported
Le HH. et al., (2016); France [19]	Systematic review & meta-analysis	Anti-aldosterone / Placebo; "usual care"	Non-industry (Academic and Government)
Bapoje SR. et al. (2013); USA [52]	SR and MA	Anti-aldosterone / Placebo; "usual care"	Not reported for included studies. No external fund for the review.
Wei J., et al. (2010); China [20]	Meta-analysis	Anti-aldosterone / Placebo; "usual care"	No data reported
Solomon SD., et al. (2016); USA [21]	Meta-analysis	Sacubitril; valsartan / ACE-i	Data reported for included studies: industry. No funding existed for the review itself.
Flather MD et al., (2000); Canada [23]	Systematic review	ACE-i / Placebo	Industry support: Medical Research Council of Canada and support from Merck Frosst Canada, Merck Sharpe and Dohme, USA, Bristol Myers, Squibb, USA, Zeneca, UK, and Hoechst Marion Roussel, Frankfurt, Germany.
Garg R. et al., (1995); Canada [22]	Systematic review & meta-analysis	ACE-i / Placebo	No data reported
Rain & Rada, (2015) ; Chile [24]	Systematic review	ARB/ ACE-i	No data reported
Heran et al., (2012) ; Canada [25]	Systematic review & meta-analysis (Cochrane)	ARB (or ARB + ACE-i) / Placebo ; ACE-i	Included studies: 23 (96%) industry-sponsored and remaining unknown. Review. External: NIHR, UK Cochrane (Non-industry)
Shibata et al., (2008) ; Canada [26]	Systematic review & meta-analysis	ARB/ Placebo ; ACE-i	No funding exists. Not reported for included studies.
Lee et al., (2004) ; USA [27]	Meta-analysis	ARB/ Placebo ; ACE-i	Industry support: Stock ownership or options (other than mutual funds): D.C. Rhew (Merck, Pfizer); Grants received: Zynx Health Incorporated has received grants from AstraZeneca, Aventis, Bristol-Myers Squibb, Merck, Novartis, and Pfizer.
Dimopoulos et al., (2004) ; UK [28]	Meta-analysis	ARB/ Placebo ; ACE-i	No data reported
Jong P., et al. (2002), Canada [29]	Systematic review & meta-analysis	ARB (or ARB + ACE-i) / Placebo ; ACE-i	No data reported
Rain & Rada, (2017); Chile [30]	systematic review	Statins / Placebo ; "usual care"	No data reported
Al-Gobari M., et al. (2017), Switzerland [5]	Systematic review & meta-analysis	Statins / Placebo ; "usual care"	Yes for the review: non-industry (Governments). Not reported for included studies.

For peer review only

Bonsu KO, et al. (2015), Malaysia [31]	Meta-analysis	Statins / Placebo ; “usual care”	No data reported
Wang JQ., et al. (2014); China [32]	Meta-analysis	Statins / Placebo ; “usual care”	None (reported) but not for included studies.
Liu G. et al. (2014); China [33]	Meta-analysis	Statins / Placebo ; “usual care”	Non-industry-sponsored but no data for included studies.
Rahimi K. et al. (2012); UK [38]	Meta-analysis	Statins / Placebo ; “usual care”	Funding for included studies reported. Some are industry. No funding for the review itself from industry but from Academy for some authors.
Zhang S. et al. (2011); China [34]	Meta-analysis	Statins / Placebo ; “usual care”	Non-industry-sponsored but no data for included studies. Academic, doctoral program…etc
Xu M. et al. (2010); China [35]	Meta-analysis	Statins / Placebo ; “usual care”	No data reported
Lipinski MJ. (2009); USA [36]	Meta-analysis	Statins / Placebo ; “usual care”	No data reported
Levantesi G. et al. (2007); Italy [37]	Meta-analysis	Statins / Placebo ; “usual care”	No data reported
Claro JC., et al. (2015), Chile [39]	Systematic review & meta-analysis (Cochrane)	Amiodarone / Placebo; “usual care”	Funding for the review: Government (non-industry). Not reported for included studies.
Santangeli P. et al. (2012); USA [40]	systematic review	Amiodarone / Placebo	No data reported
Piccini JP., et al. (2009); USA [41]	Meta-analysis	Amiodarone / Placebo ; “usual care”	No external fund for the review. No data reported for included studies.
ATMA Investigators (1997) [42]	Meta-analysis	Amiodarone / Placebo ; “usual care”	No data reported
Sim I. et al., (1997); USA [43]	Meta-analysis	Amiodarone / Placebo ; “usual care”	No data reported
Das MK. et al., (2010): USA [44]	Narrative Review	Anti-arrhythmics / Placebo ; “usual care”	No data reported
Hilleman DE. et al., (2001); USA [45]	Narrative Review	Anti-arrhythmics / Placebo ; “usual care”	No data reported
Rizos EC., et al. (2012); Greece [46]	systematic review & meta-analysis	Omega 3 Fatty acids / Placebo ; “usual care”	Funding of included studies is reported. Conflict of interest too (for one author): industry sponsorship not related to those who manufacture or market Omega-3 suppl.
Kotwal S. et al. (2012); Australia [47]	systematic review & meta-analysis	Omega 3 Fatty acids / Placebo ; “usual care”	Reported but not for included studies. Non-industry.
Kwak SM., et al. (2012); Korea [48]	Meta-analysis	Omega 3 Fatty acids / Placebo ; “usual care”	All included trials sponsored by industry but not reported for the review itself.
Chen Q., et al. (2011); China [49]	Meta-analysis	Omega 3 Fatty acids / Placebo ; “usual care”	No data reported
Marik PE., et al. (2009); USA [50]	systematic review	Omega 3 dietary supplements / Placebo; olive oil ; corn oil, sunflower oil ; “usual care”	No data reported
Wang C., et al. (2006); USA [51]	systematic review	n-3 Fatty acids / Placebo/olive oil ; corn oil, sunflower oil ; “usual care”	Internal by saying who obtained funds but no specific source. No data for included studies.

For peer review only

Effectiveness of drug interventions to prevent sudden cardiac death in patients with heart failure and reduced ejection fraction: an overview of systematic reviews

Muaamar Al-Gobari[1*], Sinaa Al-Aqeel[2], François Gueyffier[3], Bernard Burnand[1]

[1] Institute of social & preventive medicine (IUMSP) and Cochrane Switzerland, Lausanne University Hospital (CHUV), Lausanne, Switzerland

[2] Clinical Pharmacy Department, College of Pharmacy, King Saud University, Riyadh, Kingdom of Saudi Arabia.

[3] Laboratoire de Biologie et Biométrie Evolutive-Equipe Modélisation des Effets Thérapeutiques, UMR 5558 Université Claude Bernard Lyon1, Lyon, France

*** Corresponding Author Information:**

Institute of social & preventive medicine (IUMSP)

Biopôle 2, SV-A

Route de la Corniche 10

CH-1010 Lausanne, Switzerland

Tel. +41 21 314 51 45

E-mail: muaamar.algobari@gmail.com

For peer review only

For peer review only - http://bmjopen.bmj.com/site/about/guidelines.xhtml

Abstract

Objectives

To summarize and synthesize the current evidence regarding the effectiveness of drug interventions to prevent sudden cardiac death (SCD) and all-cause mortality in heart failure with reduced ejection fraction (HFrEF) patients.

Design

Overview of systematic reviews.

Data sources

Medline, Embase, ISI Web of Science and Cochrane Library from inception to May 2017; manual search of references of included studies for potentially relevant reviews.

Eligibility criteria for study selection

We reviewed the effectiveness of drug interventions for SCD and all-cause mortality prevention in HFrEF patients. We included overviews, systematic reviews and meta-analyses of randomized controlled trials of beta-blockers, angiotensin-converting enzyme inhibitors (ACE-i), angiotensin receptor blockers (ARBs), anti-aldosterones or mineralocorticoid-receptor antagonists, amiodarone, other antiarrhythmic drugs, combined angiotensin receptor blocker (ARB)/neprilysin inhibitors, statins and fish oil supplementation.

Review methods

Two independent reviewers extracted data and assessed the methodological quality of the reviews and the quality of evidence for the primary studies for each drug intervention, using AMSTAR and GRADE respectively.

Results

We identified 41 reviews. Beta-blockers, anti-aldosterones, and combined ARB/neprilysin inhibitors appeared effective to prevent SCD and all-cause mortality. ACE-i significantly reduced all-cause mortality but not SCD events. ARBs and statins were ineffective where antiarrhythmic drugs and omega-3 fatty acids had unclear evidence of effectiveness for prevention of SCD and all-cause mortality.

For peer review only - http://bmjopen.bmj.com/site/about/guidelines.xhtml

Conclusions

This comprehensive overview of systematic reviews confirms that beta-blockers, anti-aldosterone agents and combined ARB/neprilysin inhibitors are effective on SCD prevention but not ACE-i or ARBs. In patients with high risk of SCD, an alternative therapeutic strategy should be explored in future research.

Systematic Review Registration: PROSPERO 2017: CRD42017067442

Key Words

cardiac failure, sudden death, umbrella review, treatment, pharmacotherapy, meta-analysis, guidelines, evidence-based medicine

Strengths and limitations of this study

- A major strength of our study is that it summarizes and synthesizes the effectiveness of most evidence-based drug interventions in heart failure patients with reduced ejection fraction for sudden cardiac death (SCD) prevention and classified drug interventions according to the current evidence of their effectiveness.
- Our study used data from published studies and no data from unpublished studies.
- Our study reviews most heart failure drugs on the prevention of SCD and all-cause mortality but limited in scope for not including some drugs such as digoxin, ivabradine, and non-drug interventions/devices such as implantable cardioverter defibrillators.

For peer review only

Introduction

Heart failure (HF) morbidity and mortality constitute an important burden for patients and for the healthcare systems in both developed and developing countries [1]. HF patients are frequently hospitalized and not only have a high mortality risk from a bad prognosis, but also unexpectedly, termed sudden cardiac death (SCD). In people diagnosed with HF, SCD occurs at 6–9 times the rate of the general population. Almost 20% and 80% of patients die within one year and eight years of initial diagnosis, respectively [1 2]. Risk factors of SCD were reported to be similar to cardiovascular diseases. However, the most studied and proven predictor of SCD in HF patients is left ventricular ejection fraction (LVEF)[3]. Potential drug interventions in heart failure with reduced ejection fraction (HFrEF) patients include beta-blockers (BBs), angiotensin-converting enzyme inhibitors (ACE-i), angiotensin receptor blockers (ARBs), anti-aldosterones or mineralocorticoid-receptor antagonists, amiodarone, other antiarrhythmic agents, combined angiotensin receptor blocker/neprilysin inhibitors, statins and fish oil supplementation [4]. Some of these interventions aimed at improving survival and reducing total mortality and SCD in HF. For instance, a newly licensed drug (Sacubitril/valsartan) in Paradigm-HF trial showed around 20% SCD reduction compared to enalapril [5].

Nevertheless, optimal strategies for SCD prevention in HF are warranted if we take into account the high portion of mortality that still occurs in this population. Had a practitioner identified a patient with high risk of SCD, it would be important to know which drug is effective or not in SCD prevention other than non-drug interventions such as implantable cardioverter defibrillators (ICDs). However, the large amount of information and the multiple and sometimes discordant systematic reviews on drug interventions could be misleading [6].

Therefore, it is vital to identify the pharmacological agents that confer the greatest benefit in SCD risk reduction particularly in high-risk patients and if any optimization of therapeutic strategies to those patients is possible accordingly. Thus, we decided to conduct an overview of systematic reviews to summarize and synthesize the available evidence about the effectiveness of drug interventions in the prevention of SCD in HFrEF, and categorized the evidence into effective, ineffective and unclear evidence of effectiveness.

For peer review only - http://bmjopen.bmj.com/site/about/guidelines.xhtml

Methods

We developed an a priori protocol for this review according to the Preferred Reporting Items for Systematic Reviews and Meta-Analyses (PRISMA) statement (**S1 File**) and registered it in the PROSPERO International prospective register of systematic reviews (CRD42017067442).

Data sources and search strategy

Using the Ovid online interface, we searched Medline (up to May 24, 2017), Embase (up to May 23, 2017), ISI web of science and the Cochrane library (up to May 24, 2017). We identified overviews, systematic reviews and meta-analyses of randomized clinical trials by means of a search strategy (available on **S2 File**). The search strategy was composed of a filter [7 8], a mixture of medical subject heading terms [Mesh and EMTREE in Medline and Embase respectively], text words as well as a truncation when possible without any language or publication date restriction. We did not search conference proceedings nor the grey literature. Reference lists of the included reviews were manually checked for any additional eligible studies. We contacted corresponding reviews' and primary studies' authors to seek for relevant unreported data. If judged necessary, we intended to update the included reviews by searching primary studies published after the systematic review publication date. Apart from authors' expertise in the field, we decided to update if the most up-to-date review of a drug intervention was published more than 5 years ago and/or new clinical trials are not integrated into the evidence.

Selection criteria and data abstraction

Studies were eligible if they were overviews, systematic reviews and meta-analyses of randomized clinical trials that evaluated the effectiveness of drug interventions in HFrEF patients. Reviews were included if they examined the effectiveness of the following drugs: BBs, ACE-i, ARBs, anti-aldosterones or mineralocorticoid-receptor antagonists, amiodarone, antiarrythmics, combined ARB/neprilysin inhibitors, statins and fish oil supplementation. The selected reviews should have contained at least one of the aforementioned HF therapy and had evaluated SCD and/or all-cause mortality prevention as outcomes. We used Endnote and Rayyan[9] to remove duplicates during the selection based on titles and abstracts, and full-text screening.

The abstracted data included eligibility criteria, population type, ejection fraction, study design (including intervention and comparator arms), follow-up duration, and authors' evaluation of outcomes. Two reviewers (MA&SA) independently abstracted data. We resolved discrepancies by consensus or by adding a third reviewer's judgment, when necessary.

For peer review only - http://bmjopen.bmj.com/site/about/guidelines.xhtml

Quality assessment of the included reviews

Methodological quality of the included reviews

Two authors (MA&SA) independently used the AMSTAR measurement tool to assess systematic reviews included in our overview. The AMSTAR checklist comprises eleven questions (**S3 table**) and each question accounted for one score point [10]. The answer of "yes" gave a score of 1 and zero otherwise. This increasingly adopted tool was used at the data collection step as stipulated in the overview protocol[11]. If the authors of included reviews failed to publish their protocol, we deducted a score of one. In addition, we scored "yes" if the authors mentioned that two reviewers were involved in the study screening, selection or data extraction.

Quality of evidence in the included reviews

Two authors (MA&SA) independently used the GRADE (Grading of Recommendations, Assessment, Development and Evaluation) approach [12] to assess the quality of evidence of each intervention. GRADE is a widely accepted tool that allows the assessment of five key elements: risk of bias, inconsistency, indirectness, imprecision, and publication bias. GRADE categorizes the quality of evidence into four levels: high, moderate, low and very low. In the presence of a high risk of bias, the quality of the evidence is downgraded from high to moderate and so on. We also reported the GRADE assessments reported by the authors of the included reviews, or assessed them otherwise. Moreover, we did not reassess the risk of bias at primary study level if authors of included reviews had sufficiently assessed their quality. In the case of the updated review of angiotensin receptor blockers, however, we assessed the quality of newly added randomized clinical trials and integrated it into the evidence synthesis.

Statistical analysis and data synthesis

We provided a narrative synthesis of the findings of the included reviews and if multiple reviews existed for the same intervention. However, in the case of ARBs, we updated the evidence and meta-analyzed the data using random-effects and fixed-effects model with Mantel-Haenszel methods [13] and reported random-effects model to account for heterogeneity. Meanwhile, we evaluated each intervention against our outcomes of interest and synthesized the evidence taking into account heterogeneity and inconsistencies between reviews. As a rule of thumb, I^2 (I-square) values of 25%, 50% and 75% correspond to low, moderate and high levels of heterogeneity, respectively [14].

For peer review only - http://bmjopen.bmj.com/site/about/guidelines.xhtml

For the purpose of our overview, we categorized the evidence of the included interventions into three categories: 1) effective interventions; 2) ineffective interventions; 3) uncertain evidence (conflicting or inconclusive evidence). We used relative effect [odds ratio (OR) or risk ratio (RR)] as a summary statistic from the most recent or largest published systematic reviews, and confidence intervals (CIs) of 95% with a significance level determined at two-sided alpha less than 5%.

Patient and Public Involvement

Our study did not involve direct contact with patients or the public

For peer review

Results

Search result

According to our pre-defined eligibility criteria, our search strategy in electronic databases and manual searches resulted in 41 studies [6 15-54]. Fig 1 shows the search strategy results. At full-text level, we excluded studies that did not assess our outcome of interest (n=129), were narrative reviews (n=4), did not include HF patients (n=8), included preserved heart failure patients (n= 3), were duplicate or had no full text (n=2).

Characteristics of the included reviews

As shown in **Table 1**, the population of the included reviews consisted of HF patients with an ejection fraction ≤ 45% in most studies and a corresponding New York Heart Association (NYHA) classification ranging from I-IV. The effectiveness of each drug intervention has been assessed in at least one review. All reviews were systematic, except two reviews for anti-arrhythmic drugs. At the time of their publication, 15 out of 41 reviews (37%) had corresponding authors based in the USA, 7 (17%) in Canada, 6 (15%) in China, 3 in Chile, 2 in France, 2 in the UK, and the 6 remaining in other countries.

The disclosure and reporting of financial resources or funding varied from one study to another. Twenty-one reviews (51%) did not report the source of funding. Ten reviews (24 %) reported financial supports that included governments, academic institutions, and device industry. Six reviews declared financial resources as none or no external funds. Three reviews reported industry sponsorship for at

For peer review only - http://bmjopen.bmj.com/site/about/guidelines.xhtml

least one author. One review [53] stated that one author obtained funds for the review without clarifying the source (**S4 table**). We also reported findings summary of each review as stated by their respective authors (**Table 1**).

For peer review only

Table 1: Summary of characteristics of included studies of heart failure (ordered by intervention)

Author (year); country; reference	Review type	Intervention/ Comparator	Population type; Ejection fraction (%); NYHA	Study design n; participants n	Mean follow-up/Range (months)	Authors' Findings summary	AMSTAR score
Al-Gobari M., et al. (2013), France [16≠]	Systematic review & meta-analysis	Beta-blockers /Placebo; "usual care"	HF; <45% except one study < 62%; I-IV	RCTs n= 30; n = 24,779	Mean: 11.51	Beta-blockers significantly reduced SCD, cardiovascular death and all-cause mortality	6
Chatterjee S., et al. (2013); USA [15]	Systematic review & meta-analysis	Beta-blockers / Placebo; beta-blocker; "usual care"	HF; <45%; II-IV	RCTs n=21 ; n= 23,122	median: 12	The study confirmed mortality benefits of BBs compared to placebo or usual care in heart failure with reduced ejection fraction.	8
Brophy JM. Et al., (2001); Canada [17]	Meta-analysis	Beta-blockers / Placebo; "usual care"	CHF; <45%; I-IV	RCTs n= 22 ; n= 10,135	Range: 3 to 23	This study reported a reduction in mortality and morbidity in CHF.	4
Lee S. et al., (2001), USA [18]	systematic review & meta-analysis	Beta-blockers / Placebo	HF; <30%; II-III	RCTs n=6 ; n= 9335	Range : 12 to 23	The authors recommended use of beta-blockers in HF with reduced ejection fraction and NYHA II-III.	4
Bonet S., et al. (2000), USA [19]	Meta-analysis	Beta-blockers / Placebo ; "usual care"	HF; <45%; NA	RCTs n= 21 ; n= 5,849	Median: 6	Beta-blockers reduce total mortality by reducing pump failure and sudden cardiac death events. Vasodilating beta-blockers have perhaps greater effects on overall mortality than nonvasodilating agents.	4
Heidenreich P.A., et al. (1997); USA [20]	Meta-analysis	Beta-blockers / Placebo; "usual care"	HF; <30%; I-IV	RCTs n= 17 ; n= 3,039	Range: 3 to 24	Beta-blockers significantly reduced all-cause mortality but showed a trend toward better reduction in nonsudden cardiac death compared to sudden cardiac death.	5
Le HH. et al., (2016); France [21≠]	Systematic review & meta-analysis	Anti-aldosterone / Placebo; "usual care"	HF, post-MI; <40%- >50%; I-IV	RCTs n=25; n = 19,333	Range: 3 to 39.6	In HF, anti-aldosterones or mineralocorticoid receptor blockers reduced SCD (subgroup analysis: 5 RCTs), all-cause mortality (subgroup analysis: 10 RCTs) and cardiovascular, all-cause and cardiovascular hospitalization. Adverse effects (hyperkalemia, degradation of renal function, gynecomastia) were, however, significantly higher in the treated group compared to placebo.	7
Bapoje SR. et al. (2013); USA [54]	Systematic review & meta-analysis	Anti-aldosterone / Placebo; "usual care"	HF; <45%; I-IV	RCTs n= 8; n= 11,875	Range: 3 to 24	Mineralocorticoid receptor antagonists (or aldosterone antagonists) reduced the risk of SCD in patients with left ventricular dysfunction.	8
Wei J., et al. (2010); China [22]	Meta-analysis	Anti-aldosterone / Placebo; "usual care"	HF; <45%; NA1	RCTs n= 6 (two are not double blind), n= 00000	Range: 2 to 24	Two [Pitt et al, 1995 and Pitt et al 2003] of the six-included studies showed a significant reduction of SCD in the group of Spironolactone vs placebo and the group of Eplerenone vs. Placebo, cited respectively.	5
Solomon SD., et al. (2016); USA [23≠]	Meta-analysis	Sacubitril; valsartan / ACE-i	HF; <30%; II-IV	RCTs n= 3; n= 14742	Range 6 to 27	The authors concluded that combined neprilysin/RAS inhibition reduced all-cause mortality in HFrEF.	7
Flather MD et al., (2000); Canada [25]	Systematic review	ACE-i / Placebo	CHF; post-MI <45%; NA	RCTs n= 5, n= 12,763	Range: 15 to 42	This meta-analysis showed a lower risk of death in ACE-i treated group compared to placebo.	NA
Garg R. et al., (1995); Canada [24≠]	Systematic review & meta-analysis	ACE-i / Placebo	CHF; <45% ; I-IV	RCTs n= 32, n= 7,105	Range: 3 to 42	Overall, this study reported a significant reduction of total mortality (attributed mainly to less progressive HF deaths) and hospitalization for worsening HF.	2

For peer review only - http://bmjopen.bmj.com/site/about/guidelines.xhtml

Rain & Rada, (2015) ; Chile [26]	Systematic review	ARB/ ACE-i	HF ; <45% - < 35% ; II-IV	RCTS = 8 ; n= 5201	NA	The authors concluded that ARBs are probably as effective in mortality reduction as ACE-i with probably less withdrawal rate due to adverse effects.	NA
Heran et al., (2012) ; Canada [27*]	Systematic review & meta-analysis (Cochrane)	ARB (or ARB + ACE-i) / Placebo ; ACE-i	HF ; ≤40% ; II-IV	RCTS n= 24 ; n= 25,051	Range: 1 to 49.5	Compared to placebo or in addition to ACE-i, ARBs did not reduce all-cause mortality.	10
Shibata et al., (2008) ; Canada [28]	Systematic review & meta-analysis	ARB/ Placebo ; ACE-i	HF ; <40% ; I-IV	RCTs n= 7 ; n= 27,495	Range: 11 to 41	Compared to ACE-i or used in combination, ARBs provided no beneficial effects on mortality. A 17% reduction in hospitalizations was observed.	4
Lee et al., (2004) ; USA [29]	Meta-analysis	ARB/ Placebo ; ACE-i	CHF , AMI ; ≤45% ; II–IV	RCTs n= 24 ; n= 38,080	Range: 1 to 41	Compared to ACE-i, ARBs do not differ in efficacy for reducing all-cause mortality in CHF and AMI patients.	7
Dimopoulos et al., (2004) ; UK [30]	Meta-analysis	ARB/ Placebo ; ACE-i	CHF; <40% ; II–IV	RCTs n= 4; n = 7666	Mean: 31	ARBs can be used to prevent events in ACE-i-treated heart failure patients who are not suitable for beta-blockers.	3
Jong P., et al. (2002), Canada [31]	Systematic review & meta-analysis	ARB (or ARB + ACE-i) / Placebo ; ACE-i	HF; ≤35% - ≤45%; II–IV	RCTs n= 17; n = 12,469	Range: 1 to 23	The authors could not conclude any superiority of ARBs versus controls, stating this might be due to the use of ACE-i as a comparator or background treatment in the majority of included trials.	8
Rain & Rada, (2017); Chile [32]	systematic review	Statins / Placebo ; "usual care"	HF; <45%; I-IV	RCTs n= 25; n = NR	NA	The authors summarized that statins do not decrease mortality in chronic heart failure and might lead to a small reduction in hospital admissions for HF.	NA
Al-Gobari M., et al. (2017), Switzerland [6≠]	Systematic review & meta-analysis	Statins / Placebo ; "usual care"	HF, ischaemic/nonischaemic; NA; I-IV NA	RCTs n= 24; n = 11,463	Range: 1 to 46.8	Statins do not significantly reduced SCD and all-cause mortality. They may or may not reduce hospitalizations due to worsening heart failure (HWHF)	7
Bonsu KO, et al. (2015), Malaysia [33]	Meta-analysis	Statins / Placebo ; "usual care"	HF; <45%; I-IV	RCTs n= 13; n = 10,966	Range: 3 to 46.8	Lipophilic statins showed significant decrease in all-cause mortality, cardiovascular mortality and hospitalization for worsening heart failure.	8
Wang JQ., et al. (2014); China [34]	Meta-analysis	Statins / Placebo ; "usual care"	HF; NA; NA	RCTs n= 6 (9: observational studies), n= 10,016	Range: 12 to 46.8	The authors concluded that statins reduce sudden cardiac death and all-cause mortality in HF.	5
Liu G. et al. (2014); China [35]	Meta-analysis	Statins / Placebo ; "usual care"	HF; <45%; I-IV	RCTs n=13 ; n = 1,532	Range: 3 to 35.5	The authors reported significant decrease in all-cause mortality but recommended cautious interpretation and further research.	7
Rahimi K. et al. (2012); UK [40]	Meta-analysis	Statins / Placebo ; "usual care"	HF, MI, Primary prevention, diabetes, ACS, CHD; NA; NA	RCTs n= 37 n= 155,020		Statins have a modest effect on SCD but no substantial protective effect on ventricular arrhythmic events.	6
Zhang S. et al. (2011); China [36]	Meta-analysis	Statins / Placebo ; "usual care"	HF; <45%; I-IV	RCTs n=13 ; n = 10,447	Range: 2 to 46.8	This meta-analysis concluded of no difference between treatment groups but benefits may occur in some specific populations and with a specific statin.	7
Xu M. et al. (2010); China [37]	Meta-analysis	Statins / Placebo ; "usual care"	HF; <45%; I-IV	RCTs n= 7, n= 540	Range: 3 to 31	The authors suggested that atorvastatin treatment is effective and reduce all-cause mortality and hospitalization for worsening HF.	6
Lipinski MJ. (2009); USA [38]	Meta-analysis	Statins / Placebo ; "usual care"	HF; <45%; I-IV	RCTS n= 10; n= 10,192	Range 3 to 47	The authors stated that statins are safe and improve LVEF and decrease hospitalization for worsening HF.	7
Levantesi G. et al. (2007); Italy [39]	Meta-analysis	Statins / Placebo ; "usual care"	Secondary prevention; NA; NA	RCTs n= 10 n= 22,275	Range: 6 to 73.2	Statins were associated with a significant risk reduction for SCD (in secondary prevention settings).	3
Claro JC., et al. (2015), Chile [41≠]	Systematic review & meta-	Amiodarone / Placebo; "usual care"	Sub-analysis : heart failure ; NA; NA	RCTs n= 11, n= 5,006	NA	In HF sub-population, amiodarone showed a statistically significant reduction for SCD but not for all-cause mortality. Authors judged the quality of the evidence for the whole	10

For peer review only - http://bmjopen.bmj.com/site/about/guidelines.xhtml

	analysis (Cochrane)					population (primary prevention) as low to moderate and for secondary prevention population as very low.	
Santangeli P. et al. (2012); USA [42]	systematic review	Amiodarone / Placebo	cardiovascular disease; NA; NA	NA	NA	Amiodarone has less favorable net clinical benefits for prophylaxis of SCD because of adverse effects.	5
Piccini JP., et al. (2009); USA [43]	Meta-analysis	Amiodarone / Placebo ; "usual care"	HF, AMI; <45%; II-IV	RCTs n= 15, n= 8,522	Range: 2 to 12	In HF sub-population, amiodarone showed a statistically significant reduction for SCD but not all-cause mortality.	7
ATMA Investigators (1997) [44]	Meta-analysis	Amiodarone / Placebo ; "usual care"	Post-MI and CHF; 31%; NA	NA	Range: 4.8 to 25.8	Amiodarone reduced arrhythmic/sudden death in high-risk patients with recent MI or CHF. All-cause mortality decreased by 13 %.	NA
Sim I. et al., (1997); USA [45]	Meta-analysis	Amiodarone / Placebo ; "usual care"	Subgroup : heart failure; <45%; NA	RCTs n=5, n= 4,125	Range: 6 to 45.6	Amiodarone reduced all-cause mortality in high SCD risk groups.	5
Das MK. et al., (2010): USA [46]	Narrative Review	Anti-arrhythmics / Placebo ; "usual care"	Subgroup : heart failure; NA; NA	NA	NA	Class I Anti-arrhythmic drugs (AADs) increased all-cause mortality and SCD in Post-MI patients. Amiodarone (class III AADs) decreased or have neutral effect on SCD. Caution is warranted to outweigh risks of proarrhythmia and other adverse effects.	NA
Hilleman DE. et al., (2001); USA [47]	Narrative Review	Anti-arrhythmics / Placebo ; "usual care"	HF; <45%; NA	RCTs n= 6 n= 10440	Range: 6.5 to 45	Beta-blockers (Bisoprolol, Carvedilol, Metoprolol) reduced total mortality and SCD in HF. Class I antiarryhthmics increased mortality and SCD in a post hoc analysis of SPAF-I study. Amiodarone had mixed results and dofetilide did not reduce mortality or SCD.	NA
Rizos EC., et al. (2012); Greece [48]	systematic review & meta-analysis	Omega 3 Fatty acids / Placebo ; "usual care"	cardiovascular diseases; NA; NA	RCTs n= 20; n= 68,680	NA	Omega-3 PUFAs supplementation were not associated with a lower risk of all-cause mortality or sudden cardiac death	8
Kotwal S. et al. (2012); Australia [49]	systematic review & meta-analysis	Omega 3 Fatty acids / Placebo ; "usual care"	cardiovascular diseases, heart failure admissions; NA; NA	RCTs n= 20; n= 62,851	Range: 6 to 72	The authors concluded that there is no clear effect on total mortality and sudden death outcomes.	7
Kwak SM., et al. (2012); Korea [50]	Meta-analysis	Omega 3 Fatty acids / Placebo ; "usual care"	secondary prevention of cardiovascular disease; NA; NA	RCTs n=14; n= 20,485	NA	This meta-analysis concluded of insufficient evidence.	8
Chen Q., et al. (2011); China [51]	Meta-analysis	Omega 3 Fatty acids / Placebo ; "usual care"	cardiovascular disease; NA; NA	RCTs n= 10; n= 33,429	NA	Omega-3 fatty acids did not appear to reduce SCD under guideline-adjusted treatment for CVD secondary prevention.	7
Marik PE., et al. (2009); USA [52]	systematic review	Omega 3 dietary supplements / Placebo; olive oil ; corn oil, sunflower oil ; "usual care"	cardiovascular disease; NA; NA	RCTs n= 11; n= 39,044	Range: 12 to 55.2	Dietary supplementation with omega-3 fatty acids reduced SCD and all-cause mortality	4
Wang C., et al. (2006); USA [53]	systematic review	n-3 Fatty acids / Placebo/olive oil ; corn oil, sunflower oil ; "usual care"	Primary and secondary prevention; NA; NA	RCTs n= 12; n= 32,981	Range: 12 to 48	The authors concluded of a significant reduction in all-cause mortality and SCD with n-3 Fatty acids from fish or fish-oil supplements but not α-linolenic acid.	6

≠: studies considered in Table 2 as "reference" studies. *: updated in this study. NA: not applicable

For peer review only

Risk of bias and quality of reviews

As shown in **Table 1**, the AMSTAR scores for quality assessment of the included reviews widely ranged from 2 to 10 (out of 11). All reviews had one score less because of non-listing of excluded primary studies except Cochrane reviews [27 41] which scored 10 because of non-inclusion of grey literature in the search strategy in one review and missing information for funding resources of included primary studies in another, cited respectively (**S3 Table**). We did not assess the AMSTAR score for six studies, of which two [46 47] were narrative reviews, two[25 44] were individual participant or patient data (IPD) meta-analyses, and the other two [26 32] were overviews of reviews.

The risk of bias of the included primary studies within reviews remained as judged by the original reviews' authors with the exception of the newly added randomized trials in the update of the angiotensin receptor blockers review that we assessed (GRADE) (**Table 2**). The quality of evidence for beta-blockers and anti-aldosterone agents obtained a high quality on the GRADE scale, while angiotensin-converting enzyme inhibitors (ACE-i), amiodarone and statins obtained a moderate quality. However, combined ARB/neprilysin inhibitors had a moderate and high quality for SCD and all-cause mortality outcomes respectively, whereas angiotensin receptor blockers had a low quality of evidence (**Table 2**).

Table 2: Summary of findings and Grade evaluation for sudden cardiac death (SCD) and all-cause mortality prevention

Drug interventions for SCD and all-cause mortality prevention in heart failure patients							
Outcome	Intervention / comparison	Assumed risk with comparator	Corresponding risk with intervention	Relative effect (95% CI)	Number of participants (No of studies)	Quality of the evidence (GRADE)	Comments
**Sudden cardiac death**							
	Beta-blockers / placebo	77 per 1000	54 per 1000 (49 to 60)	OR 0.69 (0.62 to 0.77)	24,779 (26 RCTs)	⊕⊕⊕⊕ high*	I^2=0% (P=0.57)
	Anti-aldosterone inhibitor / Placebo ;	61 per 1000	49 per 1000 (41 to 60)	RR 0.81 (0.67 to 0.98)	8,301 (5 RCTs)	⊕⊕⊕⊕ high*	I^2=8% (P=0.36)

	"usual care"						
	ARB;Neprilysin inhibitor/ ACE-i	74 per 1000	60 per 1000 (51 to 70)	RR 0.81 (0.69 to 0.95)	8,399 (1 RCsT)	⊕⊕⊕⊖ moderate[e]	
	ACE-i / Placebo	59 per 1000	54 per 1000 (43 to 65)	OR 0.91 (0.73 to 1.11)	6,988 (30 RCTs)	⊕⊕⊕⊖ moderate[b]	I^2=0% (P=0.94)
	ARB (or ARB + ACE-i) / Placebo ; ACE-i	See comment	See comment	Not estimable	13,884 (5 RCTs)	⊕⊕⊖⊖ Low[b,c]	I^2=78% (P=0.010). Overall, we did not pool the studies because of heterogeneity
	Statins / placebo ; "usual care"	108 per 1000	100 per 1000 (76 to 131) [99 per 1000 (72 to 131)]	RR 0.92 (0.7 to 1.21) [OR 0.90 (0.64 to 1.24)]	10,077 (8 RCTs)	⊕⊕⊕⊖ moderate[a]	I^2=42.6% (P=0.094
	Amiodarone / Placebo; "usual care"	118 per 1000	93 per 1000 (79 to 110)	RR 0.79 (0.67 to 0.93)	5,006 (11 RCTs)	⊕⊕⊖⊖ Low[a,b]	
	Omega 3 Fatty acids / Placebo ; "usual care"	93 per 1000	88 per 1000 (77 to 102)	RR 0.94 (0.82 to 1.09)	6,975 (1 RCT)	⊕⊕⊕⊖ moderate[e]	
**All-cause mortality**							
	Beta-blockers / placebo	178 per 1000	127 per 1000 (113 to 141)	OR 0.67 (0.59 to 0.76)	24,779 (26 RCTs)	⊕⊕⊕⊕ high*	I^2= 40% (P=0.02)
	Anti-aldosterone inhibitor / Placebo ; "usual care"	200 per 1000	162 per 1000 (148 to 176)	RR 0.81 (0.74 to 0.88)	9,019 (10 RCTs)	⊕⊕⊕⊕ high*	I^2=0% (P=0.56)
	ARB;Neprilysin inhibitor/ ACE-i	183 per 1000	158 per 1000 (145 to 172)	RR 0.86 (0.79 to 0.94)	14,742 (3 RCTs)	⊕⊕⊕⊕ high	I^2=0% (P=0.42)

For peer review only

For peer review only - http://bmjopen.bmj.com/site/about/guidelines.xhtml

	ACE-i / Placebo	219 per 1000	178 per 1000 (158 to 198)	OR 0.77 (0.67 to 0.88)	7,105 (32 RCTs)	⊕⊕⊕⊖ moderate[b]	I^2=0% (P=0.95)
	ARB (or ARB + ACE-i) / Placebo ; ACE-i	183 per 1000	177 per 1000 (161 to 197)	RR 0.97 (0.88 to 1.08)	19,510 (27 RCTs)	⊕⊕⊖⊖ Low[b,d]	I^2=24% (P=0.14)
	Statins / placebo ; "usual care"	273 per 1000	240 per 1000 (205 to 278) [233 per 1000 (199 to 273)]	RR 0.88 (0.75 to 1.02) OR 0.81 (0.66 to 1)	11,024 (13 RCTs)	⊕⊕⊕⊖ moderate[a]	I^2=37.7% (P=0.083)
	Amiodarone / Placebo ; "usual care"	264 per 1000	237 per 1000 (211 to 266)	RR 0.90 (0.80 to 1.01)	5,006 (11 RCTs)	⊕⊕⊖⊖ Low[a,b]	
	Omega 3 Fatty acids / Placebo ; "usual care"	291 per 1000	274 per 1000 (253 to 294)	RR 0.94 (0.87 to 1.01)	6,975 (1 RCT)	⊕⊕⊕⊖ moderate[e]	

[a] Likelihood of Publication bias presence with an asymmetric funnel plot.

[b] The studies reported to generally have a moderate to high risk of bias due to allocation concealment and blinding reporting.

[c] Inconsistent results ranged from no effect to insignificant increase of events (I^2 = 71%)

[d] Most studies have small sample and wide confidence intervals including no effect with appreciable harm or benefit.

[e] Estimation comes from one single clinical trial. No data obtained from other relevant studies for this outcome

* Although graded high, this might be downgraded into moderate if we strictly consider the risk of bias of primary studies other than an overall estimation.

RR: risk ratio; OR: odds ratio; CI: confidence interval; GRADE: Grading of Recommendations, Assessment, Development and Evaluation; I^2: between-study variance due to heterogeneity.

For peer review only - http://bmjopen.bmj.com/site/about/guidelines.xhtml

For peer review only

For peer review only

Up-to-dateness of included reviews

Most retrieved evidence was published within the last 10 years (2008 and on) and seven (out of nine) drug interventions with updated systematic reviews were within the last five years (2012 and on). Moreover, we updated the pooled results for ARBs, which resulted in slightly different results compared to the original Cochrane review [27].

Effectiveness of interventions

We report below the summaries of our evaluation on the effectiveness of the drug interventions considered that we have categorized into effective, ineffective, and uncertain effectiveness (inconclusive or conflicting evidence).

Effective interventions

Beta-blockers (BBs)

Meta-analyses and systematic reviews of randomized controlled trials [15-20] provided overwhelming evidence that beta-blockers (BBs) decrease the risk of SCD and all-cause mortality in HFrEF patients. The quality of the evidence was rated high with a relative effect of 0.69 for SCD [OR, 95% CI (0.62 to 0.77)], and of 0.67 for all-cause mortality [OR, 95% CI (0.59 to 0.76)] (**Table 2**).

Anti-aldosterone agents

Published studies about mineralocorticoid-receptor antagonists (antimineralocorticoids) or (so-called) anti-aldosterones appeared effective in SCD and all-cause mortality prevention [21 22 54]. However, in a recent systematic review [21], adverse effects (hyperkalemia, degradation of renal function, gynecomastia) were significantly higher in the anti-aldosterone-treated group compared to placebo. The quality of the evidence was rated high with relative effect for SCD [RR 0.81, 95% CI (0.67 to 0.98)], and all-cause mortality [RR 0.81, 95% CI (0.74 to 0.88)] (**Table 2**).

Angiotensin receptor blocker (ARB)/neprilysin inhibitor

One meta-analysis [23] estimated the effects of combined neprilysin renin-aldosterone system inhibition and reported a reduction in SCD and all-cause mortality. The finding was principally derived from one RCT (PARADIGM-HF) [5] that showed about 16% reduction of all-cause mortality in favor of sacubitril/valsartan (LCZ696 previously) compared to enalapril (an ACE-i). This mortality reduction was attributed to a decline on both SCD (20%) and pump failure deaths [55]. **Table 2** shows the relative effect for SCD [risk ratio (RR) 0.81, 95% confidence interval (CI) (0.69 to 0.95)], and all-cause mortality [RR 0.86, 95% CI (0.79 to 0.94)]. The moderate quality of the evidence for SCD outcome

For peer review only - http://bmjopen.bmj.com/site/about/guidelines.xhtml

was due to estimation from one single clinical trial and absence of data from other included studies. All-cause mortality was, however, rated as high with a possibility of downgrading in case of a detectable publication bias or unestablished class effect.

Ineffective Interventions

Angiotensin-converting enzyme inhibitors (ACE-i)

Although two systematic reviews [24 25], with an AMSTAR score of 2/11 and 3/11 respectively, reported a decline in total mortality and less progressive HF deaths, SCD events did not significantly decrease [OR 0.91, 95% CI (0.73 to 1.12)]. The quality of the evidence was rated as moderate because of the unclear or high risk of bias in included primary studies (**Table 2**).

Angiotensin receptor blockers (ARBs)

As shown in **Fig 2** and **Fig 3**, we updated a Cochrane review [27] by including more eligible primary studies such as SUPPORT trial.[56] Comparing ARBs to controls resulted in slightly different effect size estimation. Eventually, we did not combine the different control groups to account for heterogeneity. In stratified analyses, ARBs compared to placebo remained ineffective for all-cause mortality [RR 0.79, 95% CI (0.55 to 1.13)]. Similarly, ARBs, compared to ACE-I or in combination versus ACE-i alone, were not superior in all-cause mortality reduction [RR 0.87, 95% CI (0.56 to 1.36); RR 0.99, 95% CI (0.90 to 1.09), respectively] (**Fig 2**). The quality of the evidence is rated as low because of risk of bias, imprecision and inconsistency ($I^2 \approx 74$, P=0.010 for SCD outcome) (**Table 2**). Data were limited for studies reporting SCD, in particular those comparing ARBs versus placebo, or versus ACE-i (**Fig 2**). In addition, the funnel plot for all-cause mortality outcome showed no evidence of publication bias (**Fig 4**). No estimation of publication bias and no funnel plot was drawn for SCD as only five studies reported this outcome.

Statins

Published systematic reviews and meta-analyses about statins in HF were inconsistent [6 32-40] with a recent tendency toward inefficacy in total mortality and SCD prevention. The quality of the evidence was rated as moderate because of a likelihood of publication bias revealed on the most up-to-date systematic review [6] (**Table 2**).

<u>Unclear evidence of effectiveness</u>

The evidence of effectiveness of the drug interventions reported below was considered uncertain due either to conflicting or inconclusive evidence.

For peer review only - http://bmjopen.bmj.com/site/about/guidelines.xhtml

Amiodarone & anti-arrhythmic drugs (AADs)

Recently published systematic reviews[41 43] for amiodarone showed a significant reduction for SCD but not for all-cause mortality with less favorable net clinical benefits[42]. Other older reviews [44 45] of minor quality (AMSTAR of 3/11 and 5/11 cited respectively) reported a decline of both SCD and all-cause mortality. The quality of evidence for amiodarone was rated as low because of the unclear or high risk of bias and potential publication bias in primary studies (**Table 2**). No systematic review for AADs of other classes or drugs (other than amiodarone) were retrieved. Nevertheless, two narrative reviews [46 47] reported that class I anti-arrhythmics increased SCD and all-cause mortality. These narrative reviews called for caution regarding the mixed results of amiodarone and its adverse effects.

Omega-3 polyunsaturated fatty acids (PUFA) and fish oil supplementation

No systematic review was exclusively conducted in heart failure patients for this intervention. One primary study[57], known as GISSI-Prevenzione HF, recruited patients with chronic heart failure and reported a lower mortality events in the n-3 polyunsaturated fatty acids (PUFA) group compared to the placebo group. The authors reported an adjusted hazard ratio of 0.91 [95.5% CI 0.833 to 0.998], p=0.041). However, relative risk in our analysis remained statistically insignificant [RR 0.94, 95% CI (0.87 to 1.01), p=0.10] and [RR 0.94, 95% CI (0.81 to 1.09), p=0.42] for all-cause mortality and SCD, respectively. Our assessment of the quality of the evidence involving GISSI-Prevenzione HF was moderate because of an absence of data of any other relevant studies (**Table 2**). In addition, some recent systematic reviews [48-51] included patients regardless of their cardiovascular disease and concluded of no clear effect, insufficient evidence or no reduction on SCD and all-cause mortality outcomes. Meanwhile, some older studies [52 53] reported that omega-3 fatty acids and fish-oil supplements (other than α-linolenic acid [53]) reduced SCD and all-cause mortality.

Discussion

Our assessment of the effectiveness of drug interventions to prevent SCD in HFrEF patients indicated that beta-blockers (BBs), antialdosterone agents, as well as combined angiotensin receptor blocker (ARB)/neprilysin inhibitors were effective.

Previously reported meta-analyses and systematic reviews of RCTs [15-20] indicated that beta-blockers are effective in the prevention of SCD and all-cause mortality in HFrEF. However, although they were increasingly used as a usual “routine” care in the compared arms of the more recently published clinical trials[58], BBs stayed underused for long time and may still be [59]. Mineralocorticoid receptor antagonists or antialdosterone drugs have been reported effective in HFrEF by reducing SCD and all-

For peer review only - http://bmjopen.bmj.com/site/about/guidelines.xhtml

cause mortality [21 22 54 60]. Our summary of the findings and the consistency of the results support this claim with a high quality of evidence. Only one retrieved meta-analysis [23] supported the effectiveness of combined ARB/neprilysin inhibitor. The authors acknowledged the limitation of their meta-analysis, which was not based on a systematic review, but merely pooling three well-known trials published in high impact journals (i.e., IMPRESS [61], OVERTURE [62] and Paradigm-HF [5]). The quality of the evidence is, however, moderate for SCD and high for all-cause mortality, albeit our inability to assess any presence of a class effect or a potential publication bias.

We found that ACE-i showed a total mortality reduction in clinical trials and systematic reviews of heart failure patients [24 25]. However, our overview showed that ACE-i, surprisingly, did not significantly decrease SCD with a moderate quality of evidence.

In addition, we found that neither ARBs nor statins reduced SCD and/or all-cause mortality. Our findings for ARBs were in agreement with Jong and colleagues [31], Shibata et al. [28] and Dimopoulos et al. [30], but in contradiction to Lee VC et al. [29] and Rain & Rada's conclusions [26]. Our up-to-date meta-analysis for ARBs included only five primary studies, but large-scale trials, that reported SCD events. Eventually, we did not pool all the different comparators together but separately estimated the effect size for each group to account for the heterogeneity. Moreover, the addition of current trials such as SUPPORT [56], improved the statistical power of detecting an effect if existed and the summary statistic remained statistically insignificant (**Fig 2**). Of note, Jong and colleagues [31] attributed this inefficacy of ARBs in HF to the background treatment with ACE-i.

Within the current evidence, ARBs should not be seen as interchangeable with ACE-i, which also showed a neutral effect on SCD, without a proper reason. Therefore, in a high-risk SCD patient, another therapeutic strategy should be sought and an ARB/neprilysin inhibitor might be an alternative in patients similar to those of the Paradigm-HF trial[5].

The addition of statins to the therapy regimen of heart failure patients had no survival benefits. Actually, a recent systematic review and meta-analysis indicated that statins did not reduce SCD nor all-cause mortality [6]. Our current study reached the same conclusion with similar quality of evidence.

Our overview showed unclear evidence of effectiveness of Omega-3 polyunsaturated fatty acids (PUFA), fish oil supplementation and AADs. The latter intervention had an evidence originated from only narrative reviews, as we did not identify any systematic reviews. Also, only one n-3 PUFA clinical trial [57] was conducted in HF patients and reported a statistically significant mortality reduction; this result was not supported by other trials and recent systematic reviews [48-51], a finding that justified our

For peer review only - http://bmjopen.bmj.com/site/about/guidelines.xhtml

conclusion of unclear evidence. Moreover, no other data or systematic reviews conducted in HF were retrieved by our electronic and manual searches.

AADs are classified into four categories [46]: sodium channel blocking drugs (class I), BBs (class II), potassium channel blockers (class III), and calcium channel blockers (class IV). We found inconclusive evidence of effectiveness of all categories, with the exception of BBs. The evidence of effectiveness of class I, III and IV is inconclusive, neutral or even detrimental to patients as for class I AADs [46 47]. Amiodarones, which present class I, II, III, and IV effects, reported mixed results with potential SCD prevention with adverse effects [43] and potentially, but rare [63], life-threatening proarrhythmias [46].

Our overview has some limitations. First, we limited the scope of our study to drug treatment, thus excluding devices like implantable cardioverter defibrillators. We believe that non-drug devices should be tackled in future research. ESC Guideline (2016)[64] and others (e.g. www.uptodate.com) recommend the use of ICDs for only ≤ 35% HF patients and only after optimization of drug therapy. In fact, SCDs occur in both reduced and preserved heart failure. Our overview might help to optimize therapy as a first step before introducing ICDs, which applies to a limited HF subpopulation, regardless of costs. Second, we may have failed to include other drug interventions used in HFrEF. Such drug candidates include digoxin, I_f-channel blockers (ivabradine), hydralazine/isosorbide dinitrate, nitroglycerin, and phosphodiesterase 3 or 5 inhibitors. However, our overview included most commonly prescribed and evidence-based pharmacological therapy in HF as pre-specified in our published protocol[11]. Third, we did not use specific drug names in our literature search strategy, in order to avoid omitting a therapy that evaluated SCD and/or all-cause mortality prevention in heart failure patients. Fourth, we based our analyses on existing systematic reviews and meta-analyses and we updated only one meta-analysis. Consequently, we were unable to update the evidence for ACE-inhibitors. Furthermore, as indicated by the AMSTAR score, the methodological quality of some of the existing reviews was suboptimal. Fifth, we did not assess the safety of the evaluated drug interventions, nor the contra-indications for their prescription, drug-drug interactions, as well as treatment adherence. Indeed, we considered that these important aspects were out of the scope of our analysis. Sixth, we were unable to do a sensitivity analysis, initially suggested in our protocol, for ischaemic versus non-ischaemic heart failure due to limited data availability. Finally, a potential source of bias relates to authors of this overview being the authors of three of the included reviews [16 21 65]. However, the adopted methodology is in line with systematic reviews guidelines and ensured a

For peer review only - http://bmjopen.bmj.com/site/about/guidelines.xhtml

double check of data and methodological evaluation by at least two reviewers and a published protocol[11].

It is noteworthy that high quality evidence does not necessarily imply strong recommendations, and strong recommendations can arise from low quality evidence [66]. Therefore, when one intervention is graded high, it is not our intention to say that it is highly recommended, as we did not assess the level of recommendation in our study. In fact, level of recommendation not only depends on the strength of evidence, but also (among others) on values and preferences of patients, net benefits and cost-effectiveness of a particular intervention.

Implications for practice

Our study summarizes and synthesizes the effectiveness of most evidence-based drug interventions in HF patients with reduced ejection fraction (HFrEF) for SCD prevention. It classified drug interventions according to the current evidence of their effectiveness. This categorization could help health professionals and patients making evidence-based decisions based on updated knowledge, particularly whenever a high-risk SCD patient is identified. Currently, there is no an established strategy to deal with patients at high risk of SCD. In such patients, a particular attention should be considered and a careful selection of available therapeutic options is needed. Furthermore, there might be a shift towards an alternative therapeutic strategy based on SCD prevention-effective drugs in light of our findings.

Conclusion

Our overview indicates that only three drug interventions (BBs, anti-aldosterones, combined ARB/neprilysin inhibitors) significantly reduce SCD and improve overall survival among individuals with heart failure and reduced ejection fraction. On the other hand, there is no evidence of effectiveness of ARBs to reduce neither all-cause mortality nor SCD (with a low quality of evidence), and ACE-i do not significantly reduce SCD events. When the goal of drug therapy is to reduce SCD, especially in high-risk patients, our synthesis supports the use of the most effective regimen.

Author contribution

Design and conception: MA, FG, BB. (MA is the guarantor). Project administration: BB. Writing original draft: MA. Critical analysis: MA, SA, FG, BB. Data curation: MA, SA. Statistical analysis: MA. Proofread and approved the final draft: MA, SA, FG, BB.

For peer review only - http://bmjopen.bmj.com/site/about/guidelines.xhtml

For peer review only

Competing interests

We declare that some authors of this overview are also authors of some of the included reviews. However, at least two reviewers systematically checked and validated the extracted data including study qualities. We declare that no other competing interests exist.

Funding

MA, the corresponding author, received a financial grant (2014.0193) from The Federal Department of Economic Affairs, Education and Research (EAER), Switzerland, also from Département universitaire de médecine et santé communautaires (DUMSC), Lausanne university hospital (CHUV), Switzerland. The former funders had no role in study design or conception, study analysis, writing or preparation of the manuscript.

Ethics committee approval

This study did not need to ask for ethical approval and no direct patient involvement occurred.

Data sharing

All data are available in the manuscript and its supported files. Any more information can be requested from the corresponding author.

Acknowledgements

We thank Emilie Zuercher for her technical assistance.

Supporting materials

- S1 File: PRISMA checklist
- S2 File: Search strategy equations and result
- S3 Table: AMSTAR scores.
- S4 Table: Funding data of included reviews

Abbreviations

- ACE-i: angiotensin-converting enzyme inhibitor
- ACS: acute coronary syndrome
- ARBs: angiotensin receptor blockers

For peer review only - http://bmjopen.bmj.com/site/about/guidelines.xhtml

- ATMA: amiodarone trial meta-analysis
- BBs: beta-blockers
- CHD: coronary heart disease
- CHF: chronic heart disease
- GRADE: Grading of Recommendations, Assessment, Development and Evaluation
- HF: heart failure
- HFrEF: heart failure with reduced ejection fraction
- LVD: left ventricular dysfunction
- MI: Myocardial Infarction
- NA: not available or not applicable.
- NR: not reported
- NYHA: New York Heart Association class
- Post-MI: post myocardial infarction
- PUFAs: polyunsaturated fatty acids
- RAS: renin angiontensin system
- RCTs: randomized controlled trials
- SCD: sudden cardiac death
- SPAF-I: Stroke prevention Atrial Fibrillation Study
-
-

References

1. Thom T, Haase N, Rosamond W, et al. Heart disease and stroke statistics--2006 update: a report from the American Heart Association Statistics Committee and Stroke Statistics Subcommittee. *Circulation* 2006;113(6):e85-151. doi: 10.1161/circulationaha.105.171600 [published Online First: 2006/01/13]
2. Tomaselli GF, Zipes DP. What causes sudden death in heart failure? *Circulation research* 2004;95(8):754-63. doi: 10.1161/01.RES.0000145047.14691.db [published Online First: 2004/10/16]
3. Klein L, Hsia H. Sudden Cardiac Death in Heart Failure. *Cardiology Clinics* 2014;32(1):135-+. doi: 10.1016/j.ccl.2013.09.008
4. Ponikowski P, Voors AA, Anker SD, et al. 2016 ESC Guidelines for the diagnosis and treatment of acute and chronic heart failure: The Task Force for the diagnosis and treatment of acute and chronic heart failure of the European Society of Cardiology (ESC)Developed with the special contribution of the Heart Failure Association (HFA) of the ESC. *European heart journal* 2016;37(27):2129-200. doi: 10.1093/eurheartj/ehw128 [published Online First: 2016/05/22]
5. McMurray JJ, Packer M, Desai AS, et al. Angiotensin-neprilysin inhibition versus enalapril in heart failure. *The New England journal of medicine* 2014;371(11):993-1004. doi: 10.1056/NEJMoa1409077 [published Online First: 2014/09/02]

For peer review only - http://bmjopen.bmj.com/site/about/guidelines.xhtml

6. Al-Gobari M, Le HH, Fall M, et al. No benefits of statins for sudden cardiac death prevention in patients with heart failure and reduced ejection fraction: A meta-analysis of randomized controlled trials. *Plos One* 2017;12(2) doi: 10.1371/journal.pone.0171168
7. Lee E, Dobbins M, Decorby K, et al. An optimal search filter for retrieving systematic reviews and meta-analyses. *BMC medical research methodology* 2012;12:51. doi: 10.1186/1471-2288-12-51 [published Online First: 2012/04/20]
8. Wilczynski NL, Haynes RB. EMBASE search strategies achieved high sensitivity and specificity for retrieving methodologically sound systematic reviews. *Journal of clinical epidemiology* 2007;60(1):29-33. doi: 10.1016/j.jclinepi.2006.04.001 [published Online First: 2006/12/13]
9. Ouzzani M, Hammady H, Fedorowicz Z, et al. Rayyan-a web and mobile app for systematic reviews. *Systematic reviews* 2016;5(1):210. doi: 10.1186/s13643-016-0384-4 [published Online First: 2016/12/07]
10. Shea BJ, Grimshaw JM, Wells GA, et al. Development of AMSTAR: a measurement tool to assess the methodological quality of systematic reviews. *BMC medical research methodology* 2007;7:10. doi: 10.1186/1471-2288-7-10 [published Online First: 2007/02/17]
11. Al-Gobari M, Al-Aqeel S, Gueyffier F, et al. Effectiveness of drug interventions to prevent sudden cardiac death in patients with heart failure and reduced ejection fraction: an overview of systematic reviews. *PROSPERO 2017:CRD42017067442* 2017; Available from http://www.crd.york.ac.uk/PROSPERO/display_record.asp?ID=CRD42017067442
12. Kunz R, Burnand B, Schünemann HJ. Das GRADE-System. *Der Internist* 2008;49(6):673-80. doi: 10.1007/s00108-008-2141-9
13. Mantel N, Haenszel W. Statistical aspects of the analysis of data from retrospective studies of disease. *Journal of the National Cancer Institute* 1959;22(4):719-48. [published Online First: 1959/04/01]
14. Higgins JP, Thompson SG, Deeks JJ, et al. Measuring inconsistency in meta-analyses. *BMJ (Clinical research ed)* 2003;327(7414):557-60. doi: 10.1136/bmj.327.7414.557 [published Online First: 2003/09/06]
15. Chatterjee S, Biondi-Zoccai G, Abbate A, et al. Benefits of beta blockers in patients with heart failure and reduced ejection fraction: network meta-analysis. *BMJ (Clinical research ed)* 2013;346:no pagination.
16. Al-Gobari M, El Khatib C, Pillon F, et al. Beta-blockers for the prevention of sudden cardiac death in heart failure patients: a meta-analysis of randomized controlled trials. *Bmc Cardiovascular Disorders* 2013;13 doi: 10.1186/1471-2261-13-52
17. Brophy JM, Joseph L, Rouleau JL. Beta-blockers in congestive heart failure. A Bayesian meta-analysis. *Annals of internal medicine* 2001;134(7):550-60. [published Online First: 2001/04/03]
18. Lee S, Spencer A. Beta-blockers to reduce mortality in patients with systolic dysfunction: A meta-analysis. *Journal of Family Practice* 2001;50(6):499-504.
19. Bonet S, Agusti A, Arnau JM, et al. Beta-adrenergic blocking agents in heart failure: benefits of vasodilating and nonvasodilating agents according to patients' characteristics: a meta-analysis of clinical trials (Structured abstract). *Archives of Internal Medicine* 2000;160(5):621-27.
20. Heidenreich PA, Lee TT, Massie BM. Effect of beta-blockade on mortality in patients with heart failure: A meta-analysis of randomized clinical trials. *Journal of the American College of Cardiology* 1997;30(1):27-34.
21. Le HH, El-Khatib C, Mombled M, et al. Impact of Aldosterone Antagonists on Sudden Cardiac Death Prevention in Heart Failure and Post-Myocardial Infarction Patients: A Systematic Review and Meta-Analysis of Randomized Controlled Trials. *Plos One* 2016;11(2) doi: 10.1371/journal.pone.0145958
22. Wei J, Ni J, Huang D, et al. The effect of aldosterone antagonists for ventricular arrhythmia: a meta-analysis. *Clinical cardiology* 2010;33(9):572-7.
23. Solomon SD, Claggett B, McMurray JJ, et al. Combined neprilysin and renin-angiotensin system inhibition in heart failure with reduced ejection fraction: a meta-analysis. *European journal of heart failure* 2016;18(10):1238-43. doi: 10.1002/ejhf.603 [published Online First: 2016/07/02]
24. Garg R, Yusuf S. Overview of randomized trials of angiotensin-converting enzyme inhibitors on mortality and morbidity in patients with heart failure. Collaborative Group on ACE Inhibitor Trials. *Jama* 1995;273(18):1450-6. [published Online First: 1995/05/10]
25. Flather MD, Yusuf S, Kober L, et al. Long-term ACE-inhibitor therapy in patients with heart failure or left-ventricular dysfunction: a systematic overview of data from individual patients. ACE-Inhibitor Myocardial Infarction Collaborative Group. *Lancet* 2000;355(9215):1575-81. [published Online First: 2000/05/23]

For peer review only - http://bmjopen.bmj.com/site/about/guidelines.xhtml

26. Rain C, Rada G. Is there any difference between angiotensin converting enzyme inhibitors and angiotensin receptor blockers for heart failure? *Medwave* 2015;15 Suppl 1:e6177. doi: 10.5867/medwave.2015.06.6177 [published Online First: 2015/08/08]
27. Heran BS, Musini VM, Bassett K, et al. Angiotensin receptor blockers for heart failure. *The Cochrane database of systematic reviews* 2012(4):Cd003040. doi: 10.1002/14651858.CD003040.pub2 [published Online First: 2012/04/20]
28. Shibata MC, Tsuyuki RT, Wiebe N. The effects of angiotensin-receptor blockers on mortality and morbidity in heart failure: a systematic review. *International journal of clinical practice* 2008;62(9):1397-402. doi: 10.1111/j.1742-1241.2008.01806.x [published Online First: 2008/09/17]
29. Lee VC, Rhew DC, Dylan M, et al. Meta-analysis: angiotensin-receptor blockers in chronic heart failure and high-risk acute myocardial infarction. *Annals of internal medicine* 2004;141(9):693-704. [published Online First: 2004/11/03]
30. Dimopoulos K, Salukhe TV, Coats AJ, et al. Meta-analyses of mortality and morbidity effects of an angiotensin receptor blocker in patients with chronic heart failure already receiving an ACE inhibitor (alone or with a beta-blocker). *International journal of cardiology* 2004;93(2-3):105-11. doi: 10.1016/j.ijcard.2003.10.001 [published Online First: 2004/02/21]
31. Jong P, Demers C, McKelvie RS, et al. Angiotensin receptor blockers in heart failure: meta-analysis of randomized controlled trials. *J Am Coll Cardiol* 2002;39(3):463-70. [published Online First: 2002/02/02]
32. Rain C, Rada G. Are statins beneficial for chronic heart failure? First update. *Medwave* 2017;17(Suppl2):e6853. doi: 10.5867/medwave.2017.6853 [published Online First: 2017/04/30]
33. Osei Bonsu K, Reidpath DD, Kadirvelu A. Hydrophilic versus lipophilic statin therapy on outcomes in patients with heart failure: A systematic review and an adjusted indirect comparison meta-analysis of randomised trials. *European Heart Journal* 2015;36:546.
34. Wang JQ, Wu GR, Wang Z, et al. Long-term Clinical Outcomes of Statin Use for Chronic Heart Failure: A Meta-analysis of 15 Prospective Studies. *Heart Lung and Circulation* 2014;23(2):105-13.
35. Liu G, Zheng XX, Xu YL, et al. Effects of lipophilic statins for heart failure: a meta-analysis of 13 randomised controlled trials. *Heart, lung & circulation* 2014;23(10):970-7. doi: 10.1016/j.hlc.2014.05.005 [published Online First: 2014/06/24]
36. Zhang S, Zhang L, Sun A, et al. Efficacy of statin therapy in chronic systolic cardiac insufficiency: a meta-analysis. *European journal of internal medicine* 2011;22(5):478-84. doi: 10.1016/j.ejim.2011.06.003 [published Online First: 2011/09/20]
37. Xu M, Yuan G, Wei F. Effect of atorvastatin in patients with chronic heart failure - insights from randomized clinical trials. *Archives of medical science : AMS* 2010;6(6):866-73.
38. Lipinski MJ, Cauthen CA, Biondi-Zoccai GG, et al. Meta-analysis of randomized controlled trials of statins versus placebo in patients with heart failure. *The American journal of cardiology* 2009;104(12):1708-16. doi: 10.1016/j.amjcard.2009.07.055 [published Online First: 2009/12/08]
39. Levantesi G, Scarano M, Marfisi R, et al. Meta-analysis of effect of statin treatment on risk of sudden death. *The American journal of cardiology* 2007;100(11):1644-50. doi: 10.1016/j.amjcard.2007.07.015 [published Online First: 2007/11/27]
40. Rahimi K, Majoni W, Merhi A, et al. Effect of statins on ventricular tachyarrhythmia, cardiac arrest, and sudden cardiac death: A meta-analysis of published and unpublished evidence from randomized trials. *European Heart Journal* 2012;33(13):1571-81.
41. Claro JC, Candia R, Rada G, et al. Amiodarone versus other pharmacological interventions for prevention of sudden cardiac death. *Cochrane Database of Systematic Reviews* 2015(12) doi: 10.1002/14651858.CD008093.pub2
42. Santangeli P, Di Biase L, Burkhardt JD, et al. Examining the safety of amiodarone. *Expert Opinion on Drug Safety* 2012;11(2):191-214.
43. Piccini JP, Berger JS, O'Connor CM. Amiodarone for the prevention of sudden cardiac death: a meta-analysis of randomized controlled trials. *European Heart Journal* 2009;30(10):1245-53. doi: 10.1093/eurheartj/ehp100
44. Effect of prophylactic amiodarone on mortality after acute myocardial infarction and in congestive heart failure: meta-analysis of individual data from 6500 patients in randomised trials (Structured abstract). *Lancet* 1997;350(2):1417-24.

For peer review only - http://bmjopen.bmj.com/site/about/guidelines.xhtml

45. Sim I, McDonald KM, Lavori PW, et al. Quantitative overview of randomized trials of amiodarone to prevent sudden cardiac death. *Circulation* 1997;96(9):2823-29.
46. Das MK, Zipes DP. Antiarrhythmic and Nonantiarrhythmic Drugs for Sudden Cardiac Death Prevention. *Journal of Cardiovascular Pharmacology* 2010;55(5):438-49. doi: 10.1097/FJC.0b013e3181d64dde
47. Hilleman DE, Bauman AL. Role of antiarrhythmic therapy in patients at risk for sudden cardiac death: an evidence-based review. *Pharmacotherapy* 2001;21(5):556-75.
48. Rizos E, Ntzani E, Bika E, et al. Association between omega-3 fatty acid supplementation and risk of major cardiovascular disease events: a systematic review and meta-analysis (Structured abstract). *Jama* 2012;308(10):1024-33.
49. Kotwal S, Jun M, Sullivan D, et al. Omega 3 Fatty acids and cardiovascular outcomes: systematic review and meta-analysis. *Circulation Cardiovascular quality and outcomes* 2012;5(6):808-18. doi: 10.1161/circoutcomes.112.966168 [published Online First: 2012/11/01]
50. Kwak SM, Myung SK, Lee YJ, et al. Efficacy of omega-3 fatty acid supplements (eicosapentaenoic acid and docosahexaenoic acid) in the secondary prevention of cardiovascular disease: A meta-analysis of randomized, double-blind, placebo-controlled trials. *Archives of Internal Medicine* 2012;172(9):686-94.
51. Chen Q, Cheng L, Xiao T, et al. Effects of omega-3 fatty acid for sudden cardiac death prevention in patients with cardiovascular disease: a contemporary meta-analysis of randomized, controlled trials (Structured abstract). *Cardiovascular Drugs and Therapy* 2011;25(3):259-65.
52. Marik PE, Varon J. Omega-3 dietary supplements and the risk of cardiovascular events: a systematic review. *Clinical cardiology* 2009;32(7):365-72.
53. Wang C, Harris WS, Chung M, et al. n-3 Fatty acids from fish or fish-oil supplements, but not alpha-linolenic acid, benefit cardiovascular disease outcomes in primary- and secondary-prevention studies: a systematic review. *The American journal of clinical nutrition* 2006;84(1):5-17. [published Online First: 2006/07/11]
54. Bapoje SR, Bahia A, Hokanson JE, et al. Effects of Mineralocorticoid Receptor Antagonists on the Risk of Sudden Cardiac Death in Patients With Left Ventricular Systolic Dysfunction A Meta-analysis of Randomized Controlled Trials. *Circulation-Heart Failure* 2013;6(2):166-+. doi: 10.1161/circheartfailure.112.000003
55. Desai AS, McMurray JJ, Packer M, et al. Effect of the angiotensin-receptor-neprilysin inhibitor LCZ696 compared with enalapril on mode of death in heart failure patients. *European heart journal* 2015;36(30):1990-7. doi: 10.1093/eurheartj/ehv186 [published Online First: 2015/05/30]
56. Sakata Y, Shiba N, Takahashi J, et al. Clinical impacts of additive use of olmesartan in hypertensive patients with chronic heart failure: the supplemental benefit of an angiotensin receptor blocker in hypertensive patients with stable heart failure using olmesartan (SUPPORT) trial. *European heart journal* 2015;36(15):915-23. doi: 10.1093/eurheartj/ehu504 [published Online First: 2015/02/02]
57. Tavazzi L, Maggioni AP, Marchioli R, et al. Effect of n-3 polyunsaturated fatty acids in patients with chronic heart failure (the GISSI-HF trial): a randomised, double-blind, placebo-controlled trial. *Lancet* 2008;372(9645):1223-30. doi: 10.1016/s0140-6736(08)61239-8 [published Online First: 2008/09/02]
58. Shen L, Jhund PS, Petrie MC, et al. Declining Risk of Sudden Death in Heart Failure. *The New England journal of medicine* 2017;377(1):41-51. doi: 10.1056/NEJMoa1609758 [published Online First: 2017/07/06]
59. Busson A, Thilly N, Laborde-Casterot H, et al. Effectiveness of guideline-consistent heart failure drug prescriptions at hospital discharge on 1-year mortality: Results from the EPICAL2 cohort study. *European journal of internal medicine* 2018 doi: 10.1016/j.ejim.2017.12.005 [published Online First: 2018/01/07]
60. Anand K, Mooss AN, Mohiuddin SM. Aldosterone inhibition reduces the risk of sudden cardiac death in patients with heart failure. *Journal of the Renin-Angiotensin-Aldosterone System* 2006;7(1):15-19. doi: 10.3317/jraas.2006.001
61. Rouleau JL, Pfeffer MA, Stewart DJ, et al. Comparison of vasopeptidase inhibitor, omapatrilat, and lisinopril on exercise tolerance and morbidity in patients with heart failure: IMPRESS randomised trial. *Lancet* 2000;356(9230):615-20. [published Online First: 2000/09/01]
62. Packer M, Califf RM, Konstam MA, et al. Comparison of omapatrilat and enalapril in patients with chronic heart failure: the Omapatrilat Versus Enalapril Randomized Trial of Utility in Reducing Events (OVERTURE). *Circulation* 2002;106(8):920-6. [published Online First: 2002/08/21]
63. Doggrell SA. Amiodarone - Waxed and waned and waxed again. *Expert Opinion on Pharmacotherapy* 2001;2(11):1877-90.

For peer review only - http://bmjopen.bmj.com/site/about/guidelines.xhtml

64. Ponikowski P, Voors AA, Anker SD, et al. 2016 ESC Guidelines for the diagnosis and treatment of acute and chronic heart failure: The Task Force for the diagnosis and treatment of acute and chronic heart failure of the European Society of Cardiology (ESC). Developed with the special contribution of the Heart Failure Association (HFA) of the ESC. *European journal of heart failure* 2016;18(8):891-975. doi: 10.1002/ejhf.592 [published Online First: 2016/05/22]
65. Al-Gobari M, Le HH, Fall M, et al. No benefits of statins for sudden cardiac death prevention in patients with heart failure and reduced ejection fraction: A meta-analysis of randomized controlled trials. *PloS one* 2017;12(2):e0171168. doi: 10.1371/journal.pone.0171168 [published Online First: 2017/02/07]
66. Guyatt GH, Oxman AD, Vist GE, et al. GRADE: an emerging consensus on rating quality of evidence and strength of recommendations. *BMJ : British Medical Journal* 2008;336(7650):924-26. doi: 10.1136/bmj.39489.470347.AD

For peer review only

Legends of figures

Fig 1: flow chart for search result

Fig 2: Efficacy of angiotensin receptor blockers (ARBs) compared with placebo**,** angiotensin-converting enzyme inhibitor (ACE-i), or combined in heart failure and reduced ejection fraction (HFrEF) for the prevention of **all-cause mortality**.

Fig 3: Efficacy of angiotensin receptor blockers (ARBs) compared with placebo**,** angiotensin-converting enzyme inhibitor (ACE-i), or combined in heart failure and reduced ejection fraction (HFrEF) for the prevention of **sudden cardiac death (SCD).**

Fig 4: Funnel plot of SE (log odds ratio) by odds ratio to evaluate publication bias for the efficacy of angiotensin receptor blockers (ARBs) compared with control in heart failure and reduced ejection fraction (HFrEF) for the prevention of **all-cause mortality.**

For peer review only - http://bmjopen.bmj.com/site/about/guidelines.xhtml
